# Once-Daily Oral Ozanimod for Japanese Patients With Ulcerative Colitis: Results From the Phase 2/3 J-True North Study

**DOI:** 10.1016/j.gastha.2025.100812

**Published:** 2025-09-16

**Authors:** Hiroshi Nakase, Toshimitsu Fujii, Tadakazu Hisamatsu, Yasuo Suzuki, Mamoru Watanabe, Sakuma Takahashi, Makoto Ooi, Ken Takeuchi, Tsuguhiro Kimura, Ken Furuya, Nobuo Aoyama, Kenkei Hasatani, Noriyuki Horiki, Kazunari Kanke, Satoki Tokito, Souken Sai, Yoko Uchikawa, Shoichiro Goto, Go Fujimoto, Changliang Zhang, AnnKatrin Petersen, Toshifumi Hibi

**Affiliations:** 1 Sapporo Medical University, Sapporo, Japan; 2 Institute of Science Tokyo, Tokyo, Japan; 3 Kyorin University School of Medicine, Mitaka, Japan; 4 Ginza Central Clinic, Tokyo, Japan; 5 Graduate School of Medicine, Juntendo University, Tokyo, Japan; 6 Kagawa Prefectural Central Hospital, Takamatsu, Japan; 7 Division of Gastroenterology, Department of Internal Medicine, Graduate School of Medicine, Kobe University, Kobe, Japan; 8 Tsujinaka Hospital Kashiwanoha, Kashiwa, Japan; 9 Medical Corporation Shoyu-Kai Fujita Gastroenterology Hospital, Takatsuki, Japan; 10 JCHO Hokkaido Hospital, Sapporo, Japan; 11 Aoyama Medical Clinic GI Endoscopy & IBD Center, Kobe, Japan; 12 Fukui Prefectural Hospital, Fukui, Japan; 13 Mie University Hospital, Tsu, Japan; 14 Kanke Gastrointestinal Clinic, Utsunomiya, Japan; 15 Tokitokai Tokito Clinic, Saitama, Japan; 16 Sai Gastroenterology and Proctology Clinic, Fujiidera, Japan; 17 Bristol Myers Squibb, Tokyo, Japan; 18 Bristol Myers Squibb, Princeton, New Jersey; 19 Kitasato University Kitasato Institute Hospital, Tokyo, Japan

**Keywords:** Clinical Trial, Japan, Ozanimod, Sphingosine 1-Phosphate, Ulcerative Colitis

## Abstract

**Background and Aims:**

Ozanimod is a once-daily, oral, selective sphingosine 1-phosphate receptor 1 and 5 modulator. The objective of the randomized, phase 2/3 J-True North study (NCT03915769) was to assess the efficacy and safety of ozanimod in Japanese patients with moderately to severely active ulcerative colitis.

**Methods:**

In the 12-week induction period (IP), patients were randomized 1:1:1 to receive placebo, ozanimod 0.46 mg, or ozanimod 0.92 mg. Patients who completed the IP with a clinical response at week (w) 12 were eligible to enter a 40-week maintenance period where they received the same treatment as they did in the IP. The primary endpoint was clinical response (complete Mayo score) at w12; clinical and mucosal secondary endpoints were assessed at w12 and w52.

**Results:**

Of 198 patients randomized, 176 completed the IP. Of these patients, 97 entered and 77 completed the maintenance period. A significantly higher proportion of patients receiving ozanimod achieved clinical response at w12 versus placebo (ozanimod 0.46 mg: 52.9%, *P* = .0158; ozanimod 0.92 mg: 61.5%, *P* = .0006; vs placebo: 32.3%). Similar results were observed in the secondary endpoints where patients receiving ozanimod achieved higher rates of clinical remission, endoscopic improvement, and mucosal healing at w12 than those receiving placebo. Efficacy was maintained at w52 for all endpoints. Both doses of ozanimod were well tolerated, with no unexpected safety signals.

**Conclusion:**

This large-scale clinical trial demonstrated the efficacy and safety of once-daily oral ozanimod in Japanese patients with moderately to severely active ulcerative colitis. This is the first time that the efficacy and safety of ozanimod were verified in a large number of patients in Asia.

## Introduction

Ulcerative colitis (UC) is an immune-mediated disease with unknown etiology characterized by an increased migration and accumulation of lymphocytes in the inflamed tissues of the colon and rectum.^1^^,^^2^ Although UC has a higher prevalence in the West, it is increasing in Asia, especially in Japan.^3^

Patients with UC are commonly treated with standard therapies (eg, 5-aminosalicylic acid, corticosteroids, immunomodulators), often followed by advanced therapies (ie, biologics [eg, anti-tumor necrosis factor agents, anti-integrins, anti-interleukin 12/23], and Janus kinase [JAK] inhibitors) if UC is uncontrolled with standard treatment.^1^^,^^4^, ^5^, ^6^ Despite the advancements in treatments for UC, there is still a need for novel advanced therapies due to the overuse of corticosteroids and parenteral administration, limited efficacy (ie, primary nonresponse or loss of response over time), and safety profiles associated with current advanced therapies.^7^, ^8^, ^9^, ^10^, ^11^, ^12^, ^13^, ^14^, ^15^

Unlike most advanced therapies that target cytokine signaling,^16^, ^17^, ^18^ sphingosine 1-phosphate (S1P) receptor modulators, such as ozanimod, primarily regulate lymphocyte trafficking.^2^^,^^19^ Ozanimod is an oral small molecule that selectively binds to S1P_1_ and S1P_5_ receptors, causing internalization of S1P_1_ receptors on lymphocyte surfaces to prevent S1P-dependent lymphocyte egression from lymph nodes to inflamed tissues.^2^ Given the role of S1P receptors on lymphocyte trafficking, targeting these receptors has proven effective in treating immune-mediated diseases, such as multiple sclerosis (MS) and UC.^2^^,^^20^^,^^21^ The efficacy and/or safety of ozanimod 0.92 mg for the treatment of moderately to severely active UC was demonstrated in the phase 2 TOUCHSTONE study^20^ and the subsequent pivotal phase 3 True North study.^22^ Ozanimod was approved for the treatment of relapsing MS in 2020 and moderately to severely active UC in 2021 in the United States and several other countries.^23^, ^24^, ^25^, ^26^, ^27^, ^28^

This phase 2/3 Japan (J)-True North study compared the efficacy and safety of once-daily ozanimod 0.46 or 0.92 mg with placebo in Japanese patients with moderately to severely active UC. Based on the findings from J-True North, once-daily ozanimod 0.92 mg was approved for the treatment of UC in Japan in December 2024.^29^ Herein, we report the J-True North efficacy and safety results for ozanimod in Japanese patients with moderately to severely active UC.

## Methods

### Patients

Japanese patients aged 18–75 years with a diagnosis of UC ≥3 months before receiving study treatment, evidence of UC extending ≥15 cm from the anal verge by baseline endoscopy, and moderately to severely active UC (defined as Mayo score of 6–12 with a Mayo endoscopy subscore ≥2, a rectal bleeding subscore [RBS] ≥1, and a stool frequency subscore [SFS] ≥1) were included. Patients must have been previously exposed to aminosalicylates or corticosteroids, and those receiving treatment of either at enrollment needed to continue treatment during the induction period. In addition, patients needed to have documentation of positive varicella zoster virus immunoglobulin G (IgG) antibody status or must have completed varicella zoster virus vaccination ≥30 days before randomization. Patients were excluded if they had severe extensive colitis, Crohn’s disease or intermediate colitis, clinically relevant cardiovascular conditions, a history of type 1 diabetes or uncontrolled type 2 diabetes, or a history of uveitis or macular edema. Full inclusion and exclusion criteria are included in the Supplementary Methods.

### Study Design

J-True North was a multicenter, double-blind, placebo-controlled, parallel-group, 2-dose, randomized phase 2/3 study. A list of J-True North investigators, sites, institutional review boards or ethical review boards, and chairpersons are reported in Table A1.

Following up to 5 weeks of screening, patients were randomized 1:1:1 using interactive-response technology to receive once-daily ozanimod 0.46 mg, ozanimod 0.92 mg, or placebo, stratified by corticosteroid use at screening and prior use of biologics, in the 12-week induction period. The study included patients with or without prior biologic exposure, with the proportion of those previously treated with biologics limited to approximately 30%. Patients randomized to ozanimod initiated a 7-day dose escalation upon treatment initiation: ozanimod 0.23 mg on days 1–4, ozanimod 0.46 mg on days 5–7, and the assigned ozanimod treatment dose (0.46 mg or 0.92 mg) thereafter. Patients who achieved clinical response at week 12 were eligible to enter the 40-week maintenance period, during which patients continued to receive the same treatment. Patients were eligible to enter an open-label extension (OLE) to receive once-daily ozanimod 0.92 mg if they were clinical nonresponders at week 12, experienced disease relapse during the maintenance period, or completed the maintenance period (Figure A1, Supplementary Methods).

The patient, investigator, and study-site personnel were blinded to the treatment received by each patient. A patient’s treatment assignment was kept blinded to the investigators until after the last randomized patient completed their last visit in the maintenance period, unless subsequent medical treatment for the patient required knowledge of the assigned treatment.

### Assessments and Outcomes

#### Efficacy

Endoscopy and biopsy samples were evaluated in a blinded manner by a qualified central laboratory. Patient-reported outcomes (ie, SFS and RBS) and the clinician-reported Physician Global Assessment were collected in an electronic diary. All efficacy endpoints are defined in Table A2.

The primary endpoint was the proportion of patients with clinical response (ie, a reduction from baseline in the complete Mayo score [sum of SFS, RBS, endoscopy subscore, and Physician Global Assessment, with each assessment rated on a scale of 0–3] ≥3 points and ≥30%, and a reduction from baseline in the RBS of ≥1 or an absolute RBS of ≤1 point) at week 12.

Secondary efficacy endpoints, listed in no particular order, included the proportion of patients who achieved clinical response (complete Mayo score) at week 52 and the proportion of patients who achieved clinical response (9-point Mayo score), clinical remission (based on definitions 1 and 2 in Table A2), endoscopic improvement, and mucosal healing at weeks 12 and 52. The proportion of patients who achieved histologic remission and clinical remission (based on definition 3 in Table A2) at weeks 12 and 52 were also explored.

### Pharmacodynamics

Absolute lymphocyte count (ALC), C-reactive protein (CRP), and stool analysis for fecal calprotectin (FCP) were assessed throughout the study.

### Safety

Treatment-emergent adverse events (TEAEs), serious adverse events (SAEs), TEAEs leading to discontinuation, and adverse events of special interest (eg, bradycardia, heart conduction abnormalities, serious infections, malignancies, macular edema, hepatic effects) were assessed through J-True North. Clinical laboratory evaluations were completed by a central laboratory. During the blinded treatment period, white blood cell differential results were available to an unblinded medical reviewer independent from the study. Vital signs, electrocardiograms (before first dose and 6 hours after the first dose), and optical coherence tomography (for at-risk patients with a history of uveitis, diabetes mellitus, or underlying or coexisting retinal disease) were also completed during the study.

### Statistical Analysis

Baseline patient characteristics and demographics were summarized descriptively. The intention-to-treat population, which was the primary population for efficacy analyses, included all randomized patients from the screened population who received ≥1 dose of study treatment. The primary endpoint of clinical response at week 12 was analyzed using the Cochran-Mantel-Haenszel test stratified by corticosteroid use at screening and by prior biologic use. Pairwise comparisons were performed between each ozanimod group and the placebo group. Other binary efficacy endpoints were similarly analyzed. Missing data were handled using nonresponder imputation analyses, which included patients who discontinued the study before a given timepoint and patients who met prespecified criteria for treatment failures before the timepoint and were considered nonresponders at that timepoint. Continuous endpoints were analyzed by analysis of covariance models adjusted for corticosteroid use (yes or no) at screening, prior biologic use (yes or no), and the baseline value of the corresponding variable.

To account for multiplicity for the primary endpoint, ozanimod 0.92 mg versus placebo comparison in the primary analysis was tested using a 2-sided test with an alpha of 0.05 level of significance. When this comparison was statistically significant (*P* ≤ .05), ozanimod 0.46 mg versus placebo comparisons were further performed. Due to no statistical hypotheses, secondary and exploratory efficacy endpoints were tested in a nonhierarchical fashion without multiplicity adjustment; therefore, reported *P* values were nominal and were provided as a measure of strength of association between the endpoint and treatment effect for the exploratory purpose. By using a 2-sided chi-square test for 2-sample hypothesis testing at an alpha of 0.05 with a 90% power, the estimated sample size needed to detect a difference of 28% between ozanimod 0.92 mg versus placebo was 65 patients per treatment group, or a total of 195 patients.

### Ethical Considerations

J-True North adhered to the Good Clinical Practices guidelines and was conducted in accordance with the ethical principles outlined in the Declaration of Helsinki. Before study initiation, the study protocol and informed consent were approved at each study site by an institutional review board or independent ethics committee. Written informed consent was obtained from each patient prior to entering the study and before the initiation of any trial-related procedure. J-True North was sponsored by Bristol Myers Squibb.

## Results

### Patient Disposition and Baseline Characteristics

**Figure 1:**
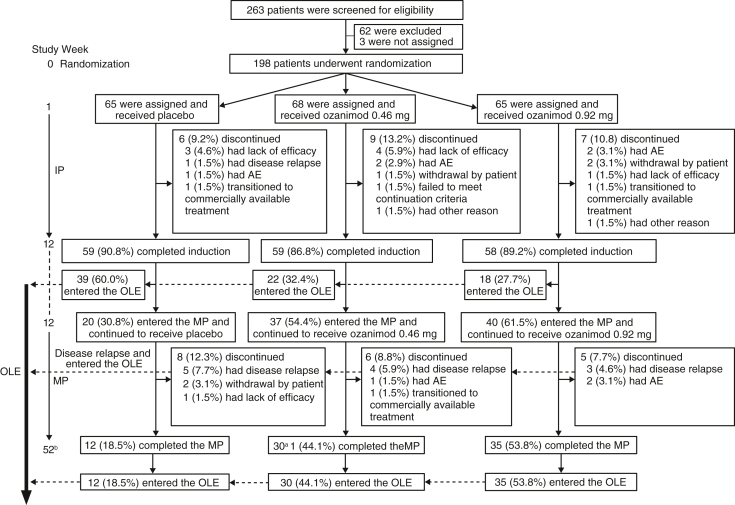
Patient disposition. Percentages are based on the randomized population. ^a^One patient completed the week 52 study visit but was still on treatment in the MP at the time of the data cutoff date; all data for this patient up to week 52 were included in the analysis. ^b^Duration of the MP was shortened to 40 weeks (total of 52 weeks) after the study protocol was amended from the original 52 weeks (total of 64 weeks). The timepoint of MP efficacy endpoints was week 52 throughout the study. AE, adverse event; IP, induction period; MP, maintenance period; OLE, open-label extension.

From June 3, 2019, to August 28, 2023, a total of 263 patients were enrolled and screened (Figure 1). Of these patients, 198 were randomized: 65 to placebo, 68 to ozanimod 0.46 mg, and 65 to ozanimod 0.92 mg. Most patients (n = 176 [88.9%]) completed the 12-week induction period (placebo: n = 59 [90.8%]; ozanimod 0.46 mg: n = 59 [86.8%]; ozanimod 0.92 mg: n = 58 [89.2%]). The most common reasons for treatment discontinuation in the induction period were lack of efficacy (n = 8 [4.0%]), adverse event (AE; n = 5 [2.5%]), and withdrawal by patient (n = 3 [1.5%]).

A total of 97 (49.0%) patients who achieved clinical response at week 12 entered the maintenance period (placebo: n = 20 [30.8%]; ozanimod 0.46 mg: n = 37 [54.4%]; ozanimod 0.92 mg: n = 40 [61.5%]). Of these patients, 77 completed the maintenance period (placebo: n = 12 [18.5%]; ozanimod 0.46 mg: n = 30 [44.1%]; ozanimod 0.92 mg: n = 35 [53.8%]). For the 19 patients who discontinued treatment in the placebo or ozanimod groups, common reasons for discontinuation included disease relapse (n = 12 [6.1%]), AE (n = 3 [1.5%]), and withdrawal by patient (n = 2 [1.0%]). At the time of data cutoff (August 28, 2023), 168 patients entered the OLE: 79 (47.0%) from the induction period, 12 (7.1%) who relapsed during the maintenance period, and 77 (45.8%) who completed the maintenance period.

The baseline demographic and disease characteristics were well balanced across the 3 treatment groups (Table 1). Overall, more patients were male, the mean age was approximately 43 years, the mean total Mayo score at baseline was 8.4, and 21.2% of patients were previously exposed to biologics.

**Table 1:** Demographic and Clinical Characteristics at Baseline in the Induction Period (Intention-to-Treat Population)

Characteristic	Placebo (N = 65)	Ozanimod 0.46 mg (N = 68)	Ozanimod 0.92 mg (N = 65)	Total (N = 198)
Female, n (%)	26 (40.0)	22 (32.4)	24 (36.9)	72 (36.4)
Age, y, mean (SD)	42.5 (13.0)	43.9 (13.0)	41.4 (14.3)	42.6 (13.4)
Weight, kg, mean (SD)	62.5 (11.4)	62.7 (11.8)	62.8 (11.5)	62.7 (11.5)
BMI, kg/m^2^, mean (SD)	22.7 (2.8)	22.5 (3.3)	22.5 (3.5)	22.6 (3.2)
Years since UC diagnosis, mean (SD)	8.3 (7.4)	7.0 (8.3)	6.3 (6.5)	7.2 (7.5)
Extent of UC disease, n (%)				
Left-sided	27 (41.5)	37 (54.4)	26 (40.0)	90 (45.5)
Extensive	38 (58.5)	31 (45.6)	39 (60.0)	108 (54.5)
Total mayo score, mean (SD)	8.5 (1.1)	8.4 (1.4)	8.3 (1.5)	8.4 (1.3)
9-Point mayo score,^a^ mean (SD)	6.4 (1.0)	6.4 (1.2)	6.3 (1.4)	6.4 (1.2)
Fecal calprotectin, μg/g, median (range)	1060 (13–16,800)	1500 (27–15,200)	885 (20–22,200)	1130 (13–22,200)
Prior medication use, n (%)				
5-ASA	65 (100.0)	68 (100.0)	65 (100.0)	198 (100.0)
Corticosteroid	57 (87.7)	58 (85.3)	52 (80.0)	167 (84.3)
Immunomodulator	27 (41.5)	23 (33.8)	25 (38.5)	75 (37.9)
Biologics	14 (21.5)	15 (22.1)	13 (20.0)	42 (21.2)
Nonresponse to biologics,^b^ n (%)				
Primary nonresponse	5 (7.7)	4 (5.9)	6 (9.2)	15 (7.6)
Secondary nonresponse	4 (6.2)	10 (14.7)	6 (9.2)	20 (10.1)

5-ASA, 5-aminosalicylic acid; BMI, body mass index; RBS, rectal bleeding subscore; SD, standard deviation; SFS, stool frequency subscore; UC, ulcerative colitis.

a The sum of RBS, SFS, and Mayo endoscopy subscore.

b Percentages are from the total patients in each treatment group.

### Efficacy Outcomes in the Induction Period

**Figure 2:**
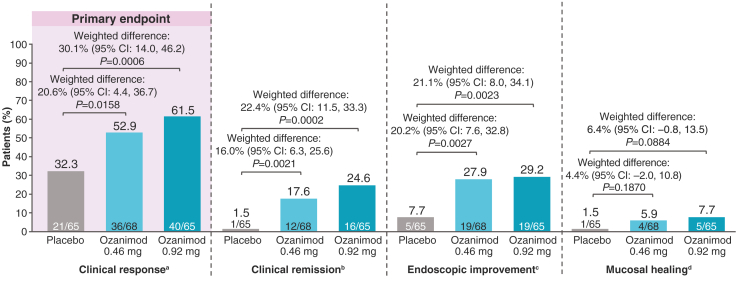
Proportion of patients who achieved efficacy endpoints at week 12. Nonresponder imputation approach was used for handling of missing data. Weighted differences, 95% CIs, and *P* values for comparison between groups were based on the Cochran-Mantel-Haenszel test and were stratified by prior biologic agents and corticosteroid use (yes/no). ^a^Clinical response: reduction from baseline in total Mayo score of ≥3 points and ≥30%, and reduction from baseline in the RBS of ≥1 point or an absolute RBS of ≤1 point. ^b^Clinical remission: RBS = 0 and SFS ≤1 (and a decrease of ≥1 point from baseline SFS) and MES ≤1 point. ^c^Endoscopic improvement: MES ≤1 point. ^d^Mucosal healing: MES ≤1 point and Geboes score <2.0. CI, confidence interval; MES, Mayo endoscopy subscore; RBS, rectal bleeding subscore; SFS, stool frequency subscore.

At week 12, the percentages of patients who achieved clinical response (complete Mayo score), the primary endpoint, were significantly higher in both ozanimod groups (ozanimod 0.46 mg: 52.9%, *P* = .0158; ozanimod 0.92 mg: 61.5%, *P* = .0006) than in the placebo group (32.3%) (Figure 2). Greater proportions of patients achieved clinical remission (ozanimod 0.46 mg: 17.6%, *P* = .0021; ozanimod 0.92 mg: 24.6%, *P* = .0002; vs placebo: 1.5%) and endoscopic improvement (ozanimod 0.46 mg: 27.9%, *P* = .0027; ozanimod 0.92 mg: 29.2%, *P* = .0023; vs placebo: 7.7%) with ozanimod than placebo at week 12 (Figure 2). Likewise, higher proportions of patients achieved mucosal healing at week 12 with ozanimod versus placebo (ozanimod 0.46 mg: 5.9%, *P* = .1870; ozanimod 0.92 mg: 7.7%, *P* = .0884; vs placebo: 1.5%) (Figure 2).

The efficacy of ozanimod at week 12 remained consistent when using alternate definitions of clinical response and clinical remission (Figure A2A). A higher proportion of patients achieved histologic remission at week 12 with both ozanimod dose groups compared with placebo (ozanimod 0.46 mg: 11.8%, *P* = .0183; ozanimod 0.92 mg: 13.8%, *P* = .0071; vs placebo: 1.5%) (Figure A2A).

In a prespecified subgroup analysis of the primary endpoint, ozanimod 0.92 mg was effective regardless of prior biologic experience or concomitant corticosteroid use; however, those without concomitant corticosteroid use demonstrated better efficacy than those with concomitant corticosteroid use (Figure A3A). Clinical response rates favored ozanimod 0.92 mg over placebo in most baseline characteristic subgroups (Figure A3A), and similar patterns were seen in the ozanimod 0.46 mg group (Figure A3B).

### Efficacy Outcomes in the Maintenance Period

**Figure 3:**
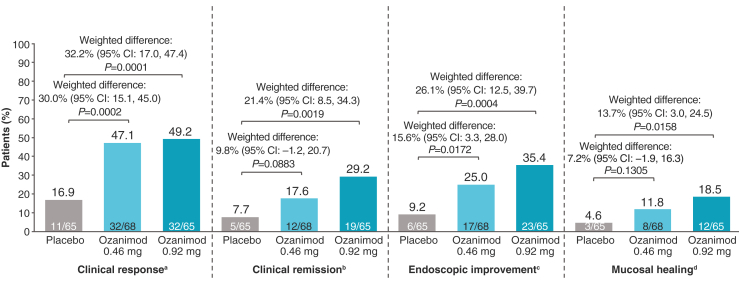
Proportions of patients who achieved efficacy endpoints at week 52. Nonresponder imputation approach was used for handling of missing data. Weighted differences, 95% CIs, and *P* values for comparison between groups were based on the Cochran-Mantel-Haenszel test and were stratified by prior biologic agents and corticosteroid use (yes/no). ^a^Clinical response: reduction from baseline in total Mayo score of ≥3 points and ≥30%, and reduction from baseline in the RBS of ≥1 point or an absolute RBS of ≤1 point. ^b^Clinical remission: RBS = 0 and SFS ≤1 (and a decrease of ≥1 point from baseline SFS) and MES ≤1 point. ^c^Endoscopic improvement: MES ≤1 point. ^d^Mucosal healing: MES ≤1 point and Geboes score <2.0. CI, confidence interval; MES, Mayo endoscopy subscore; RBS, rectal bleeding subscore; SFS, stool frequency subscore.

Compared with placebo, a higher proportion of patients in both ozanimod groups achieved clinical response at week 52 in the maintenance period (ozanimod 0.46 mg: 47.1%, *P* = .0002; ozanimod 0.92 mg: 49.2%, *P* = .0001; vs placebo: 16.9%) (Figure 3). Notably, ≥80% of patients who achieved clinical response receiving ozanimod 0.46 or ozanimod 0.92 mg at the end of the induction period also showed clinical response at the end of the maintenance period.

At week 52, rates of clinical remission were greater with ozanimod 0.46 mg (17.6%, *P* = .0883) and with ozanimod 0.92 mg (29.2%, *P* = .0019) than with placebo (7.7%) (Figure 3). A similar pattern was seen for endoscopic improvement and mucosal healing (Figure 3). These results were consistent when measured by alternate definitions of clinical response and remission (Figure A2B). Rates of histologic remission were greater with ozanimod than placebo at week 52 (ozanimod 0.46 mg: 17.6%, *P* = .0176; ozanimod 0.92 mg: 24.6%, *P* = .0016; vs placebo: 4.6%) (Figure A2B). Although sample sizes were low, corticosteroid-free remission at week 52 was achieved in 0% (0/3), 25.0% (1/4), and 28.6% (2/7) of patients in the placebo, ozanimod 0.46 mg, and ozanimod 0.92 mg groups, respectively.

### Pharmacodynamics

Median FCP levels at baseline were 1060 μg/g in the placebo group, 1500 μg/g in the ozanimod 0.46 mg group, and 885 μg/g in the ozanimod 0.92 mg group. Greater decreases from baseline in FCP levels were seen with ozanimod treatment versus placebo at weeks 12 and 52 (Table A3). The proportions of patients whose FCP levels shifted from baseline to ≤50 μg/g, ≤100 μg/g, and ≤150 μg/g at week 12 were higher in both ozanimod groups compared with the placebo group (Table A4). Median CRP levels at baseline were similar in the 3 groups: placebo (1.4 mg/L), ozanimod 0.46 mg (1.7 mg/L), and ozanimod 0.92 mg (1.6 mg/L). Similar to FCP, greater decreases from baseline in CRP levels were seen with ozanimod than with placebo at weeks 12 and 52 (Table A3).

While ALCs were stable in the placebo group, those in the ozanimod 0.46 mg and 0.92 mg groups showed a decrease at week 2 (mean ALC change from baseline: −0.6 × 10^9^/L and −0.7 × 10^9^/L, respectively) and a plateau at week 5 (−0.8 × 10^9^/L and −0.8 × 10^9^/L) (Figure A4). The decreases in ALC observed during the induction period in both ozanimod groups were sustained throughout the maintenance period. Mean ALC decreased by 1.0 × 10^9^/L in both ozanimod groups compared with an increase of 0.08 × 10^9^/L in the placebo group from baseline to week 52. Leukocytes decreased by 0.2 × 10^9^/L in the placebo group, 1.9 × 10^9^/L in the ozanimod 0.46 mg group, and 2.0 × 10^9^/L in the ozanimod 0.92 mg group from baseline to week 52 (Table A5). Slight decreases were seen for circulating neutrophils (placebo: 0.3 × 10^9^/L; ozanimod 0.46 mg: 0.6 × 10^9^/L; ozanimod 0.92 mg: 0.9 × 10^9^/L). Only 1 patient (1.5%) in the placebo group had a total white blood cell count >20 × 10^9^/L after the initiation of treatment (Table A6).

### Safety

The overall incidences of AEs were similar across all treatment groups in the induction period and greater in the ozanimod groups than in the placebo group during the induction and maintenance periods (Table 2). The incidences of SAEs in the induction and maintenance periods were 2 (3.1%) in the placebo group, 5 (7.4%) in the ozanimod 0.46 mg group, and 5 (7.7%) in the ozanimod 0.92 mg group. Treatment-related SAEs occurred in 1 patient in the placebo group and 1 patient in the ozanimod 0.46 mg group. TEAEs leading to treatment withdrawal occurred in 5.9% and 6.2% in the ozanimod 0.46 mg and 0.92 mg groups, respectively, and 3.1% in the placebo group in the induction and maintenance periods. TEAEs leading to ozanimod treatment withdrawal in the induction and maintenance periods included hemiplegia (1.5% [1/68]) in the ozanimod 0.46 mg group, macular edema (1.5% [1/65]) in the ozanimod 0.92 mg group, UC (2.9% [2/68]) in the ozanimod 0.46 mg group, drug-induced liver injury (1.5% [1/65]) in the ozanimod 0.92 mg group, pyrexia (1.5% [1/68]) in the ozanimod 0.46 mg group, alanine aminotransferase increased (3.1% [2/65]) in the ozanimod 0.92 mg group, and aspartate aminotransferase increased (3.1% [2/65]) in the ozanimod 0.92 mg group. The overall incidences of nonserious infections were higher in the placebo group than in the ozanimod groups in the induction period and similar between groups in the induction and maintenance periods. Only 1 serious infection occurred in the induction and maintenance periods: a patient with COVID-19 in the ozanimod 0.92 mg group. There were no deaths during the induction and maintenance periods (Table 2). TEAEs with incidence >2% are listed in Table A7.

**Table 2:** Safety Findings in the Induction and Maintenance Periods

TEAEs	IP			IP and MP		
	Placebo (n = 65)	Ozanimod 0.46 mg (n = 68)	Ozanimod 0.92 mg (n = 65)	Placebo (n = 65)	Ozanimod 0.46 mg (n = 68)	Ozanimod 0.92 mg (n = 65)
≥1 TEAE	36 (55.4)	40 (58.8)	38 (58.5)	43 (66.2)	51 (75.0)	51 (78.5)
≥1 serious TEAE	2 (3.1)	4 (5.9)	4 (6.2)	2 (3.1)	5 (7.4)	5 (7.7)
≥1 serious TEAE related to treatment	1 (1.5)	1 (1.5)	0	1 (1.5)	1 (1.5)	0
≥1 serious TEAE leading to treatment withdrawal	2 (3.1)	3 (4.4)	3 (4.6)	2 (3.1)	4 (5.9)	4 (6.2)
TEAE with incidence ≥5%	1 (1.5)	1 (1.5)	3 (4.6)	1 (1.5)	1 (1.5)	4 (6.2)
TEAE with incidence ≥5%						
Nasopharyngitis	4 (6.2)	5 (7.4)	5 (7.7)	6 (9.2)	10 (14.7)	9 (13.8)
Pyrexia	2 (3.1)	6 (8.8)	2 (3.1)	3 (4.6)	11 (16.2)	7 (10.8)
Headache	4 (6.2)	4 (5.9)	4 (6.2)	4 (6.2)	8 (11.8)	6 (9.2)
Back pain	5 (7.7)	2 (2.9)	3 (4.6)	5 (7.7)	5 (7.4)	6 (9.2)
COVID-19	2 (3.1)	2 (2.9)	1 (1.5)	3 (4.6)	4 (5.9)	5 (7.7)
Colitis ulcerative	1 (1.5)	4 (5.9)	4 (6.2)	1 (1.5)	4 (5.9)	4 (6.2)
Arthralgia	1 (1.5)	2 (2.9)	3 (4.6)	2 (3.1)	4 (5.9)	4 (6.2)
Abdominal pain	1 (1.5)	2 (2.9)	0	1 (1.5)	4 (5.9)	2 (3.1)
GGT increased	0	1 (1.5)	1 (1.5)	0	5 (7.4)	2 (3.1)
Dental caries	1 (1.5)	3 (4.4)	0	2 (3.1)	5 (7.4)	0
Infection with incidence ≥3%						
Nasopharyngitis	4 (6.2)	5 (7.4)	5 (7.7)	6 (9.2)	10 (14.7)	9 (13.8)
COVID-19	2 (3.1)	2 (2.9)	1 (1.5)	3 (4.6)	4 (5.9)	5 (7.7)^a^
Herpes zoster	1 (1.5)	0	1 (1.5)	1 (1.5)	1 (1.5)	2 (3.1)
Hordeolum	0	0	0	2 (3.1)	1 (1.5)	0
Serious infection	0	0	0	0	0	1 (1.5)
Cancer	0	0	0	0	0	0
AESI^b^	2 (3.1)	1 (1.5)	2 (3.1)	2 (3.1)	3 (4.4)	5 (7.7)
Bradycardia	0	0	0	0	0	0
Herpes zoster	1 (1.5)	0	1 (1.5)	1 (1.5)	1 (1.5)	2 (3.1)
Macular edema	0	0	0	0	0	1 (1.5)
Orthostatic hypotension	0	0	0	0	0	1 (1.5)
Cytomegalovirus colitis	0	1 (1.5)	0	0	1 (1.5)	0
Oral herpes	0	0	0	0	1 (1.5)	0
Drug-induced liver injury^c^	0	0	1 (1.5)	0	0	1 (1.5)
Interstitial lung disease	1 (1.5)	0	0	1 (1.5)	0	0
Laboratory assessments						
ALT^d^						
≥2 × ULN	0	3 (4.5)	3 (4.8)	1 (1.6)	9 (13.6)	5 (7.9)
≥3 × ULN	0	0	2 (3.2)	1 (1.6)	1 (1.5)	2 (3.2)
≥5 × ULN	0	0	1 (1.6)	0	1 (1.5)	1 (1.6)
≥10 × ULN	0	0	1 (1.6)^b^	0	0	1 (1.6)^b^
AST^d^						
≥2 × ULN	0	1 (1.5)	2 (3.2)	0	3 (4.5)	3 (4.8)
≥3 × ULN	0	1 (1.5)	2 (3.2)	0	2 (3.0)	2 (3.2)
≥5 × ULN	0	0	2 (3.2)	0	1 (1.5)	2 (3.2)
≥10 × ULN	0	0	0	0	0	0

AESI, adverse event of special interest; ALT, alanine aminotransferase; AST, aspartate aminotransferase; GGT, gamma-glutamyl transferase; IP, induction period; MP, maintenance period; PML, progressive multifocal leukoencephalopathy; TEAE, treatment-emergent adverse event; ULN, upper limit of normal.

a 1 COVID-19 adverse event that occurred during the MP required hospitalization.

b Bradycardia (defined as events that were symptomatic, had a heart rate <45 beats per minute, or required treatment), heart conduction abnormalities (ie, second-degree and higher atrioventricular block), macular edema, malignancy, serious or opportunistic infection, pulmonary effects, hepatic effects, posterior reversible encephalopathy syndrome, PML, and events associated with orthostatic hypotension (eg, dizziness, lightheadedness, fainting, syncope, seizure) were defined as AESIs.

c A patient who had a medical history of hepatic steatosis and hyperlipidemia and concomitantly used rosuvastatin and ezetimibe experienced ALT >10 × ULN. The patient discontinued ozanimod as this event met the discontinuation criteria per protocol but total bilirubin remained within the normal range; the patient did not meet Hy’s law criteria, and no clinical symptoms were observed. There was no treatment or intervention for this event, and the event was considered to be related to study treatment.

d Percentages were based on the numbers of patients with assessments: placebo = 64, ozanimod 0.46 mg = 66, and ozanimod 0.92 mg = 63.

In the induction and maintenance periods, the incidence of adverse events of special interest was 3.1% (2/65) in the placebo group, 4.4% (3/68) in the ozanimod 0.46 group, and 7.7% (5/65) in the ozanimod 0.92 mg group. Orthostatic hypotension occurred in 1 patient in the ozanimod 0.92 mg group, and the event was not serious, was mild in severity, and resolved. Macular edema occurred in 1 patient in the ozanimod 0.92 mg group who discontinued treatment due to the event; the event was not serious, was mild in severity, and resolved. Incidences of herpes zoster infection were low and similar across treatment groups; herpes zoster infection occurred in 1 patient each in the placebo group and ozanimod 0.46 mg group and in 2 patients in the ozanimod 0.92 mg group. All cases of herpes zoster resolved while on study treatment. There was 1 case each of cytomegalovirus colitis and oral herpes, both of which occurred in the ozanimod 0.46 mg group and subsequently resolved. There were no AEs related to pulmonary effects with ozanimod treatment. There were no cases of cancer, bradycardia, or progressive multifocal leukoencephalopathy during the study (Table 2).

Elevated liver transaminase levels were more common in the ozanimod treatment groups than in the placebo group (Table 2). No patients with alanine aminotransferase or aspartate aminotransferase >3 × the upper limit of normal elevation experienced accompanying symptoms suggestive of liver injury. Drug-induced liver injury occurred in 1 patient who received ozanimod 0.92 mg, which led to drug withdrawal, but did not meet the criteria for Hy’s law (defined as alanine aminotransferase or aspartate aminotransferase levels ≥3 × the upper limit of normal and a total bilirubin >2 × the upper limit of normal)^30^ as the patient had a normal total bilirubin despite an alanine aminotransferase level >10 × the upper limit of normal; this event was considered related to study treatment. No Hy’s law cases or serious hepatic AEs were associated with hepatic abnormalities.

ALC reductions <500 cells/μL occurred in 3.1% (2/65), 40.3% (27/67), and 58.5% (38/65) of patients in the induction period. An increase in the number of patients with ALC <500 cells/μL was observed in the ozanimod treatment groups when combining the induction and maintenance period to 3.1% (2/65), 52.2% (35/67), and 67.7% (44/65) of patients with an assessment per visit receiving placebo, ozanimod 0.46 mg, and ozanimod 0.92 mg, respectively. Patients who experienced ALC reduction <200 cells/μL had a recovery time to ALC levels >200 cells/μL after treatment interruption within 2 weeks, while no treatment interruption was required for patients with ALC <500 cells/μL. In the induction period, 6.2% (4/65) of patients in the ozanimod 0.92 mg group and no patients in the other groups experienced ALC reductions <200 cells/μL. All patients interrupted treatment per protocol; 3 of these patients resumed ozanimod treatment after ALC levels increased to >500 cells/μL, and 1 patient who discontinued treatment had ALC levels increase to ≥500 cells/μL after treatment interruption. In the induction and maintenance periods, no patients in the placebo group, 3.0% (2/67) of patients in the ozanimod 0.46 mg group, and 13.8% (9/65) of patients in the ozanimod 0.92 mg group experienced ALC reductions <200 cells/μL. Of the 7 new patients who experienced an ALC reduction <200 cells/μL and interrupted study treatment during the maintenance period, 6 patients resumed treatment after ALC levels increased to >500 cells/μL, and ALC levels increased to ≥500 cells/μL after treatment interruption for the 1 patient who discontinued treatment.

In the induction and maintenance periods, no clinically significant abnormalities were noted in any treatment group for electrocardiograms (Tables A8, A9), vital signs (including heart rate, pulse rate, and blood pressure) (Table A10), or physical findings. No patients receiving ozanimod 0.46 mg or ozanimod 0.92 mg reported a mean supine and standing heart rate <50 bpm throughout the induction period and maintenance period.

## Discussion

This phase 2/3 study demonstrated that ozanimod was effective and well tolerated in Japanese patients with moderately to severely active UC. During the induction period, high efficacy rates were observed with ozanimod treatment, and significantly greater proportions of patients who received either ozanimod dose achieved the primary endpoint of clinical response versus placebo at week 12. Similar trends were observed for other endpoints, including clinical remission, endoscopic improvement, and mucosal healing. Notably, ozanimod 0.92 mg achieved higher efficacy rates for all evaluated endpoints than ozanimod 0.46 mg. Efficacy was well maintained during the study, with greater response rates in the ozanimod groups than in the placebo group.

As a phase 2/3 study, 2 ozanimod doses were assessed. Dose-dependent effects were observed for efficacy but not safety. Efficacy results in the maintenance period suggest that ozanimod 0.92 mg is the most effective dose to maintain remission. This is consistent with ozanimod labeling in the United States and other countries, including Japan, where ozanimod 0.92 mg is the recommended maintenance dose.^24^^,^^25^^,^^29^

The efficacy of ozanimod 0.92 mg in J-True North was consistent with that in the induction period of the global 52-week (10-week induction period and rerandomized 42-week maintenance period) True North study^22^; however, when comparing the trials qualitatively, efficacy outcomes were generally better in J-True North at week 12 than in True North at week 10.^22^ In the True North induction period, clinical response was achieved in 25.9% of patients receiving placebo and 47.8% of patients receiving ozanimod 0.92 mg, with a weighted difference of 21.9% compared with a placebo-adjusted difference of 30.1% in the ozanimod 0.92 mg group in J-True North. Similarly, clinical remission was achieved in 6.0% of patients receiving placebo and 18.4% of patients receiving ozanimod 0.92 mg with a weighted difference of 12.4% in True North compared with a placebo-adjusted difference of 22.4% in J-True North. Direct comparisons of efficacy results during the maintenance period by evaluating differences versus placebo are less meaningful due to the differences in study designs; in True North, clinical responders at week 10 were rerandomized to receive placebo or ozanimod, whereas clinical responders at week 12 continued the same treatment from induction during maintenance in J-True North. Placebo-adjusted differences cannot be compared, but the proportion of patients receiving ozanimod 0.92 mg during maintenance who achieved clinical response (60% [138/230] and 80% [32/40]) and clinical remission (37% [85/230] and 47.5% [19/40]) at week 52 in True North and J-True North, respectively, suggest that greater maintenance efficacy results were observed in J-True North. Differences in efficacy during induction may be highly attributed to the different time durations (12 weeks vs 10 weeks) between the induction periods of J-True North and True North, respectively. However, this may have a smaller influence on the greater outcomes observed in the maintenance period of J-True North. In addition, differences in patient populations may have led to better efficacy results in J-True North. Although there were no major differences in baseline characteristics, such as age or Mayo score, more patients were previously exposed to biologics in True North than in J-True North.

The efficacy of ozanimod 0.92 mg in J-True North was similar to that of the S1P modulator etrasimod in the phase 3 ELEVATE UC trials, with similar rates of placebo-adjusted treatment differences observed for clinical remission, clinical response, and endoscopic improvement at week 12; also, similar maintenance efficacy was observed at week 52 in the J-True North and ELEVATE UC 52 trials.^10^ However, head-to-head clinical trials comparing the efficacy of ozanimod versus etrasimod in patients with moderately to severely active UC are needed for direct treatment comparisons.

The safety profile of ozanimod in J-True North was consistent with previous ozanimod studies, with no unexpected safety signals.^20^^,^^22^^,^^31^^,^^32^ Certain S1P receptor modulators are associated with bradycardia, which may be due to S1P_1_ receptor binding in cardiac myocytes.^33^ There were no cases of bradycardia in J-True North, likely mitigated by the 7-day dose escalation upon dose initiation.^34^ There were no clinically significant electrocardiogram findings. There was 1 case of macular edema in the maintenance period. However, longer observation may be needed to accurately assess risk. In True North, macular edema occurred in 3 patients receiving ozanimod during the induction (cohort 1: 0.2% [1/429] and cohort 2: 0.3% [1/367]) and maintenance (0.4% [1/230]) periods and in 1 patient (0.8%; 0.2/100 patient-years) in the True North OLE.^22^^,^^35^ Herpes zoster infection occurred in 3 patients (2.3%) receiving ozanimod in J-True North, 8 patients (3 [0.4%] during induction and 5 [2.2%] during maintenance) in True North, and 7 patients (5.3%; 1.7 exposure-adjusted incidence rate per 100 patient-years) in the True North OLE. Unlike JAK inhibitors, which have previously demonstrated an increased risk of herpes zoster infection in Asians,^36^, ^37^, ^38^ our results demonstrate that Asian race did not impact rates of herpes zoster infection with ozanimod treatment in comparison to those observed in True North and its OLE.^22^^,^^35^ However, it is essential for future studies to collect real-world data on the safety of ozanimod in Asian patients with UC and to compare the findings with the safety of JAK inhibitors reported to date.

Infection and malignancies were not frequently observed with ozanimod even in the context of ALC reductions. This may be because ozanimod mainly reduces the distribution of naive CD4+ T cells and central memory subsets, but not effector CD8+ T cells, and has minimal impact on innate immune cells.^39^^,^^40^ Accordingly, neutrophil and leukocyte changes were limited in this study. No connection between ALC reduction and infection was observed in J-True North or True North. A post hoc analysis of the phase 3 RADIANCE and SUNBEAM MS trials demonstrated that IgG levels with ozanimod treatment were maintained within normal ranges despite dose-dependent decreases in circulating IgG levels, thus suggesting that ozanimod’s impact on lymphocytes does not greatly reduce circulating IgG levels.^41^ Similar trends regarding ALC reductions and infection were observed with ozanimod treatment in patients with MS. In a pooled safety analysis of all ozanimod MS studies, 1 (0.5%) patient had an ALC <0.2 × 10^9^/L around the onset of a serious infection, and 1 (0.5%) patient had a similar ALC level around the onset of a nonserious opportunistic infection.^42^ Notably, ALC reductions in patients with relapsing MS recovered to a normal range (≥1 × 10^9^/L) with a median time to recovery of 30 and 28 days after treatment discontinuation of ozanimod 0.92 mg and 0.46 mg, respectively, in a post hoc assessment of the off-treatment recovery of ALC.^43^

Although ALC reductions are a concern with ozanimod treatment, the severity of lymphopenia caused by ozanimod differs from NUDT15 gene variant thiopurine-induced leukopenia; ozanimod and thiopurine affect immune cells through different mechanisms, leading to varying degrees of immune modulation.^2^^,^^44^^,^^45^ ALC reduction by S1P receptor modulators is a consequence of limiting lymphocyte egression from secondary lymph organs, which preserves lymphocyte function,^19^^,^^46^^,^^47^ whereas leukopenia caused by thiopurines is a cytotoxic event with DNA damage resulting from irreversible bone marrow suppression.^48^ Preserved lymphocyte function with ozanimod treatment may partly explain the lack of association observed between the decrease of ALC and serious or opportunistic infections.

Findings from this study support the efficacy and safety of ozanimod in Japanese patients with moderately to severely active UC and its approval for the UC indication in Japan.^29^ Although Japanese inflammatory bowel disease treatment guidelines have not yet incorporated ozanimod positioning into the UC therapeutic armamentarium,^4^ the American Gastroenterological Association clinical practice guidelines for moderate to severe UC have suggested early use of advanced therapy, such as ozanimod, after failure of 5-aminosalicylic acid with or without immunomodulator therapy.^49^ This is consistent with the approved ozanimod UC indication in Japan for patients with moderately to severely active UC who have had an inadequate response to conventional therapies,^29^ thus providing Japanese clinicians with a better understanding of when to initiate ozanimod in clinical practice. This study is strengthened by its treat-through design, which allowed treatment groups to remain consistent for comparisons with placebo over 52 weeks. Although some Asian patients were included in the phase 3 True North study, this is the first analysis to verify the efficacy and safety of ozanimod in a large number of patients in Asia. A limitation of this trial is the paucity of long-term data, but the OLE phase of this study is ongoing. In addition, ozanimod has demonstrated long-term efficacy and safety for up to approximately 3 years of continuous ozanimod treatment in an interim analysis of the True North OLE.^35^ Another limitation of this study is that corticosteroid-free remission results could not be interpreted due to the small number of patients receiving concomitant corticosteroid (placebo, n = 3; ozanimod 0.46 mg, n = 4; ozanimod 0.92 mg, n = 7).

## Conclusions

Ozanimod was effective and well tolerated as a once-daily oral therapy in Japanese patients with moderately to severely active UC. Results of this large-scale Japanese clinical trial verified the efficacy and safety of ozanimod in a large number of patients in Asia for the first time. The efficacy and safety profile of ozanimod in J-True North was consistent with the findings of the global phase 3 True North study.

## Supplementary data

Appendix

Extended PDF
