# Supplementary material for: Once-Daily Oral Ozanimod for Japanese Patients With Ulcerative Colitis: Results From the Phase 2/3 J-True North Study
Source: Gastro Hep Adv. 2025 Sep 16;5(1):100812. doi: 10.1016/j.gastha.2025.100812 (PMC12630022; doi:10.1016/j.gastha.2025.100812)
Supplement: Appendix [file mmc1.pdf]

SUPPLEMENTARY MATERIAL

Supplementary Methods

Inclusion Criteria

1. Japanese adults aged 18 to 75 years at the time of signing the informed consent form at screening.
2. Patient has had ulcerative colitis (UC) diagnosed ≥3 months prior to first investigational product administration. The diagnosis should be confirmed by clinical and endoscopic evidence and corroborated by a histopathology report (endoscopy and histopathology may be performed at screening if no prior report is readily available).
3. Patient has evidence of UC extending ≥15 cm from the anal verge as determined by baseline endoscopy (flexible sigmoidoscopy or colonoscopy).
4. Patient has active UC defined as Mayo score of 6–12 inclusive, with Mayo endoscopy subscore of ≥2, a rectal bleeding subscore of ≥1, and a stool frequency subscore ≥1.
5. Patient must have been treated with aminosalicylates or corticosteroids. If patients are currently receiving treatment with ≥1 of the following therapies, the patient must continue on these therapies during Induction:
   a. Oral aminosalicylates at a therapeutic dose for their disease (eg, mesalazine, sulfasalazine), with the dose stable for ≥2 weeks prior to screening endoscopy.
   b. Prednisolone (doses ≤10 mg/day) or equivalent receiving a stable dose for ≥2 weeks prior to screening endoscopy.
6. Patient has undergone colonoscopy (or is willing to undergo colonoscopy during screening):
   a. Within the past 2 years, to screen for dysplasia (unless otherwise recommended by local and national guidelines) if the patient has had left-sided colitis for >12 years or total/extensive colitis for >8 years.
   b. Within the past 5 years, to screen for polyps if the patient age is >45 years.

7. If oral aminosalicylates or corticosteroids have been recently discontinued, patient must have been stopped for ≥2 weeks prior to the endoscopy used for baseline Mayo score.
8. Females of childbearing potential:
    a. Patient must agree to practice a highly effective method of contraception throughout the study until completion of the 90-day safety follow-up visit. Highly effective methods of contraception are those that alone or in combination result in a failure rate of a Pearl Index of <1% per year when used consistently and correctly. The following are acceptable methods of birth control in the study:
        i. Combined hormonal (containing estrogen and progestogen) contraception, which may be oral, intravaginal, or transdermal
        ii. Progestogen-only hormonal contraception associated with inhibition of ovulation, which may be oral, injectable, or implantable
        iii. Placement of an intrauterine device
        iv. Placement of a hormone-releasing intrauterine system
        v. Bilateral tubal occlusion
        vi. Vasectomized partner
        vii. Complete sexual abstinence
    b. A female of childbearing potential is a sexually mature female who (1) has not undergone a hysterectomy (the surgical removal of the uterus) or bilateral oophorectomy (the surgical removal of both ovaries) or (2) has not been postmenopausal for ≥24 consecutive months (ie, has had menses at any time during the preceding 24 consecutive months). Periodic abstinence (calendar, symptothermal, postovulation methods), withdrawal (coitus interruptus), spermicides only, and the lactational amenorrhea method are not acceptable methods of contraception.
9. Patient provides written informed consent and confirms compliance with the schedule of protocol assessments.

10. Patients must have documentation of positive varicella zoster virus immunoglobulin G antibody status or complete varicella zoster virus vaccination ≥30 days prior to randomization.

Exclusion Criteria

Exclusions Related to General Health

1. Patient has severe extensive colitis as evidenced by:
    a. Physician judgment that the patient is likely to require colectomy or ileostomy within 12 weeks of baseline.
    b. Current or recent (within 3 months of screening) evidence of fulminant colitis, toxic megacolon, or bowel perforation.
2. Patient has a diagnosis of Crohn's disease, indeterminate colitis, the presence or history of a fistula consistent with Crohn's disease, microscopic colitis, radiation colitis, or ischemic colitis.
3. Patient has a positive stool examination for pathogens (ova and parasites, bacteria) or a positive test for toxin producing Clostridioides difficile (C. difficile) at screening. Polymerase chain reaction examination of the stool for C. difficile may be used to exclude false positives. If positive, patients may be treated and retested. Documentation of a negative test result for pathogens (ova and parasites, bacteria) is required within 60 days of day 1.
4. Patient is pregnant or breastfeeding, or has a positive serum beta-human chorionic gonadotropin measured during screening.
5. Patient has clinically relevant hepatic, neurological, pulmonary (severe respiratory disease, pulmonary fibrosis and chronic obstructive pulmonary disease), ophthalmological, endocrine, psychiatric, or other major systemic disease making implementation of the protocol or interpretation of the study difficult or that would put the patient at risk by participating in the study.
6. Patient has clinically relevant cardiovascular conditions, including history or presence of:
    a. Recent (within the last 6 months of screening) occurrence of myocardial infarction, unstable angina, stroke, transient ischemic attack, symptomatic

bradycardia, decompensated heart failure requiring hospitalization, class III/IV heart failure, or severe untreated sleep apnea.

b. Second degree (Mobitz type II) atrioventricular block, third-degree atrioventricular block, sick sinus syndrome, or sinoatrial block in patients without a pacemaker in place; if second-degree Type II or third-degree atrioventricular block is due to concomitant medication, consult the medical monitor prior to screening.

c. Prolonged QT interval corrected for heart rate using Fridericia's formula (QTcF; QTcF >450 msec males, >470 msec females) at either screening or day 1 predose assessment. One recheck is allowed for patients per visit (ie, during the screening and/or day 1 predose assessment visit).

d. Resting heart rate <55 beats per minute when taking vital signs as part of the physical examination at either screening or day 1 predose assessment. One recheck is allowed for patients with heart rate <55 beats per minute per visit (ie, during the screening and/or day 1 predose assessment visit).

e. Patients with the preexisting cardiac conditions listed below must be seen by a consulting cardiologist and cleared to participate in the study without more intensive monitoring. These patients will also follow first-dose monitoring procedures.

    i. History (>6 months prior to screening) of ischemic heart disease, cardiac arrest, cerebrovascular disease, uncontrolled hypertension, history of recurrent syncope, or symptomatic bradycardia

    ii. Second-degree (Mobitz type II) atrioventricular block, third-degree atrioventricular block, sick sinus syndrome, or sinoatrial block with functional pacemaker

    iii. Patients on medicinal products that may potentiate bradycardia (other than the combination of beta-blockers and calcium channel blockers)

7. Patient has a history of diabetes mellitus type 1 or uncontrolled diabetes mellitus type 2 with glycosylated hemoglobin >9%; or diabetic patients with significant

comorbid conditions (eg, retinopathy, nephropathy).

8. Patient has a history of uveitis (within the last year) or a history of macular edema.
9. Patient has a known active bacterial, viral, or fungal infection (excluding fungal infection of nail beds, minor upper respiratory tract infections, and minor skin infections), a mycobacterial infection (including tuberculosis or atypical mycobacterial disease), or any major episode of infection that either required hospitalization or treatment with intravenous antibiotics within 30 days of screening, or treatment with oral antibiotics within 14 days of screening.
    a. In the case of a known SARS-CoV-2 infection, symptoms must have completely resolved; based on investigator assessment in consultation with the clinical trial physician/medical monitor, there are no sequelae that would place the patient at a higher risk of receiving investigational treatment.
10. Patient has a history or known presence of recurrent or chronic infection (eg, hepatitis A, B, or C; HIV); recurrent urinary tract infections are allowed.
11. Patient has a history of cancer, including solid tumors and hematological malignancies (except basal cell and in situ squamous cell carcinomas of the skin or uterine cervix that have been excised and resolved), or colonic mucosal dysplasia.
12. Patient has a history of alcohol or drug abuse within 1 year prior to randomization.
13. Patient has a history of or currently active primary or secondary immunodeficiency.

Exclusions Related to Medications

1. Patient has a history of treatment with a biologic agent within 8 weeks or 5 elimination half-lives (whichever is less) of that agent prior to randomization.
2. Patient has a history of treatment with tofacitinib within 5 elimination half-lives of that agent prior to randomization.
3. Patient has a history of treatment with an investigational agent within 5 elimination half-lives of that agent prior to randomization.

4. Patient has a history of treatment with topical rectal 5-aminosalicylic acid or topical rectal steroids within 2 weeks of screening endoscopy or antimotility medications (such as diphenoxylate/atropine) during screening.
5. Patient has received a live vaccine or live attenuated vaccine within 4 weeks prior to randomization.
6. Patient has been treated previously with lymphocyte-depleting therapies (eg, alemtuzumab, anti-CD4, cladribine, rituximab, cyclophosphamide, mitoxantrone, total body irradiation, bone marrow transplantation).
7. Patient has been treated with cyclosporine, tacrolimus, sirolimus, or mycophenolate mofetil within 16 weeks of screening. If no renal disorder or cutaneous malignancy is confirmed, the washout period of tacrolimus can be reduced to 8 weeks. To check renal disorder, serum creatinine/glomerular filtration rate, albumin-to-creatinine ratio, and serum potassium should be measured.
8. Patient has been treated previously with D-penicillamine, leflunomide, or thalidomide.
9. Patient has been treated previously with natalizumab, fingolimod, or other sphingosine 1-phosphate receptor modulators.
10. Patient has a history of treatment with intravenous immunoglobulin or plasmapheresis within 3 months prior to randomization.
11. Patient has planned concurrent treatment with antineoplastic immunosuppressive agents (ie, azathioprine or methotrexate) after randomization. Patients receiving azathioprine or methotrexate at screening must discontinue treatment with these agents 4 weeks prior to randomization.
12. Patient is treated with chronic nonsteroidal antiinflammatory drugs (occasional use of nonsteroidal antiinflammatory drugs and acetaminophen [for headache, arthritis, myalgias, or menstrual cramps] and aspirin up to 325 mg/day is permitted).
13. Patient is treated with class Ia or class III antiarrhythmic drugs or with ≥2 agents in a combination known to prolong PR interval, or treatment with additional prohibited systemic cardiac medications.

14. Patient has been treated with apheresis within 2 weeks of randomization.
15. Patients who were primary nonresponders to ≥2 biologic agents approved for the treatment of UC (ie, anti–tumor necrosis factor agents or vedolizumab).
16. Patient is receiving treatment with breast cancer resistance protein inhibitors (eg, cyclosporine, eltrombopag).
17. Patient is receiving treatment with any of the following drugs or interventions within the corresponding timeframe:
    a. At randomization
        i. CYP2C8 inhibitors (eg, clopidogrel) or inducers (eg, rifampicin)
    b. 2 weeks prior to randomization
        i. Monoamine oxidase inhibitors (eg, selegiline).

Exclusions Related to Laboratory Results and Other Assessments

18. Patient had the following laboratory results:
    a. Serum creatinine >1.4 mg/dL for females or >1.6 mg/dL for males
    b. Liver function impairment or persisting elevations of aspartate aminotransferase or alanine aminotransferase >2 × the upper limit of normal (ULN) or direct bilirubin >1.5 × ULN
    c. Platelet count <100,000/µL
    d. Hemoglobin <8.0 g/dL
    e. Neutrophils <1500 /µL
    f. Absolute white blood cell count <3500/µL
    g. Absolute lymphocyte count <800/µL
    h. ECG showing any clinically significant abnormality

Study Design

Open-label extension (OLE)

Patients who experienced disease relapse during the maintenance period were eligible to enter the OLE if they met all of the following criteria: increase in UC disease activity as defined by an increase in partial Mayo score of ≥2 points compared with the week 12 partial Mayo score with an absolute partial Mayo score ≥4 points, Mayo endoscopy subscore ≥2 points, and exclusion of other causes of an increase in disease activity

unrelated to underlying UC (eg, infections, change in medication). Patients withdrew from the OLE if they did not achieve clinical improvement after 12 weeks of ozanimod treatment in the OLE. The OLE will continue until marketing launch or until the sponsor discontinues the development program.

Supplementary Table 1. List of Japan-True North Study Principal Investigators, Sites, and Institutional or Ethical Review Boards

Site No.	Principal investigator name	Name/address of EIC/IRB
101	Kawaratani, Hideto	Nara Medical University Hospital
102	Matsuoka, Katsuyoshi	Toho University Medical Center Sakura Hospital
103	Motoya, Satoshi	Sapporo-Kosei General Hospital
104	Ishigami, Keisuke	Sapporo Medical University Hospital
105	Watanabe, Kenji	Hyogo College of Medicine Hospital
106	Oka, Shiro	Hiroshima University Hospital
107	Nakano, Masaru	Kitasato University Kitasato Institute Hospital
108	Saruta, Masayuki	Jikei University Hospital
109	Hisamatsu, Tadakazu	Kyorin University Hospital
110	Matsumoto, Takayuki	Iwate Medical University Uchimaru Medical Center
111	Takedatsu, Hidetoshi	Kurume University Hospital
112	Inaba, Tomoki	Kagawa Prefectural Central Hospital
113	Bamba, Shigeki	Shiga University of Medical Science Hospital
114	Hasatani, Kenkei	Fukui Prefectural Hospital
115	Kimura, Tsuguhiro	Medical Corporation Shoyu-Kai Fujita Gastroenterology Hospital
116	Tokito, Satoki	Tokitokai Tokito Clinic
117	Fukata, Masayuki	Tokyo Yamate Medical Center
118	Ninomiya, Tomoyuki	Ehime Prefectural Central Hospital
119	Hisanaga, Yasuhiro	Ogaki Municipal Hospital
120	Ito, Toru	Kanazawa Medical University Hospital
121	Matano, Yutaka	Komatsu Municipal Hospital
122	Hisabe, Takashi	Fukuoka University Chikushi Hospital
123	Horiki, Noriyuki	Mie University hospital
124	Munemoto, Yoshinori	Fukui-ken Saiseikai Hospital
125	Takamura, Masaaki	Nagaoka Chuo General Hospital
126	Kamoshida, Toshiro	Hitachi General Hospital
127	Sato, Yuichiro	Osaki Citizen Hospital
128	Minato, Yohei	NTT Medical Center Tokyo
129	Kamiyamamoto, Shinji	Toyama City Hospital
130	Kodama, Yuzo	Kobe University Hospital
131	Imaeda, Hiroyuki	Saitama Medical University Hospital
132	Hosomi, Shuhei	Osaka Metropolitan University
133	Sakuraba, Hirotake	Hirosaki University Hospital
134	Matsushima, Masashi	Tokai University Hospital
135	Nakatsu, Morihito	Mitoyo General Hospital
136	Yoshioka, Masao	Okayama Saiseikai Outpatient Center Hospital
137	Tanaka, Toshio	Shizuoka City Shizuoka Hospital
138	Fujii, Toshimitsu	Institute of Science Tokyo
139	Arai, Takehiro	Tokatsu Tsujinaka Hospital
140	Yamazaki, Kenji	Gifu Prefectural General Medical Center
141	Naito, Yuji	University Hospital Kyoto Prefectural University of Medicine
142	Kawano, Hiroshi	Our Lady of the Snow Social Medical Corporation St. Mary's Hospital
143	Kanda, Naoki	Takatsuki Red Cross Hospital
144	Ishino, Atsushi	Hoshi General Hospital
145	Hiramatsu, Naoki	Osaka Rosai Hospital

146	Kumagai, Shinji	IMS Meirikai Sendai General Hospital
147	Furuya, Ken	Japan Community Health Care Organization Hokkaido Hospital
148	Kanke, Kazunari	Kanke Gastrointestinal Clinic
149	Ohnishi, Yoshifumi	National Hospital Organization Shizuoka Medical Center
150	Sai, Souken	Sai Gastroenterology and Proctology Clinic
151	Kobayashi, Toshihisa	Hakodate Goryoukaku Hospital
152	Kuroda, Tsuyoshi	Mazda Hospital of Mazda Motor Corporation
153	Fukuchi, Takumi	Iseikai Hospital
154	Sakata, Yasuhisa	Saga University Hospital
155	Aoyagi, Kunihiko	Japanese Red Cross Fukuoka Hospital
156	Hiraoka, Sakiko	Okayama University Hospital
157	Kato, Shingo	Saitama Medical Center, Saitama Medical University
158	Takeuchi, Ken	Tsujinaka Hospital Kashiwanoha
159	Ishida, Tetsuya	Ishida Clinic of IBD and Gastroenterology
160	Watanabe, Chiyuki	Hiroshima Prefectural Hospital
161	Ochiai, Toshiaki	Saiseikai Fukuoka General Hospital
162	Kubokawa, Masaru	Aso Iizuka Hospital
163	Aoyama, Nobuo	Aoyama Clinic GI Endoscopy and IBD Center
164	Haraguchi, Kazuhiro	Hara Sanshin Hospital
165	Hidaka, Hisamitsu	Hidaka Coloproctology Clinic
166	Tobita, Kouji	Hiratsuka Gastroenterological Hospital
167	Yamamura, Takeshi	Nagoya University Hospital

EIC, ethical review board; IRB, institutional review board.

Supplementary Table 2. Definitions of Efficacy Endpoints

Efficacy endpoints	Definition
Clinical response	• Complete Mayo score definition: a reduction from baseline in the complete Mayo score of ≥3 points and ≥30% and a reduction from baseline in the RBS of ≥1 point or an absolute RBS of ≤1 point<br>− Complete Mayo score: sum of SFS, RBS, Mayo endoscopy subscore, Physician Global Assessment (each assessment rated from 0–3)<br>• 9-point Mayo score definition: reduction from baseline in the 9-point Mayo score of ≥2 points and ≥35%, and a reduction from baseline in the RBS of ≥1 point or an absolute RBS of ≤1 point<br>− 9-point Mayo score: sum of RBS, SFS, and Mayo endoscopy subscore (each assessment rated from 0–3)
Clinical remission	• Definition 1: complete Mayo score of ≤2 points with no individual subscore ≥1 point<br>• Definition 2: RBS = 0 and SFS ≤1 (and a decrease of ≥1 point from baseline SFS) and Mayo endoscopy subscore ≤1<br>• Definition 3: SFS = 0 or 1 (without a requirement of a decrease of ≥1 from baseline SFS), RBS = 0, endoscopy subscore = 0 or 1

Endoscopic improvement	• Endoscopy subscore ≤1
Mucosal healing	• Endoscopy subscore ≤1 with a Geboes score <2.0
Histologic remission	• Geboes score <2.0

RBS, rectal bleeding subscore; SFS, stool frequency subscore.

Supplementary Table 3. Change From Baseline in FCP and CRP Levels at Weeks 12 and 52

	Induction (Week 12)			Maintenance (Week 52)		
	**Placebo**	**Ozanimod 0.46 mg**	**Ozanimod 0.92 mg**	**Placebo**	**Ozanimod 0.46 mg**	**Ozanimod 0.92 mg**
**FCP, µg/g**						
n	65	68	65	65	68	65
Mean (SD) at baseline	2168.6 (3173.2)	2649.0 (3532.7)	2784.4 (4418.4)	2168.6 (3173.2)	2649.0 (3532.7)	2784.4 (4418.4)
Median (range) at baseline	1060.0 (12.7, 16,800.0)	1500.0 (26.6, 15,200.0)	885.0 (19.6, 22,200.0)	1060.0 (12.7, 16,800.0)	1500.0 (26.6, 15,200.0)	885.0 (19.6, 22,200.0)
n	59	59	59	12	34	35
Mean (SD) change from baseline	−230.1 (4248.0)	−1693.2 (3674.3)	−1581.0 (5379.3)	−1445.4 (1504.7)	−2015.9 (3426.2)	−2449.1 (6344.0)
Median (range) change from baseline	−28.4 (−16,781.7, 10,340.0)	−567.0 (−15,190.0, 7671.5)	−494.2 (−21,340.0, 17,100.0)	−986.0 (−3961.0, 495.3)	−1168.5 (−15,190.0, 6963.0)	−638.3 (−22,185.2, 17,608.0)
**CRP, mg/L**						
n	65	68	65	65	68	65
Mean (SD) at baseline	3.7 (7.6)	4.5 (8.4)	3.5 (6.4)	3.7 (7.6)	4.5 (8.4)	3.5 (6.4)
Median (range) at baseline	1.4 (0.1, 49.7)	1.7 (0.1, 54.2)	1.6 (0.1, 42.7)	1.4 (0.1, 49.7)	1.7 (0.1, 54.2)	1.6 (0.1, 42.7)
n	59	59	59	12	34	35
Mean (SD) change from baseline	−0.5 (5.5)	−2.3 (7.9)	−1.2 (7.2)	−0.4 (6.1)	−1.4 (5.8)	−1.5 (3.6)
Median (range) change from baseline	−0.0 (−25.5, 15.6)	−0.7 (−52.3, 2.3)	−0.2 (−30.8, 35.1)	−0.6 (−8.7, 17.0)	−0.6 (−28.4, 9.5)	−0.5 (−14.7, 4.4)

CRP, C-reactive protein; FCP, fecal calprotectin; SD, standard deviation.

Supplementary Table 4. FCP Response at Week 12

FCP, μg/g	Placebo (N=65)	Ozanimod 0.46 mg (N=68)	Ozanimod 0.92 mg (N=65)
>50 to ≤50 μg/g			
Number of patients with change in FCP levels from baseline, n/N (%)	3/55 (5.5)	19/57 (33.3)	13/57 (22.8)
>100 to ≤100 μg/g			
Number of patients with change in FCP levels from baseline, n/N (%)	7/49 (14.3)	24/56 (42.9)	18/53 (34.0)
>150 to ≤150 μg/g			
Number of patients with change in FCP levels from baseline, n/N (%)	9/47 (19.1)	24/52 (46.2)	20/52 (38.5)

FCP, fecal calprotectin.

Supplementary Table 5. Change From Baseline in Leukocyte and Neutrophil Counts at Weeks 12 and 52

	Induction (Week 12)			Maintenance (Week 52)		
	**Placebo**	**Ozanimod 0.46 mg**	**Ozanimod 0.92 mg**	**Placebo**	**Ozanimod 0.46 mg**	**Ozanimod 0.92 mg**
**Leukocytes, 10^9/L**						
n	65	68	65	65	68	65
Mean (SD) at baseline	6.5 (1.8)	6.7 (2.3)	6.7 (2.1)	6.5 (1.8)	6.7 (2.3)	6.7 (2.1)
n	58	59	59	12	34	35
Mean (SD) change from baseline	0.9 (2.4)	−1.8 (2.0)	−2.1 (1.7)	−0.2 (1.3)	−1.9 (2.1)	−2.0 (1.6)
**Neutrophils, 10^9/L**						
n	65	68	65	65	68	65
Mean (SD) at baseline	4.2 (1.5)	4.4 (2.0)	4.5 (2.0)	4.2 (1.5)	4.4 (2.0)	4.5 (2.0)
n	58	59	59	12	34	35
Mean (SD) change from baseline	1.1 (2.3)	−0.7 (1.8)	−0.9 (1.5)	−0.3 (1.3)	−0.6 (2.0)	−0.9 (1.5)

SD, standard deviation.

Supplementary Table 6. Patients With Abnormal Neutrophil Count or Leukocyte Count

	Induction (Week 12)			Maintenance (Week 52)		
	**Placebo**	**Ozanimod 0.46 mg**	**Ozanimod 0.92 mg**	**Placebo**	**Ozanimod 0.46 mg**	**Ozanimod 0.92 mg**
Baseline[a]						
n	65	68	65	65	68	65
ANC <1000 cells/mL	0	1 (1.5)	1 (1.5)	0	1 (1.5)	1 (1.5)
Total WBC >20,000 cells/μL	0	0	0	0	0	0
Overall[b]						
n	65	67	65	65	67	65
ANC <1000 cells/mL	0	1 (1.5)	0	0	1 (1.5)	0
Total WBC >20,000 cells/μL	1 (1.5)	0	0	1 (1.5)	0	0

[a]Baseline is defined as the last nonmissing record on or before the first dose of study drug.

[b]Patients' postbaseline assessments are used to derive abnormality.

ANC, absolute neutrophil count; WBC, white blood cell.

Supplementary Table 7. TEAEs With Incidence >2% (safety population)

	IP			IP and MP		
	**Placebo (n=65)**	**Ozanimod 0.46 mg (n=68)**	**Ozanimod 0.92 mg (n=65)**	**Placebo (n=65)**	**Ozanimod 0.46 mg (n=68)**	**Ozanimod 0.92 mg (n=65)**
TEAE with incidence ≥2%						
Nasopharyngitis	4 (6.2)	5 (7.4)	5 (7.7)	6 (9.2)	10 (14.7)	9 (13.8)
Pyrexia	2 (3.1)	6 (8.8)	2 (3.1)	3 (4.6)	11 (16.2)	7 (10.8)
Headache	4 (6.2)	4 (5.9)	4 (6.2)	4 (6.2)	8 (11.8)	6 (9.2)
Back pain	5 (7.7)	2 (2.9)	3 (4.6)	5 (7.7)	5 (7.4)	6 (9.2)
COVID-19	2 (3.1)	2 (2.9)	1 (1.5)	3 (4.6)	4 (5.9)	5 (7.7)
Colitis ulcerative	1 (1.5)	4 (5.9)	4 (6.2)	1 (1.5)	4 (5.9)	4 (6.2)
Arthralgia	1 (1.5)	2 (2.9)	3 (4.6)	2 (3.1)	4 (5.9)	4 (6.2)
Abdominal pain	1 (1.5)	2 (2.9)	0	1 (1.5)	4 (5.9)	2 (3.1)
GGT increased	0	1 (1.5)	1 (1.5)	0	5 (7.4)	2 (3.1)
Dental caries	1 (1.5)	3 (4.4)	0	2 (3.1)	5 (7.4)	0
Nausea	1 (1.5)	1 (1.5)	1 (1.5)	1 (1.5)	2 (2.9)	3 (4.6)
ALT increased	0	1 (1.5)	2 (3.1)	1 (1.5)	1 (1.5)	3 (4.6)
Constipation	0	1 (1.5)	1 (1.5)	0	1 (1.5)	3 (4.6)
Insomnia	1 (1.5)	1 (1.5)	1 (1.5)	1 (1.5)	1 (1.5)	3 (4.6)
Malaise	1 (1.5)	1 (1.5)	2 (3.1)	1 (1.5)	1 (1.5)	3 (4.6)
Hepatic function abnormal	1 (1.5)	0	2 (3.1)	1 (1.5)	0	3 (4.6)
Vertigo	0	0	1 (1.5)	0	0	3 (4.6)
Periodontal disease	0	0	1 (1.5)	0	2 (2.9)	2 (3.1)
Rash	1 (1.5)	2 (2.9)	1 (1.5)	1 (1.5)	2 (2.9)	2 (3.1)
Wound	0	0	2 (3.1)	0	2 (2.9)	2 (3.1)
AST increased	0	0	2 (3.1)	0	1 (1.5)	2 (3.1)
Contusion	0	0	1 (1.5)	0	1 (1.5)	2 (3.1)
Diarrhea	0	0	1 (1.5)	0	1 (1.5)	2 (3.1)
Herpes zoster	1 (1.5)	0	1 (1.5)	1 (1.5)	1 (1.5)	2 (3.1)
Liver function test increased	0	2 (2.9)	1 (1.5)	0	2 (2.9)	2 (3.1)
SARS-CoV-2 test positive	0	0	0	1 (1.5)	1 (1.5)	2 (3.1)
Cough	0	0	1 (1.5)	0	0	2 (3.1)
Dizziness	1 (1.5)	0	0	2 (3.1)	0	2 (3.1)
Ocular hypertension	0	0	1 (1.5)	0	0	2 (3.1)
Peripheral edema	2 (3.1)	0	1 (1.5)	3 (4.6)	0	2 (3.1)
Oropharyngeal discomfort	0	0	2 (3.1)	0	0	2 (3.1)
Orthostatic hypotension	0	0	0	0	0	2 (3.1)
Abdominal pain upper	1 (1.5)	2 (2.9)	1 (1.5)	1 (1.5)	2 (2.9)	1 (1.5)
Oropharyngeal pain	1 (1.5)	2 (2.9)	0	1 (1.5)	3 (4.4)	1 (1.5)
Oral herpes	0	0	0	0	2 (2.9)	1 (1.5)

Stomatitis	0	2 (2.9)	0	0	3 (4.4)	0
Animal bite	0	1 (1.5)	0	0	2 (2.9)	0
Cystitis	0	0	0	0	2 (2.9)	0
Dyspepsia	0	2 (2.9)	0	0	2 (2.9)	0
Epistaxis	0	2 (2.9)	0	0	2 (2.9)	0
Gastroenteritis	0	0	0	2 (3.1)	2 (2.9)	0
Hepatic enzyme increased	0	2 (2.9)	0	0	2 (2.9)	0
Hypertension	0	2 (2.9)	0	0	2 (2.9)	0
Large intestine polyp	0	1 (1.5)	0	0	2 (2.9)	0
Nasal vestibulitis	0	2 (2.9)	0	0	2 (2.9)	0
Noncardiac chest pain	0	1 (1.5)	0	0	2 (2.9)	0
Pain in extremity	1 (1.5)	1 (1.5)	0	1 (1.5)	2 (2.9)	0
Hordeolum	1 (1.5)	0	0	2 (3.1)	1 (1.5)	0
Immunization reaction	3 (4.6)	1 (1.5)	0	3 (4.6)	1 (1.5)	0

ALT, alanine aminotransferase; AST, aspartate aminotransferase; GGT, gamma-glutamyl transferase; IP, induction period; MP, maintenance period; TEAE, treatment-emergent adverse event.

Supplementary Table 8. Abnormal Electrocardiogram Values at Hour 6 in Patients With 6-Hour Cardiac Monitoring on Day 1 (induction period, safety population)

	**Placebo (N=65)**	**Ozanimod 0.46 mg (N=68)**	**Ozanimod 0.92 mg (N=65)**
n	24	21	22
QT >480 ms	0	0	0
QT >500 ms	0	0	0
QTcF >480 ms	0	1 (4.8)	0
QTcF >500 ms	0	1 (4.8)	0
Change from baseline in QT of >30 ms	1 (4.2)	4 (19.0)	7 (31.8)
Change from baseline in QT of >60 ms	0	1 (4.8)	0
Change from baseline in QTcF of >30 ms	0	1 (4.8)	1 (4.5)
Change from baseline in QTcF of >60 ms	0	1 (4.8)	0

Data are n (%).

QTcF, QT interval corrected for heart rate using Fridericia’s formula.

Supplementary Table 9. Electrocardiogram Findings in the Induction and Maintenance Periods (safety population)

	**IP**			**IP and MP**		
	**Placebo (N=65)**	**Ozanimod 0.46 mg (N=68)**	**Ozanimod 0.92 mg (N=65)**	**Placebo (N=65)**	**Ozanimod 0.46 mg (N=68)**	**Ozanimod 0.92 mg (N=645)**
n	65	67	64	65	67	64
QT >480 ms	0	0	0	0	1 (1.5)	0
QT >500 ms	0	0	0	0	1 (1.5)	0
QTcF >480 ms	0	1 (1.5)	0	0	2 (3.0)	0
QTcF >500 ms	0	1 (1.5)	0	0	2 (3.0)	0
Change from baseline in QT of >30 ms	6 (9.2)	15 (22.4)	14 (21.9)	6 (9.2)	18 (26.9)	17 (26.6)
Change from baseline in QT of >60 ms	0	2 (3.0)	0	0	4 (6.0)	0
Change from baseline in QTcF of >30 ms	0	3 (4.5)	3 (4.7)	0	5 (7.5)	4 (6.3)
Change from baseline in QTcF of >60 ms	0	1 (1.5)	0	0	2 (3.0)	0

Data are n (%).

IP, induction period; MP, maintenance period; QTcF, QT interval corrected for heart rate using Fridericia's formula.

Supplementary Table 10. Mean (standard deviation) Heart Rate and Change From Baseline in Heart Rate During Cardiac Monitoring on Study Day 1 Hours 1–6 (induction period, safety population)

	**Placebo (N=65)**	**Ozanimod 0.46 mg (N=68)**	**Ozanimod 0.92 mg (N=65)**
Pulse rate, supine (bpm), n	24	21	22
Mean (SD) HR			
Baseline	68.5 (10.0)	71.3 (10.3)	69.1 (8.8)
Hour 1	66.7 (9.1)	69.3 (9.9)	73.0 (9.2)
Hour 2	69.4 (9.5)	72.3 (10.4)	70.1 (8.9)
Hour 3	70.0 (9.5)	72.2 (9.4)	67.7 (10.3)
Hour 4	68.5 (7.6)	70.1 (9.0)	70.2 (8.0)
Hour 5	66.9 (7.1)	69.3 (9.6)	68.2 (9.5)
Hour 6	67.8 (9.9)	68.3 (8.6)	67.2 (8.2)
Mean (SD) change from baseline in HR			
Hour 1	−0.5 (5.6)	−2.2 (7.5)	0.4 (8.5)
Hour 2	2.2 (7.9)	0.7 (8.6)	−2.4 (7.3)
Hour 3	2.8 (8.5)	0.7 (8.4)	−4.8 (7.3)
Hour 4	1.3 (7.4)	−1.5 (9.7)	−2.4 (8.3)
Hour 5	−0.3 (6.2)	−2.3 (9.7)	−4.3 (7.7)
Hour 6	0.6 (8.0)	−3.3 (9.1)	−5.3 (7.8)
Pulse rate, standing (bpm), n	24	21	22
Mean (SD) HR			
Baseline	81.0 (14.0)	85.0 (12.2)	82.8 (11.3)
Hour 1	78.6 (11.6)	82.5 (12.4)	84.5 (9.5)
Hour 2	84.4 (12.5)	85.8 (14.7)	80.2 (9.7)
Hour 3	87.7 (13.1)	83.7 (13.1)	80.4 (8.7)
Hour 4	85.2 (9.4)	82.2 (10.1)	80.8 (9.6)
Hour 5	80.4 (9.6)	80.2 (9.7)	79.6 (11.3)
Hour 6	79.3 (9.7)	80.1 (9.3)	76.0 (9.4)
Mean (SD) change from baseline in HR			
Hour 1	−1.3 (7.8)	−1.4 (9.3)	1.9 (9.8)
Hour 2	4.5 (10.7)	1.9 (11.6)	−2.4 (8.7)
Hour 3	7.9 (11.8)	−0.2 (10.4)	−2.2 (8.5)
Hour 4	5.3 (10.2)	−1.7 (10.9)	−1.8 (9.6)
Hour 5	0.5 (10.3)	−3.7 (9.8)	−2.9 (12.0)
Hour 6	−0.6 (8.7)	−3.8 (8.6)	−6.5 (10.6)

bpm, beats per minute; HR, heart rate; SD, standard deviation.

Supplementary Figure 1. Study design of the phase 3 Japan-True North study.

[a]The maintenance period was initially planned as 52 weeks but was shortened to 40 weeks in an amendment to the protocol. This did not affect the timing of efficacy endpoints at week 52 for the maintenance period. [b]Patients were stratified by corticosteroid use at screening (yes or no) and prior biologic use (yes or no). [c]Ozanimod was initiated at a dose of 0.23 mg for 4 days, then ozanimod 0.46 mg for 3 days, followed thereafter by the assigned treatment level (ie, ozanimod 0.46 mg or ozanimod 0.92 mg). [d]Patients who did not achieve clinical response (defined as a reduction from baseline in the complete Mayo score ≥3 points and ≥30% and a reduction from baseline in the RBS of ≥1 point or an absolute RBS of ≤1 point) at week 12. [e]Disease relapse: increase in UC disease activity as defined by an increase in partial Mayo score ≥2 points compared with the week 12 partial Mayo score with an absolute partial Mayo score ≥4 points, an endoscopic subscore of ≥2 points, and exclusion of other causes of an increase in disease activity unrelated to underlying UC (eg, infections, change in medication). OLE, open-label extension; RBS, rectal bleeding subscore; UC, ulcerative colitis.

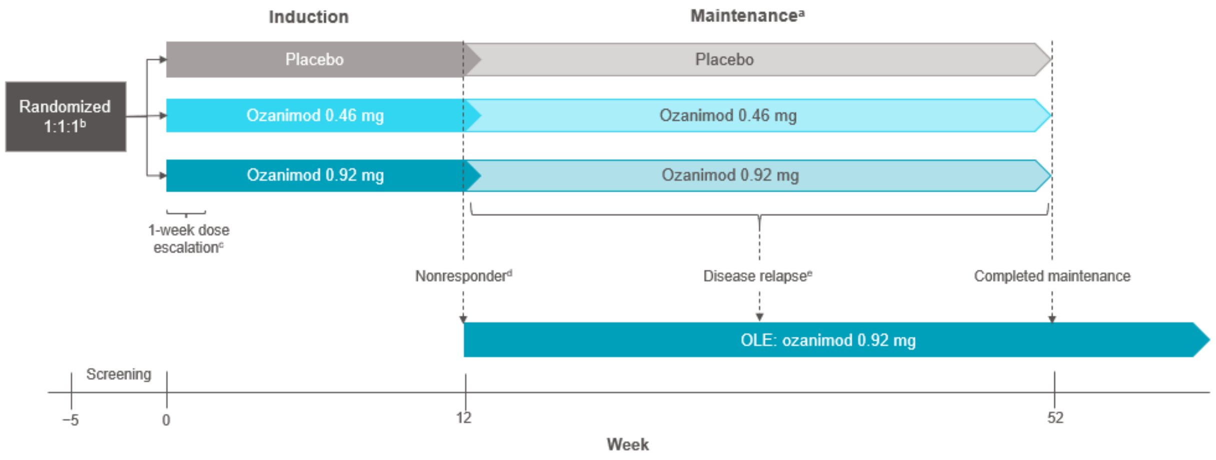

Supplementary Figure 2. Additional secondary and exploratory endpoints at weeks 12 and 52. Nonresponder imputation approach was used for handling of missing data. Weighted differences, 95% CIs, and *P* values for comparison between groups were based on the Cochran-Mantel-Haenszel test and were stratified by prior biologic agents and corticosteroid use (yes/no). [a]Clinical response: A reduction from baseline in the 9-point Mayo score of ≥2 points and ≥35%, and a reduction from baseline in the RBS of ≥1 point or an absolute RBS of ≤1 point. [b]Clinical remission: complete Mayo score of ≤2 points and with no individual subscore of >1 point. [c]Clinical remission: SFS = 0 or 1 (without a requirement of a decrease of ≥1 point from the baseline SFS), RBS = 0, and endoscopy subscore = 0 or 1. [d]Histologic remission: Geboes score <2.0. CI, confidence interval; RBS, rectal bleeding subscore; SFS, stool frequency subscore.

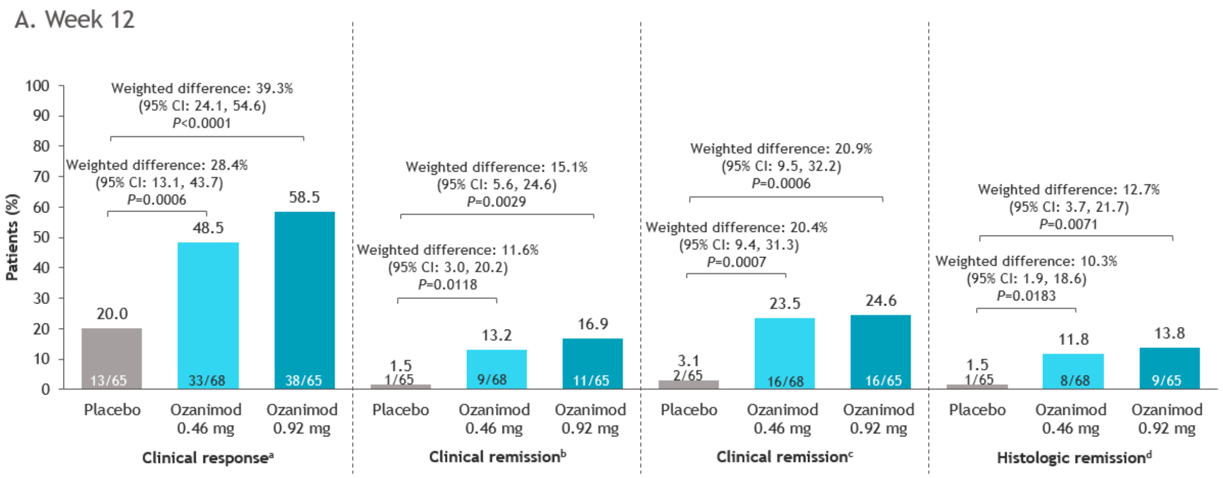

B. Week 52

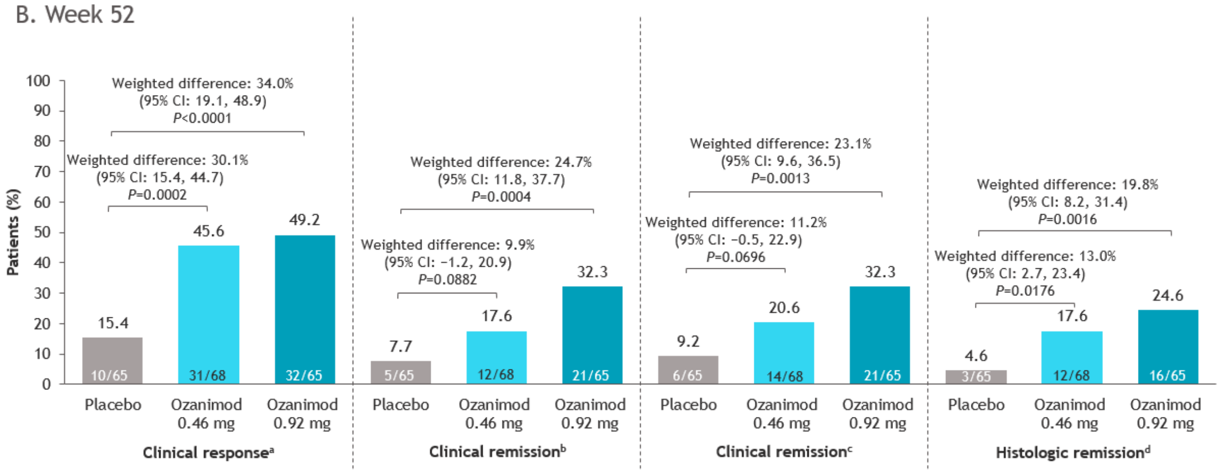
Patients (%)
100
90
80
70
60
50
40
30
20
10
0
Weighted difference: 34.0%
(95% CI: 19.1, 48.9)
P<0.0001
Weighted difference: 30.1%
(95% CI: 15.4, 44.7)
P=0.0002
15.4
45.6
49.2
10/65
31/68
32/65
Placebo
Ozanimod 0.46 mg
Ozanimod 0.92 mg
Clinical response[a]
Weighted difference: 24.7%
(95% CI: 11.8, 37.7)
P=0.0004
Weighted difference: 9.9%
(95% CI: −1.2, 20.9)
P=0.0882
7.7
17.6
32.3
5/65
12/68
21/65
Placebo
Ozanimod 0.46 mg
Ozanimod 0.92 mg
Clinical remission[b]
Weighted difference: 23.1%
(95% CI: 9.6, 36.5)
P=0.0013
Weighted difference: 11.2%
(95% CI: −0.5, 22.9)
P=0.0696
9.2
20.6
32.3
6/65
14/68
21/65
Placebo
Ozanimod 0.46 mg
Ozanimod 0.92 mg
Clinical remission[c]
Weighted difference: 19.8%
(95% CI: 8.2, 31.4)
P=0.0016
Weighted difference: 13.0%
(95% CI: 2.7, 23.4)
P=0.0176
4.6
17.6
24.6
3/65
12/68
16/65
Placebo
Ozanimod 0.46 mg
Ozanimod 0.92 mg
Histologic remission[d]

Supplementary Figure 3. Clinical response at week 12 by baseline characteristic subgroups. Nonresponder imputation approach is used for handling missing data. Treatment differences and *P* values for comparison between the active and placebo groups are based on the Cochran-Mantel-Haenszel test, stratified by prior biologic agents and CS use (yes or no). If the subgroup is the stratification factor, the Cochran-Mantel-Haenszel test is not stratified by this subgroup factor. 5-ASA, 5-aminosalicylic acid; ALC, absolute lymphocyte count; BL, baseline; CI, confidence interval; CS, corticosteroid; UC, ulcerative colitis.

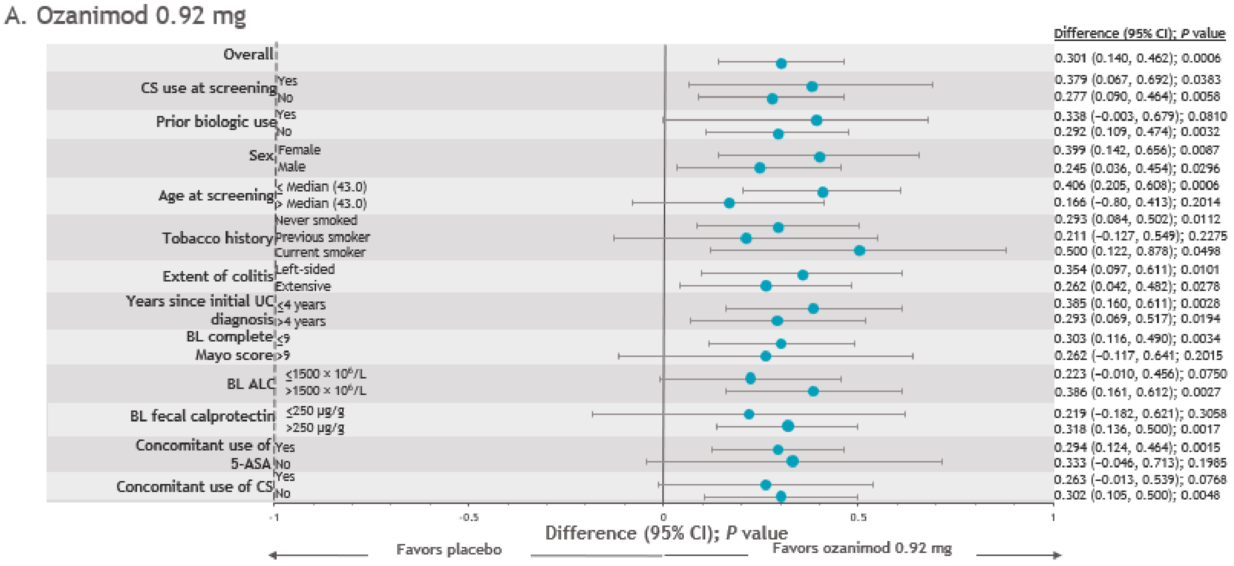

B. Ozanimod 0.46 mg

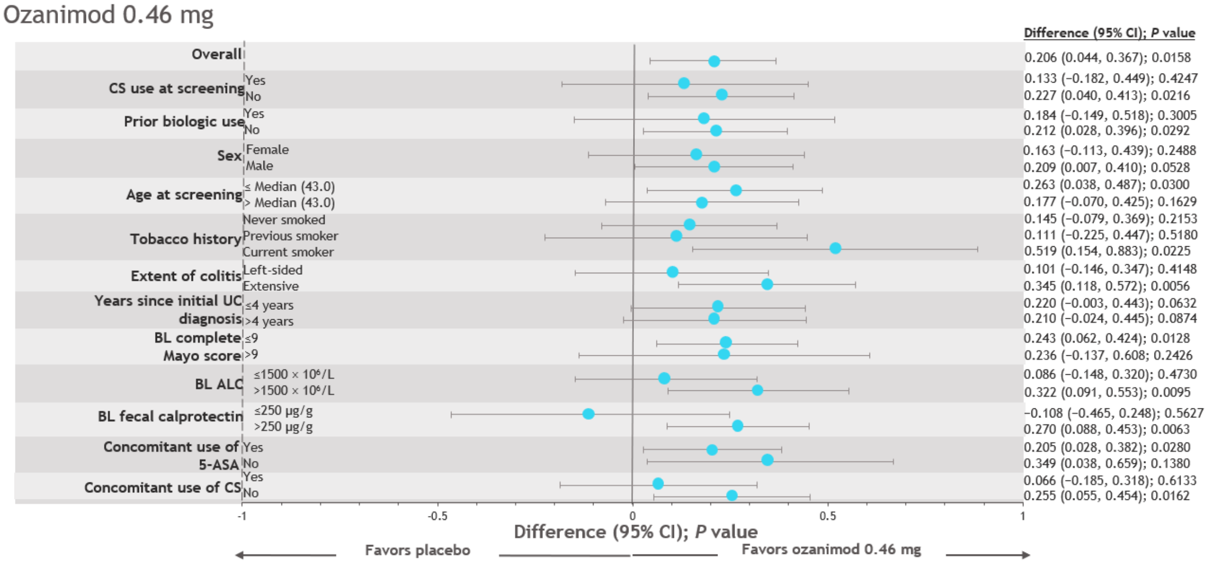

Supplementary Figure 4. Changes in absolute lymphocyte count over time. ALC, absolute lymphocyte count; SD, standard deviation; W, week.

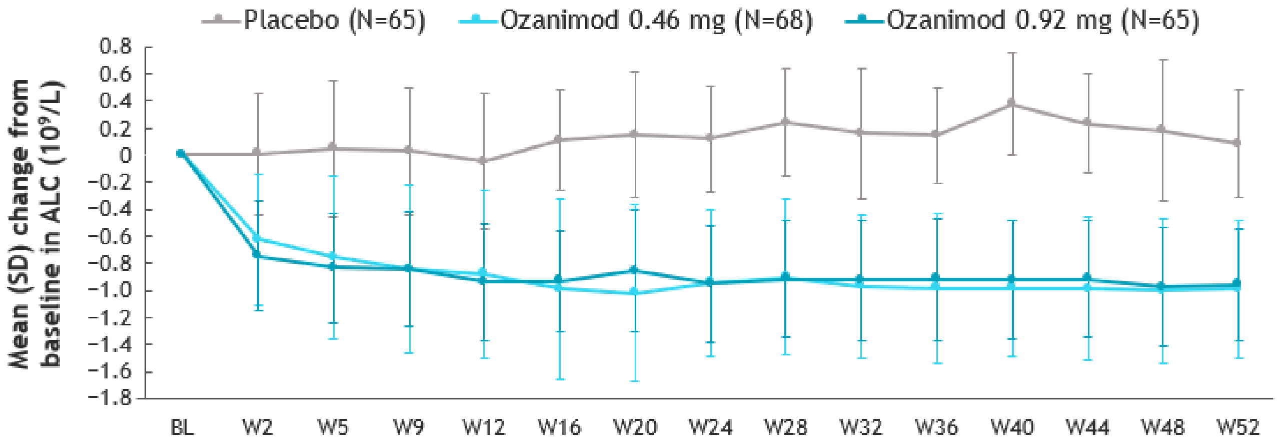

n	BL	W2	W5	W9	W12	W16	W20	W24	W28	W32	W36	W40	W44	W48	W52
Placebo	65	62	61	59	58	17	16	15	14	13	13	13	13	13	12
Ozanimod 0.46 mg	68	63	61	60	59	37	37	36	36	36	36	36	35	35	34
Ozanimod 0.92 mg	65	62	61	59	59	39	39	38	37	36	36	36	36	35	35

Plain Language Summary

Publication Plain Language Summary

Ozanimod Was Effective and Well Tolerated in Japanese Patients With Moderate to Severe Ulcerative Colitis

The full title of the article is "Once-Daily Oral Ozanimod for Japanese Patients With Ulcerative Colitis: Results From the Phase 2/3 J-True North Study"

You can find the full article here: [XXX]. You can access the full article for free

This publication plain language summary has been developed to accompany the article and is not intended for any other use.

What did this study look at?

Ulcerative colitis: What is it?

- Ulcerative colitis is a disease in which the lining of the colon and rectum becomes inflamed. Symptoms of ulcerative colitis include blood in the stool, diarrhea, and a feeling that you need to pass stools even though your bowels are already empty

People with untreated ulcerative colitis have a higher risk for

Anxiety and depression

Cancer of the colon and rectum

This disease can also negatively affect a person's job and social interactions

- To treat ulcerative colitis, doctors commonly prescribe anti-inflammatory medications, such as aminosalicylates and corticosteroids, as the first step. Medications known as immunomodulators, which reduce inflammation by decreasing the immune system response that starts the process of inflammation, may also be prescribed. If those medications do not work, medications known as biologics and Janus kinase inhibitors may be prescribed

The study: What is it about and why was it done?

- The efficacy and safety of ozanimod have not yet been evaluated in a large number of Japanese people
- Therefore, researchers conducted this J-True North study to see how once-daily ozanimod 0.46 mg or ozanimod 0.92 mg works in Japanese people (hereafter referred to as participants) with moderate to severe ulcerative colitis and to see what side effects it caused
- Based on findings from J-True North, once-daily ozanimod 0.92 mg was approved in Japan in December 2024 for the treatment of moderate to severe ulcerative colitis in participants who have had an inadequate response to standard treatments

Ozanimod: How does it work?

- Ozanimod is a sphingosine 1-phosphate (S1P) receptor 1 and 5 modulator that works to reduce inflammation by preventing lymphocytes, a kind of white blood cell, from moving into inflamed tissues

Ozanimod is a capsule taken by mouth

Participants who start ozanimod need to slowly increase the dose of ozanimod over a week to reach the once-daily dose of 0.92 mg

Ozanimod 0.23 mg once daily	Ozanimod 0.46 mg once daily	Ozanimod 0.92 mg once daily
Days 1-4	Days 5-7	Day 8 and beyond

The dose of ozanimod is increased slowly to reduce the chance of side effects on the heart.

- Ozanimod is approved to treat moderate to severe ulcerative colitis and multiple sclerosis in the United States and several other countries

The summary: What will this cover?

- This plain language summary describes a clinical study called J-True North, which looked at the effects of ozanimod in Japanese people with moderate to severe ulcerative colitis. This study compared changes in symptoms between people who took placebo and ozanimod to see if ozanimod worked. The placebo looked like ozanimod but did not have the active medication in it. Researchers also studied the side effects of ozanimod. This allowed them to determine whether ozanimod is safe and if it improved symptoms, such as blood in the stool and diarrhea

How was the study done?

Study start and end date
June 3, 2019, to August 28, 2023

Phase of study
Phase 2/3

Study status
Results reported within the study are final

Key inclusion criteria

- Japanese adults (18-75 years old) with ulcerative colitis for at least the past 3 months
- These adults must have previously taken aminosalicylates or corticosteroids to treat their ulcerative colitis

Key exclusion criteria

- People were excluded if they had serious inflammation of a large portion of their colon, known as severe extensive colitis, or if they had Crohn's disease, certain heart conditions, or a history of type 1 diabetes or uncontrolled type 2 diabetes

Randomization: Participants with ulcerative colitis were randomly assigned (or "randomized") to take once-daily ozanimod 0.46 mg, ozanimod 0.92 mg, or placebo

The placebo looks like ozanimod, but it does not have any medicine in it

This study was "blinded," meaning that participants in the study and the researchers did not know who took ozanimod and who took placebo

Screening
(5 weeks)

Researchers checked to see whether people with ulcerative colitis could take part in this study based on the study's inclusion and exclusion criteria

Induction period
(12 weeks)

Participants with ulcerative colitis took ozanimod or placebo for 12 weeks

After the 12 weeks, participants with ulcerative colitis who had clinical response, meaning whose symptoms were better, could enter the maintenance period of the study

Maintenance period
(40 weeks)

Participants with ulcerative colitis continued to take the same medicine (ozanimod or placebo) as they did during the induction period for an additional 40 weeks

Week −5 | 0 | 12 | 52

In this study, researchers determined whether people with ulcerative colitis had clinical response by seeing whether their symptoms improved (for example, by having less blood in the stool, less diarrhea, or less abdominal pain) and by using a small camera to see whether the colon looked less swollen

- The study looked at the percentages of participants with clinical response after 12 weeks of treatment and after 52 weeks of taking ozanimod
- These percentages of participants with clinical response after taking ozanimod were compared with the percentages of participants with clinical response after taking placebo
- Researchers also looked at the side effects that participants had during the study

Who took part in this study?

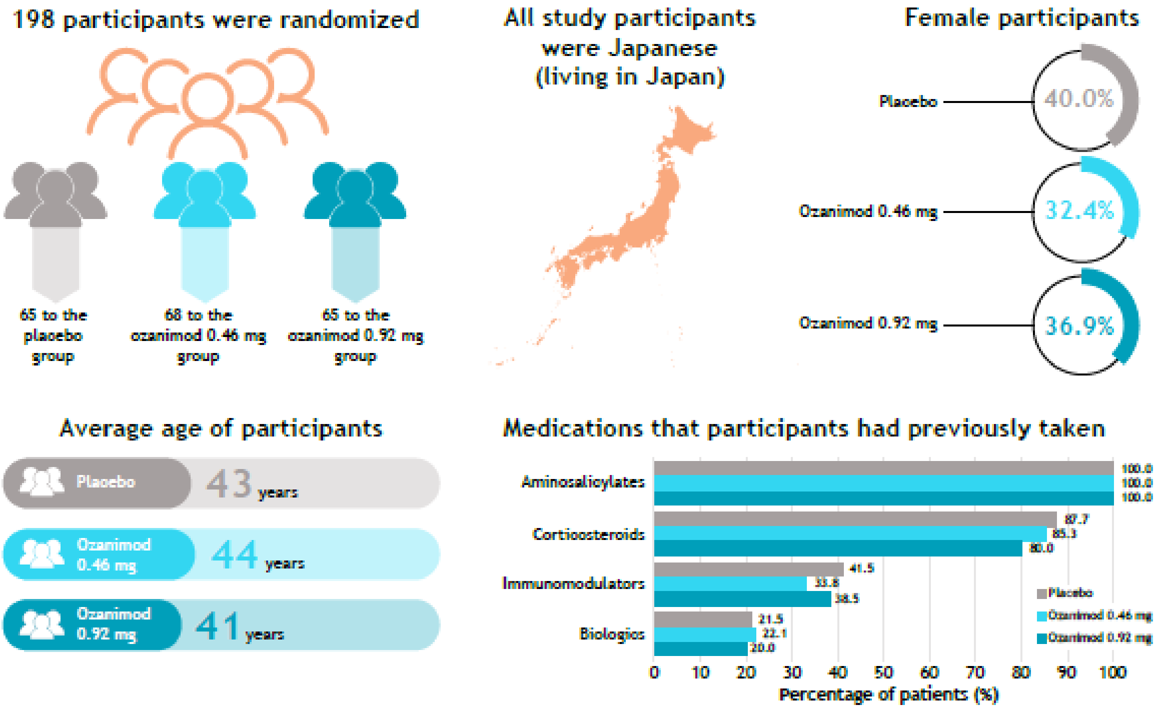

What were the results of the study?

- After taking ozanimod for **12 weeks** (either dose), **more than half of the participants had clinical response** compared with less than one-third of those in the placebo group

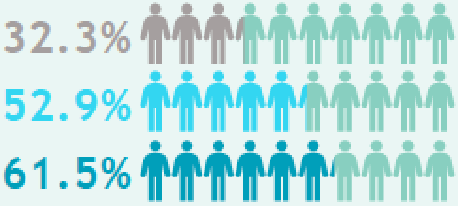

- After taking ozanimod for **52 weeks** (either dose), **almost half of the people had clinical response** compared with only 16.9% in the placebo group

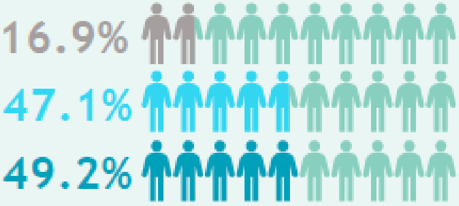

- ≥80% of participants who achieved clinical response receiving ozanimod 0.46 mg or ozanimod 0.92 mg at the end of the induction period showed clinical response at the end of the maintenance period

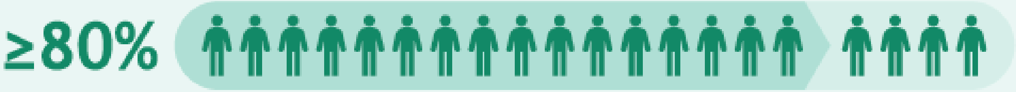

- In the induction and maintenance periods of the study, the following were the **most common side effects** in participants taking ozanimod:
- Researchers were interested in knowing whether participants taking ozanimod had **side effects** seen in patients who took S1P modulators, the same class of medications as ozanimod

	Cold symptoms	Fever	Headache	Low heart rate	Cancer	Shingles	Swelling in part of the retina
Placebo	9.2%	4.6%	6.2%	None	None	1.5%	None
Ozanimod 0.46 mg	14.7%	16.2%	11.8%	None	None	1.5%	None
Ozanimod 0.92 mg	13.8%	10.8%	9.2%	None	None	3.1%	1.5%

What were the study's main conclusions?

- Ozanimod was effective and well tolerated as a once-daily oral medication in Japanese participants with moderate to severe ulcerative colitis
- This Japanese clinical study was the first large-scale study examining ozanimod in an Asian population. The results suggest that ozanimod is effective and safe for Asians
- The efficacy and safety results of ozanimod in the J-True North study were similar to the findings from a global study of ozanimod called True North

Who sponsored this study?

This clinical study was sponsored by Bristol Myers Squibb, which thanks everybody who participated in the study

Where can I find additional information?

The original article was published in the medical journal *Gastro Hep Advances*. Please refer to the original article for complete author-disclosure information

You can find more information about this study here:
Study Details | To Evaluate Efficacy and Long-term Safety of Ozanimod in Japanese Subjects With Moderately to Severely Active Ulcerative Colitis | https://clinicaltrials.gov/study/NCT03915769
