# Supplementary material for: Once-Daily Oral Ozanimod for Japanese Patients With Ulcerative Colitis: Results From the Phase 2/3 J-True North Study
Source: Gastro Hep Adv. 2025 Sep 16;5(1):100812. doi: 10.1016/j.gastha.2025.100812 (PMC12630022; doi:10.1016/j.gastha.2025.100812)
Supplement: Extended PDF [file mmc2.pdf]

Gastro Hep Advances 2026;5:100812

ORIGINAL RESEARCH—CLINICAL

Once-Daily Oral Ozanimod for Japanese Patients With Ulcerative Colitis: Results From the Phase 2/3 J-True North Study

Hiroshi Nakase,[1] Toshimitsu Fujii,[2] Tadakazu Hisamatsu,[3] Yasuo Suzuki,[4] Mamoru Watanabe,[5] Sakuma Takahashi,[6] Makoto Ooi,[7] Ken Takeuchi,[8] Tsuguhiro Kimura,[9] Ken Furuya,[10] Nobuo Aoyama,[11] Kenkei Hasatani,[12] Noriyuki Horiki,[13] Kazunari Kanke,[14] Satoki Tokito,[15] Souken Sai,[16] Yoko Uchikawa,[17] Shoichiro Goto,[17] Go Fujimoto,[17] Changliang Zhang,[17] AnnKatrin Petersen,[18] and Toshifumi Hibi[19]

[1]Sapporo Medical University, Sapporo, Japan; [2]Institute of Science Tokyo, Tokyo, Japan; [3]Kyorin University School of Medicine, Mitaka, Japan; [4]Ginza Central Clinic, Tokyo, Japan; [5]Graduate School of Medicine, Juntendo University, Tokyo, Japan; [6]Kagawa Prefectural Central Hospital, Takamatsu, Japan; [7]Division of Gastroenterology, Department of Internal Medicine, Graduate School of Medicine, Kobe University, Kobe, Japan; [8]Tsujinaka Hospital Kashiwanoha, Kashiwa, Japan; [9]Medical Corporation Shoyu-Kai Fujita Gastroenterology Hospital, Takatsuki, Japan; [10]JCHO Hokkaido Hospital, Sapporo, Japan; [11]Aoyama Medical Clinic GI Endoscopy & IBD Center, Kobe, Japan; [12]Fukui Prefectural Hospital, Fukui, Japan; [13]Mie University Hospital, Tsu, Japan; [14]Kanke Gastrointestinal Clinic, Utsunomiya, Japan; [15]Tokitokai Tokito Clinic, Saitama, Japan; [16]Sai Gastroenterology and Proctology Clinic, Fujiidera, Japan; [17]Bristol Myers Squibb, Tokyo, Japan; [18]Bristol Myers Squibb, Princeton, New Jersey; and [19]Kitasato University Kitasato Institute Hospital, Tokyo, Japan.

BACKGROUND AND AIMS: Ozanimod is a once-daily, oral, selective sphingosine 1-phosphate receptor 1 and 5 modulator. The objective of the randomized, phase 2/3 J-True North study (NCT03915769) was to assess the efficacy and safety of ozanimod in Japanese patients with moderately to severely active ulcerative colitis. **METHODS:** In the 12-week induction period (IP), patients were randomized 1:1:1 to receive placebo, ozanimod 0.46 mg, or ozanimod 0.92 mg. Patients who completed the IP with a clinical response at week (w) 12 were eligible to enter a 40-week maintenance period where they received the same treatment as they did in the IP. The primary endpoint was clinical response (complete Mayo score) at w12; clinical and mucosal secondary endpoints were assessed at w12 and w52. **RESULTS:** Of 198 patients randomized, 176 completed the IP. Of these patients, 97 entered and 77 completed the maintenance period. A significantly higher proportion of patients receiving ozanimod achieved clinical response at w12 versus placebo (ozanimod 0.46 mg: 52.9%, $P = .0158$; ozanimod 0.92 mg: 61.5%, $P = .0006$; vs placebo: 32.3%). Similar results were observed in the secondary endpoints where patients receiving ozanimod achieved higher rates of clinical remission, endoscopic improvement, and mucosal healing at w12 than those receiving placebo. Efficacy was maintained at w52 for all endpoints. Both doses of ozanimod were well tolerated, with no unexpected safety signals. **CONCLUSION:** This large-scale clinical trial demonstrated the efficacy and safety of once-daily oral ozanimod in Japanese patients with moderately to severely active ulcerative colitis. This is the first time that the efficacy and safety of ozanimod were verified in a large number of patients in Asia.

Keywords: Clinical Trial; Japan; Ozanimod; Sphingosine 1-Phosphate; Ulcerative Colitis

Introduction

Ulcerative colitis (UC) is an immune-mediated disease with unknown etiology characterized by an increased migration and accumulation of lymphocytes in the inflamed tissues of the colon and rectum.[1,2] Although UC has a higher prevalence in the West, it is increasing in Asia, especially in Japan.[3]

Patients with UC are commonly treated with standard therapies (eg, 5-aminosalicylic acid, corticosteroids, immunomodulators), often followed by advanced therapies (ie, biologics [eg, anti-tumor necrosis factor agents, anti-integrins, anti-interleukin 12/23], and Janus kinase [JAK]

Abbreviations used in this paper: **AE, adverse event; ALC, absolute lymphocyte count; CRP, C-reactive protein; FCP, fecal calprotectin; IgG, immunoglobulin G; IP, induction period; MP, maintenance period; MS, multiple sclerosis; OLE, open-label extension; RBS, rectal bleeding subscore; S1P, sphingosine 1-phosphate; SAE, serious adverse event; SFS, stool frequency subscore; TEAE, treatment-emergent adverse event; UC, ulcerative colitis.**

Most current article

Copyright © 2025 The Authors. Published by Elsevier Inc. on behalf of the AGA Institute. This is an open access article under the CC BY license (http://creativecommons.org/licenses/by/4.0/).
2772-5723
https://doi.org/10.1016/j.gastha.2025.100812

inhibitors) if UC is uncontrolled with standard treatment.[1,4–6] Despite the advancements in treatments for UC, there is still a need for novel advanced therapies due to the overuse of corticosteroids and parenteral administration, limited efficacy (ie, primary nonresponse or loss of response over time), and safety profiles associated with current advanced therapies.[7–15]

Unlike most advanced therapies that target cytokine signaling,[16–18] sphingosine 1-phosphate (S1P) receptor modulators, such as ozanimod, primarily regulate lymphocyte trafficking.[2,19] Ozanimod is an oral small molecule that selectively binds to $S1P_1$ and $S1P_5$ receptors, causing internalization of $S1P_1$ receptors on lymphocyte surfaces to prevent S1P-dependent lymphocyte egression from lymph nodes to inflamed tissues.[2] Given the role of S1P receptors on lymphocyte trafficking, targeting these receptors has proven effective in treating immune-mediated diseases, such as multiple sclerosis (MS) and UC.[2,20,21] The efficacy and/or safety of ozanimod 0.92 mg for the treatment of moderately to severely active UC was demonstrated in the phase 2 TOUCHSTONE study[20] and the subsequent pivotal phase 3 True North study.[22] Ozanimod was approved for the treatment of relapsing MS in 2020 and moderately to severely active UC in 2021 in the United States and several other countries.[23–28]

This phase 2/3 Japan (J)-True North study compared the efficacy and safety of once-daily ozanimod 0.46 or 0.92 mg with placebo in Japanese patients with moderately to severely active UC. Based on the findings from J-True North, once-daily ozanimod 0.92 mg was approved for the treatment of UC in Japan in December 2024.[29] Herein, we report the J-True North efficacy and safety results for ozanimod in Japanese patients with moderately to severely active UC.

Methods

Patients

Japanese patients aged 18–75 years with a diagnosis of UC ≥3 months before receiving study treatment, evidence of UC extending ≥15 cm from the anal verge by baseline endoscopy, and moderately to severely active UC (defined as Mayo score of 6–12 with a Mayo endoscopy subscore ≥2, a rectal bleeding subscore [RBS] ≥1, and a stool frequency subscore [SFS] ≥1) were included. Patients must have been previously exposed to aminosalicylates or corticosteroids, and those receiving treatment of either at enrollment needed to continue treatment during the induction period. In addition, patients needed to have documentation of positive varicella zoster virus immunoglobulin G (IgG) antibody status or must have completed varicella zoster virus vaccination ≥30 days before randomization. Patients were excluded if they had severe extensive colitis, Crohn's disease or intermediate colitis, clinically relevant cardiovascular conditions, a history of type 1 diabetes or uncontrolled type 2 diabetes, or a history of uveitis or macular edema. Full inclusion and exclusion criteria are included in the Supplementary Methods.

Study Design

J-True North was a multicenter, double-blind, placebo-controlled, parallel-group, 2-dose, randomized phase 2/3 study. A list of J-True North investigators, sites, institutional review boards or ethical review boards, and chairpersons are reported in Table A1.

Following up to 5 weeks of screening, patients were randomized 1:1:1 using interactive-response technology to receive once-daily ozanimod 0.46 mg, ozanimod 0.92 mg, or placebo, stratified by corticosteroid use at screening and prior use of biologics, in the 12-week induction period. The study included patients with or without prior biologic exposure, with the proportion of those previously treated with biologics limited to approximately 30%. Patients randomized to ozanimod initiated a 7-day dose escalation upon treatment initiation: ozanimod 0.23 mg on days 1–4, ozanimod 0.46 mg on days 5–7, and the assigned ozanimod treatment dose (0.46 mg or 0.92 mg) thereafter. Patients who achieved clinical response at week 12 were eligible to enter the 40-week maintenance period, during which patients continued to receive the same treatment. Patients were eligible to enter an open-label extension (OLE) to receive once-daily ozanimod 0.92 mg if they were clinical nonresponders at week 12, experienced disease relapse during the maintenance period, or completed the maintenance period (Figure A1, Supplementary Methods).

The patient, investigator, and study-site personnel were blinded to the treatment received by each patient. A patient's treatment assignment was kept blinded to the investigators until after the last randomized patient completed their last visit in the maintenance period, unless subsequent medical treatment for the patient required knowledge of the assigned treatment.

Assessments and Outcomes

Efficacy. Endoscopy and biopsy samples were evaluated in a blinded manner by a qualified central laboratory. Patient-reported outcomes (ie, SFS and RBS) and the clinician-reported Physician Global Assessment were collected in an electronic diary. All efficacy endpoints are defined in Table A2.

The primary endpoint was the proportion of patients with clinical response (ie, a reduction from baseline in the complete Mayo score [sum of SFS, RBS, endoscopy subscore, and Physician Global Assessment, with each assessment rated on a scale of 0–3] ≥3 points and ≥30%, and a reduction from baseline in the RBS of ≥1 or an absolute RBS of ≤1 point) at week 12.

Secondary efficacy endpoints, listed in no particular order, included the proportion of patients who achieved clinical response (complete Mayo score) at week 52 and the proportion of patients who achieved clinical response (9-point Mayo score), clinical remission (based on definitions 1 and 2 in Table A2), endoscopic improvement, and mucosal healing at weeks 12 and 52. The proportion of patients who achieved histologic remission and clinical remission (based on definition 3 in Table A2) at weeks 12 and 52 were also explored.

Pharmacodynamics

Absolute lymphocyte count (ALC), C-reactive protein (CRP), and stool analysis for fecal calprotectin (FCP) were assessed throughout the study.

Safety

Treatment-emergent adverse events (TEAEs), serious adverse events (SAEs), TEAEs leading to discontinuation, and adverse events of special interest (eg, bradycardia, heart conduction abnormalities, serious infections, malignancies, macular edema, hepatic effects) were assessed through J-True North. Clinical laboratory evaluations were completed by a central laboratory. During the blinded treatment period, white blood cell differential results were available to an unblinded medical reviewer independent from the study. Vital signs, electrocardiograms (before first dose and 6 hours after the first dose), and optical coherence tomography (for at-risk patients with a history of uveitis, diabetes mellitus, or underlying or coexisting retinal disease) were also completed during the study.

Statistical Analysis

Baseline patient characteristics and demographics were summarized descriptively. The intention-to-treat population, which was the primary population for efficacy analyses, included all randomized patients from the screened population who received $\geq$1 dose of study treatment. The primary endpoint of clinical response at week 12 was analyzed using the Cochran-Mantel-Haenszel test stratified by corticosteroid use at screening and by prior biologic use. Pairwise comparisons were performed between each ozanimod group and the placebo group. Other binary efficacy endpoints were similarly analyzed. Missing data were handled using nonresponder imputation analyses, which included patients who discontinued the study before a given timepoint and patients who met prespecified criteria for treatment failures before the timepoint and were considered nonresponders at that timepoint. Continuous endpoints were analyzed by analysis of covariance models adjusted for corticosteroid use (yes or no) at screening, prior biologic use (yes or no), and the baseline value of the corresponding variable.

To account for multiplicity for the primary endpoint, ozanimod 0.92 mg versus placebo comparison in the primary analysis was tested using a 2-sided test with an alpha of 0.05 level of significance. When this comparison was statistically significant ($P \leq .05$), ozanimod 0.46 mg versus placebo comparisons were further performed. Due to no statistical hypotheses, secondary and exploratory efficacy endpoints were tested in a nonhierarchical fashion without multiplicity adjustment; therefore, reported *P* values were nominal and were provided as a measure of strength of association between the endpoint and treatment effect for the exploratory purpose. By using a 2-sided chi-square test for 2-sample hypothesis testing at an alpha of 0.05 with a 90% power, the estimated sample size needed to detect a difference of 28% between ozanimod 0.92 mg versus placebo was 65 patients per treatment group, or a total of 195 patients.

Ethical Considerations

J-True North adhered to the Good Clinical Practices guidelines and was conducted in accordance with the ethical principles outlined in the Declaration of Helsinki. Before study initiation, the study protocol and informed consent were approved at each study site by an institutional review board or independent ethics committee. Written informed consent was obtained from each patient prior to entering the study and before the initiation of any trial-related procedure. J-True North was sponsored by Bristol Myers Squibb.

Results

Patient Disposition and Baseline Characteristics

From June 3, 2019, to August 28, 2023, a total of 263 patients were enrolled and screened (Figure 1). Of these patients, 198 were randomized: 65 to placebo, 68 to ozanimod 0.46 mg, and 65 to ozanimod 0.92 mg. Most patients (n = 176 [88.9%]) completed the 12-week induction period (placebo: n = 59 [90.8%]; ozanimod 0.46 mg: n = 59 [86.8%]; ozanimod 0.92 mg: n = 58 [89.2%]). The most common reasons for treatment discontinuation in the induction period were lack of efficacy (n = 8 [4.0%]), adverse event (AE; n = 5 [2.5%]), and withdrawal by patient (n = 3 [1.5%]).

A total of 97 (49.0%) patients who achieved clinical response at week 12 entered the maintenance period (placebo: n = 20 [30.8%]; ozanimod 0.46 mg: n = 37 [54.4%]; ozanimod 0.92 mg: n = 40 [61.5%]). Of these patients, 77 completed the maintenance period (placebo: n = 12 [18.5%]; ozanimod 0.46 mg: n = 30 [44.1%]; ozanimod 0.92 mg: n = 35 [53.8%]). For the 19 patients who discontinued treatment in the placebo or ozanimod groups, common reasons for discontinuation included disease relapse (n = 12 [6.1%]), AE (n = 3 [1.5%]), and withdrawal by patient (n = 2 [1.0%]). At the time of data cutoff (August 28, 2023), 168 patients entered the OLE: 79 (47.0%) from the induction period, 12 (7.1%) who relapsed during the maintenance period, and 77 (45.8%) who completed the maintenance period.

The baseline demographic and disease characteristics were well balanced across the 3 treatment groups (Table 1). Overall, more patients were male, the mean age was approximately 43 years, the mean total Mayo score at baseline was 8.4, and 21.2% of patients were previously exposed to biologics.

Efficacy Outcomes in the Induction Period

At week 12, the percentages of patients who achieved clinical response (complete Mayo score), the primary endpoint, were significantly higher in both ozanimod groups (ozanimod 0.46 mg: 52.9%, $P = .0158$; ozanimod 0.92 mg: 61.5%, $P = .0006$) than in the placebo group (32.3%) (Figure 2). Greater proportions of patients achieved clinical remission (ozanimod 0.46 mg: 17.6%, $P = .0021$; ozanimod 0.92 mg: 24.6%, $P = .0002$; vs placebo: 1.5%) and endoscopic improvement (ozanimod 0.46 mg: 27.9%, $P = .0027$; ozanimod 0.92 mg: 29.2%, $P = .0023$; vs placebo: 7.7%) with ozanimod than placebo at week 12 (Figure 2). Likewise, higher proportions of patients achieved mucosal healing at week 12 with ozanimod versus placebo (ozanimod 0.46 mg: 5.9%, $P = .1870$; ozanimod 0.92 mg: 7.7%, $P = .0884$; vs placebo: 1.5%) (Figure 2).

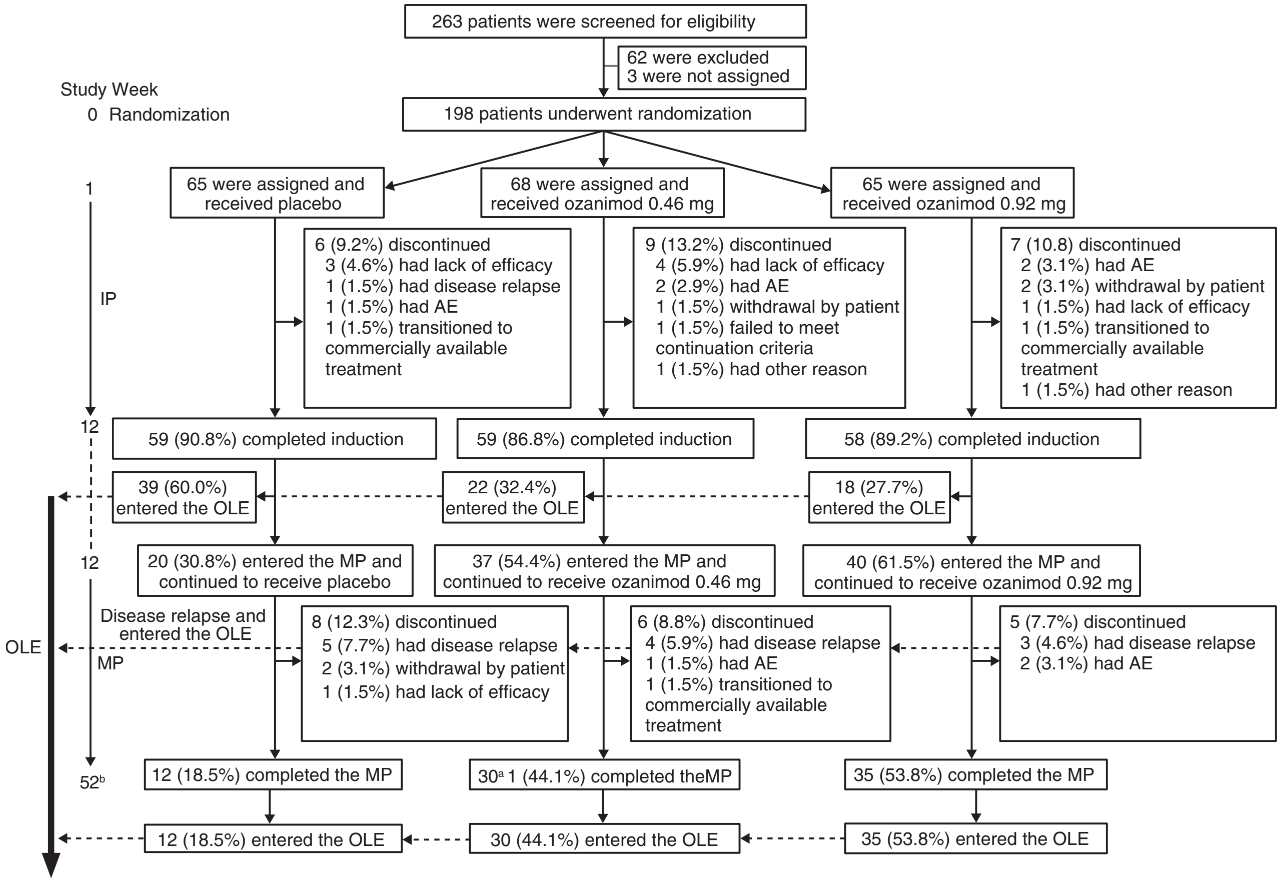

Figure 1. Patient disposition. Percentages are based on the randomized population. [a]One patient completed the week 52 study visit but was still on treatment in the MP at the time of the data cutoff date; all data for this patient up to week 52 were included in the analysis. [b]Duration of the MP was shortened to 40 weeks (total of 52 weeks) after the study protocol was amended from the original 52 weeks (total of 64 weeks). The timepoint of MP efficacy endpoints was week 52 throughout the study. AE, adverse event; IP, induction period; MP, maintenance period; OLE, open-label extension.

The efficacy of ozanimod at week 12 remained consistent when using alternate definitions of clinical response and clinical remission (Figure A2A). A higher proportion of patients achieved histologic remission at week 12 with both ozanimod dose groups compared with placebo (ozanimod 0.46 mg: 11.8%, P = .0183; ozanimod 0.92 mg: 13.8%, P = .0071; vs placebo: 1.5%) (Figure A2A).

In a prespecified subgroup analysis of the primary endpoint, ozanimod 0.92 mg was effective regardless of prior biologic experience or concomitant corticosteroid use; however, those without concomitant corticosteroid use demonstrated better efficacy than those with concomitant corticosteroid use (Figure A3A). Clinical response rates favored ozanimod 0.92 mg over placebo in most baseline characteristic subgroups (Figure A3A), and similar patterns were seen in the ozanimod 0.46 mg group (Figure A3B).

Efficacy Outcomes in the Maintenance Period

Compared with placebo, a higher proportion of patients in both ozanimod groups achieved clinical response at week 52 in the maintenance period (ozanimod 0.46 mg: 47.1%, P = .0002; ozanimod 0.92 mg: 49.2%, P = .0001; vs placebo: 16.9%) (Figure 3). Notably, ≥80% of patients who achieved clinical response receiving ozanimod 0.46 or ozanimod 0.92 mg at the end of the induction period also showed clinical response at the end of the maintenance period.

At week 52, rates of clinical remission were greater with ozanimod 0.46 mg (17.6%, P = .0883) and with ozanimod 0.92 mg (29.2%, P = .0019) than with placebo (7.7%) (Figure 3). A similar pattern was seen for endoscopic improvement and mucosal healing (Figure 3). These results were consistent when measured by alternate definitions of clinical response and remission (Figure A2B). Rates of histologic remission were greater with ozanimod than placebo at week 52 (ozanimod 0.46 mg: 17.6%, P = .0176; ozanimod 0.92 mg: 24.6%, P = .0016; vs placebo: 4.6%) (Figure A2B). Although sample sizes were low, corticosteroid-free remission at week 52 was achieved in 0% (0/3), 25.0% (1/4), and 28.6% (2/7) of patients in the placebo, ozanimod 0.46 mg, and ozanimod 0.92 mg groups, respectively.

Table 1. Demographic and Clinical Characteristics at Baseline in the Induction Period (Intention-to-Treat Population)

Characteristic	Placebo (N = 65)	Ozanimod 0.46 mg (N = 68)	Ozanimod 0.92 mg (N = 65)	Total (N = 198)
Female, n (%)	26 (40.0)	22 (32.4)	24 (36.9)	72 (36.4)
Age, y, mean (SD)	42.5 (13.0)	43.9 (13.0)	41.4 (14.3)	42.6 (13.4)
Weight, kg, mean (SD)	62.5 (11.4)	62.7 (11.8)	62.8 (11.5)	62.7 (11.5)
BMI, kg/m^2, mean (SD)	22.7 (2.8)	22.5 (3.3)	22.5 (3.5)	22.6 (3.2)
Years since UC diagnosis, mean (SD)	8.3 (7.4)	7.0 (8.3)	6.3 (6.5)	7.2 (7.5)
Extent of UC disease, n (%)				
Left-sided	27 (41.5)	37 (54.4)	26 (40.0)	90 (45.5)
Extensive	38 (58.5)	31 (45.6)	39 (60.0)	108 (54.5)
Total mayo score, mean (SD)	8.5 (1.1)	8.4 (1.4)	8.3 (1.5)	8.4 (1.3)
9-Point mayo score,[a] mean (SD)	6.4 (1.0)	6.4 (1.2)	6.3 (1.4)	6.4 (1.2)
Fecal calprotectin, μg/g, median (range)	1060 (13–16,800)	1500 (27–15,200)	885 (20–22,200)	1130 (13–22,200)
Prior medication use, n (%)				
5-ASA	65 (100.0)	68 (100.0)	65 (100.0)	198 (100.0)
Corticosteroid	57 (87.7)	58 (85.3)	52 (80.0)	167 (84.3)
Immunomodulator	27 (41.5)	23 (33.8)	25 (38.5)	75 (37.9)
Biologics	14 (21.5)	15 (22.1)	13 (20.0)	42 (21.2)
Nonresponse to biologics,[b] n (%)				
Primary nonresponse	5 (7.7)	4 (5.9)	6 (9.2)	15 (7.6)
Secondary nonresponse	4 (6.2)	10 (14.7)	6 (9.2)	20 (10.1)

5-ASA, 5-aminosalicylic acid; BMI, body mass index; RBS, rectal bleeding subscore; SD, standard deviation; SFS, stool frequency subscore; UC, ulcerative colitis.
[a]The sum of RBS, SFS, and Mayo endoscopy subscore.
[b]Percentages are from the total patients in each treatment group.

Pharmacodynamics

Median FCP levels at baseline were 1060 μg/g in the placebo group, 1500 μg/g in the ozanimod 0.46 mg group, and 885 μg/g in the ozanimod 0.92 mg group. Greater decreases from baseline in FCP levels were seen with ozanimod treatment versus placebo at weeks 12 and 52

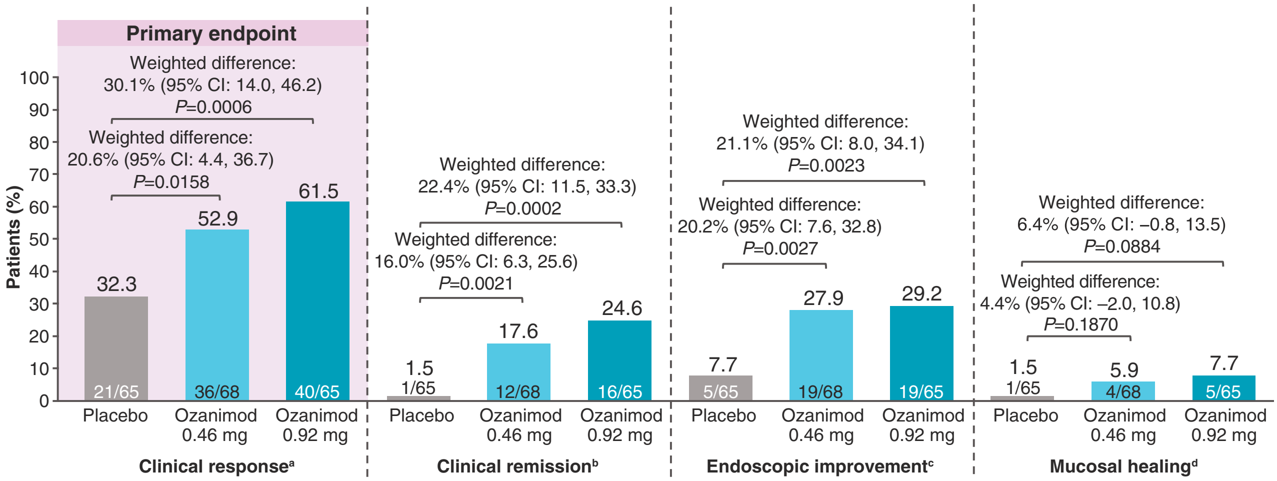

Figure 2. Proportion of patients who achieved efficacy endpoints at week 12. Nonresponder imputation approach was used for handling of missing data. Weighted differences, 95% CIs, and *P* values for comparison between groups were based on the Cochran-Mantel-Haenszel test and were stratified by prior biologic agents and corticosteroid use (yes/no). [a]Clinical response: reduction from baseline in total Mayo score of ≥3 points and ≥30%, and reduction from baseline in the RBS of ≥1 point or an absolute RBS of ≤1 point. [b]Clinical remission: RBS = 0 and SFS ≤1 (and a decrease of ≥1 point from baseline SFS) and MES ≤1 point. [c]Endoscopic improvement: MES ≤1 point. [d]Mucosal healing: MES ≤1 point and Geboes score <2.0. CI, confidence interval; MES, Mayo endoscopy subscore; RBS, rectal bleeding subscore; SFS, stool frequency subscore.

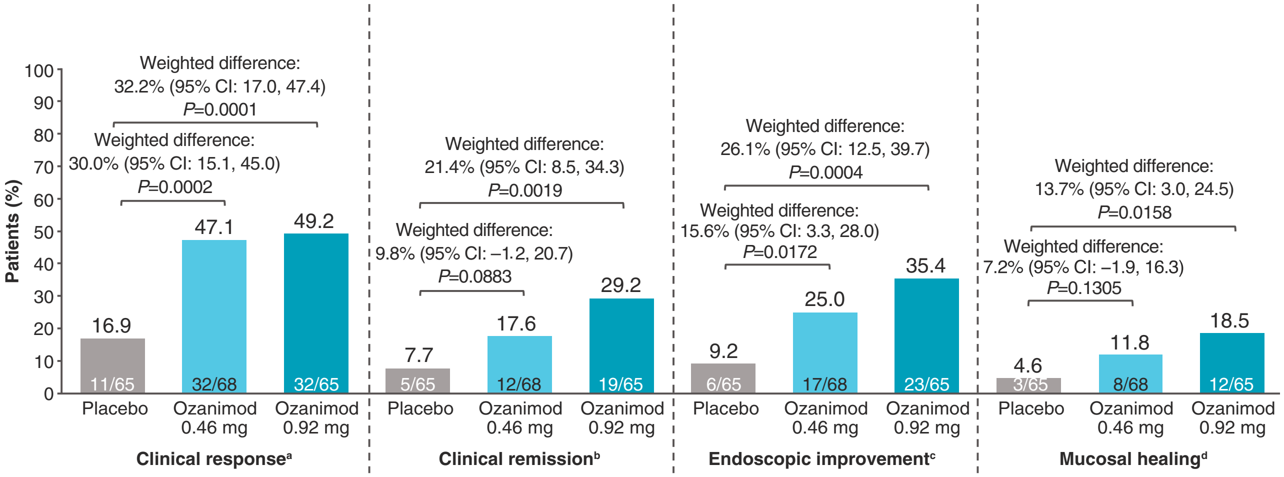

Figure 3. Proportions of patients who achieved efficacy endpoints at week 52. Nonresponder imputation approach was used for handling of missing data. Weighted differences, 95% CIs, and *P* values for comparison between groups were based on the Cochran-Mantel-Haenszel test and were stratified by prior biologic agents and corticosteroid use (yes/no). [a]Clinical response: reduction from baseline in total Mayo score of ≥3 points and ≥30%, and reduction from baseline in the RBS of ≥1 point or an absolute RBS of ≤1 point. [b]Clinical remission: RBS = 0 and SFS ≤1 (and a decrease of ≥1 point from baseline SFS) and MES ≤1 point. [c]Endoscopic improvement: MES ≤1 point. [d]Mucosal healing: MES ≤1 point and Geboes score <2.0. CI, confidence interval; MES, Mayo endoscopy subscore; RBS, rectal bleeding subscore; SFS, stool frequency subscore.

(Table A3). The proportions of patients whose FCP levels shifted from baseline to ≤50 μg/g, ≤100 μg/g, and ≤150 μg/g at week 12 were higher in both ozanimod groups compared with the placebo group (Table A4). Median CRP levels at baseline were similar in the 3 groups: placebo (1.4 mg/L), ozanimod 0.46 mg (1.7 mg/L), and ozanimod 0.92 mg (1.6 mg/L). Similar to FCP, greater decreases from baseline in CRP levels were seen with ozanimod than with placebo at weeks 12 and 52 (Table A3).

While ALCs were stable in the placebo group, those in the ozanimod 0.46 mg and 0.92 mg groups showed a decrease at week 2 (mean ALC change from baseline: -0.6×10^9/L and -0.7×10^9/L, respectively) and a plateau at week 5 (-0.8×10^9/L and -0.8×10^9/L) (Figure A4). The decreases in ALC observed during the induction period in both ozanimod groups were sustained throughout the maintenance period. Mean ALC decreased by 1.0×10^9/L in both ozanimod groups compared with an increase of 0.08×10^9/L in the placebo group from baseline to week 52. Leukocytes decreased by 0.2×10^9/L in the placebo group, 1.9×10^9/L in the ozanimod 0.46 mg group, and 2.0×10^9/L in the ozanimod 0.92 mg group from baseline to week 52 (Table A5). Slight decreases were seen for circulating neutrophils (placebo: 0.3×10^9/L; ozanimod 0.46 mg: 0.6×10^9/L; ozanimod 0.92 mg: 0.9×10^9/L). Only 1 patient (1.5%) in the placebo group had a total white blood cell count $>20 \times 10^9$/L after the initiation of treatment (Table A6).

Safety

The overall incidences of AEs were similar across all treatment groups in the induction period and greater in the ozanimod groups than in the placebo group during the induction and maintenance periods (Table 2). The incidences of SAEs in the induction and maintenance periods were 2 (3.1%) in the placebo group, 5 (7.4%) in the ozanimod 0.46 mg group, and 5 (7.7%) in the ozanimod 0.92 mg group. Treatment-related SAEs occurred in 1 patient in the placebo group and 1 patient in the ozanimod 0.46 mg group. TEAEs leading to treatment withdrawal occurred in 5.9% and 6.2% in the ozanimod 0.46 mg and 0.92 mg groups, respectively, and 3.1% in the placebo group in the induction and maintenance periods. TEAEs leading to ozanimod treatment withdrawal in the induction and maintenance periods included hemiplegia (1.5% [1/68]) in the ozanimod 0.46 mg group, macular edema (1.5% [1/65]) in the ozanimod 0.92 mg group, UC (2.9% [2/68]) in the ozanimod 0.46 mg group, drug-induced liver injury (1.5% [1/65]) in the ozanimod 0.92 mg group, pyrexia (1.5% [1/68]) in the ozanimod 0.46 mg group, alanine aminotransferase increased (3.1% [2/65]) in the ozanimod 0.92 mg group, and aspartate aminotransferase increased (3.1% [2/65]) in the ozanimod 0.92 mg group. The overall incidences of nonserious infections were higher in the placebo group than in the ozanimod groups in the induction period and similar between groups in the induction and maintenance periods. Only 1 serious infection occurred in the induction and maintenance periods: a patient with COVID-19 in the ozanimod 0.92 mg group. There were no deaths during the induction and maintenance periods (Table 2). TEAEs with incidence >2% are listed in Table A7.

In the induction and maintenance periods, the incidence of adverse events of special interest was 3.1% (2/65) in the placebo group, 4.4% (3/68) in the ozanimod 0.46 group,

Table 2. Safety Findings in the Induction and Maintenance Periods

	IP			IP and MP		
TEAEs	Placebo (n = 65)	Ozanimod 0.46 mg (n = 68)	Ozanimod 0.92 mg (n = 65)	Placebo (n = 65)	Ozanimod 0.46 mg (n = 68)	Ozanimod 0.92 mg (n = 65)
≥1 TEAE	36 (55.4)	40 (58.8)	38 (58.5)	43 (66.2)	51 (75.0)	51 (78.5)
≥1 serious TEAE	2 (3.1)	4 (5.9)	4 (6.2)	2 (3.1)	5 (7.4)	5 (7.7)
≥1 serious TEAE related to treatment	1 (1.5)	1 (1.5)	0	1 (1.5)	1 (1.5)	0
≥1 serious TEAE leading to treatment withdrawal	2 (3.1)	3 (4.4)	3 (4.6)	2 (3.1)	4 (5.9)	4 (6.2)
TEAE with incidence ≥5%	1 (1.5)	1 (1.5)	3 (4.6)	1 (1.5)	1 (1.5)	4 (6.2)
TEAE with incidence ≥5%						
Nasopharyngitis	4 (6.2)	5 (7.4)	5 (7.7)	6 (9.2)	10 (14.7)	9 (13.8)
Pyrexia	2 (3.1)	6 (8.8)	2 (3.1)	3 (4.6)	11 (16.2)	7 (10.8)
Headache	4 (6.2)	4 (5.9)	4 (6.2)	4 (6.2)	8 (11.8)	6 (9.2)
Back pain	5 (7.7)	2 (2.9)	3 (4.6)	5 (7.7)	5 (7.4)	6 (9.2)
COVID-19	2 (3.1)	2 (2.9)	1 (1.5)	3 (4.6)	4 (5.9)	5 (7.7)
Colitis ulcerative	1 (1.5)	4 (5.9)	4 (6.2)	1 (1.5)	4 (5.9)	4 (6.2)
Arthralgia	1 (1.5)	2 (2.9)	3 (4.6)	2 (3.1)	4 (5.9)	4 (6.2)
Abdominal pain	1 (1.5)	2 (2.9)	0	1 (1.5)	4 (5.9)	2 (3.1)
GGT increased	0	1 (1.5)	1 (1.5)	0	5 (7.4)	2 (3.1)
Dental caries	1 (1.5)	3 (4.4)	0	2 (3.1)	5 (7.4)	0
Infection with incidence ≥3%						
Nasopharyngitis	4 (6.2)	5 (7.4)	5 (7.7)	6 (9.2)	10 (14.7)	9 (13.8)
COVID-19	2 (3.1)	2 (2.9)	1 (1.5)	3 (4.6)	4 (5.9)	5 (7.7)[a]
Herpes zoster	1 (1.5)	0	1 (1.5)	1 (1.5)	1 (1.5)	2 (3.1)
Hordeolum	0	0	0	2 (3.1)	1 (1.5)	0
Serious infection	0	0	0	0	0	1 (1.5)
Cancer	0	0	0	0	0	0
AESI[b]	2 (3.1)	1 (1.5)	2 (3.1)	2 (3.1)	3 (4.4)	5 (7.7)
Bradycardia	0	0	0	0	0	0
Herpes zoster	1 (1.5)	0	1 (1.5)	1 (1.5)	1 (1.5)	2 (3.1)
Macular edema	0	0	0	0	0	1 (1.5)
Orthostatic hypotension	0	0	0	0	0	1 (1.5)
Cytomegalovirus colitis	0	1 (1.5)	0	0	1 (1.5)	0
Oral herpes	0	0	0	0	1 (1.5)	0
Drug-induced liver injury[c]	0	0	1 (1.5)	0	0	1 (1.5)
Interstitial lung disease	1 (1.5)	0	0	1 (1.5)	0	0
Laboratory assessments						
ALT[d]						
≥2 × ULN	0	3 (4.5)	3 (4.8)	1 (1.6)	9 (13.6)	5 (7.9)
≥3 × ULN	0	0	2 (3.2)	1 (1.6)	1 (1.5)	2 (3.2)
≥5 × ULN	0	0	1 (1.6)	0	1 (1.5)	1 (1.6)
≥10 × ULN	0	0	1 (1.6)[b]	0	0	1 (1.6)[b]
AST[d]						
≥2 × ULN	0	1 (1.5)	2 (3.2)	0	3 (4.5)	3 (4.8)
≥3 × ULN	0	1 (1.5)	2 (3.2)	0	2 (3.0)	2 (3.2)
≥5 × ULN	0	0	2 (3.2)	0	1 (1.5)	2 (3.2)
≥10 × ULN	0	0	0	0	0	0

AESI, adverse event of special interest; ALT, alanine aminotransferase; AST, aspartate aminotransferase; GGT, gamma-glutamyl transferase; IP, induction period; MP, maintenance period; PML, progressive multifocal leukoencephalopathy; TEAE, treatment-emergent adverse event; ULN, upper limit of normal.

[a]1 COVID-19 adverse event that occurred during the MP required hospitalization.

[b]Bradycardia (defined as events that were symptomatic, had a heart rate <45 beats per minute, or required treatment), heart conduction abnormalities (ie, second-degree and higher atrioventricular block), macular edema, malignancy, serious or opportunistic infection, pulmonary effects, hepatic effects, posterior reversible encephalopathy syndrome, PML, and events associated with orthostatic hypotension (eg, dizziness, lightheadedness, fainting, syncope, seizure) were defined as AESIs.

[c]A patient who had a medical history of hepatic steatosis and hyperlipidemia and concomitantly used rosuvastatin and ezetimibe experienced ALT >10 × ULN. The patient discontinued ozanimod as this event met the discontinuation criteria per protocol but total bilirubin remained within the normal range; the patient did not meet Hy's law criteria, and no clinical symptoms were observed. There was no treatment or intervention for this event, and the event was considered to be related to study treatment.

[d]Percentages were based on the numbers of patients with assessments: placebo = 64, ozanimod 0.46 mg = 66, and ozanimod 0.92 mg = 63.

and 7.7% (5/65) in the ozanimod 0.92 mg group. Orthostatic hypotension occurred in 1 patient in the ozanimod 0.92 mg group, and the event was not serious, was mild in severity, and resolved. Macular edema occurred in 1 patient in the ozanimod 0.92 mg group who discontinued treatment due to the event; the event was not serious, was mild in severity, and resolved. Incidences of herpes zoster infection were low and similar across treatment groups; herpes zoster infection occurred in 1 patient each in the placebo group and ozanimod 0.46 mg group and in 2 patients in the ozanimod 0.92 mg group. All cases of herpes zoster resolved while on study treatment. There was 1 case each of cytomegalovirus colitis and oral herpes, both of which occurred in the ozanimod 0.46 mg group and subsequently resolved. There were no AEs related to pulmonary effects with ozanimod treatment. There were no cases of cancer, bradycardia, or progressive multifocal leukoencephalopathy during the study (Table 2).

Elevated liver transaminase levels were more common in the ozanimod treatment groups than in the placebo group (Table 2). No patients with alanine aminotransferase or aspartate aminotransferase >3 × the upper limit of normal elevation experienced accompanying symptoms suggestive of liver injury. Drug-induced liver injury occurred in 1 patient who received ozanimod 0.92 mg, which led to drug withdrawal, but did not meet the criteria for Hy's law (defined as alanine aminotransferase or aspartate aminotransferase levels ≥3 × the upper limit of normal and a total bilirubin >2 × the upper limit of normal)[30] as the patient had a normal total bilirubin despite an alanine aminotransferase level >10 × the upper limit of normal; this event was considered related to study treatment. No Hy's law cases or serious hepatic AEs were associated with hepatic abnormalities.

ALC reductions <500 cells/μL occurred in 3.1% (2/65), 40.3% (27/67), and 58.5% (38/65) of patients in the induction period. An increase in the number of patients with ALC <500 cells/μL was observed in the ozanimod treatment groups when combining the induction and maintenance period to 3.1% (2/65), 52.2% (35/67), and 67.7% (44/65) of patients with an assessment per visit receiving placebo, ozanimod 0.46 mg, and ozanimod 0.92 mg, respectively. Patients who experienced ALC reduction <200 cells/μL had a recovery time to ALC levels >200 cells/μL after treatment interruption within 2 weeks, while no treatment interruption was required for patients with ALC <500 cells/μL. In the induction period, 6.2% (4/65) of patients in the ozanimod 0.92 mg group and no patients in the other groups experienced ALC reductions <200 cells/μL. All patients interrupted treatment per protocol; 3 of these patients resumed ozanimod treatment after ALC levels increased to >500 cells/μL, and 1 patient who discontinued treatment had ALC levels increase to ≥500 cells/μL after treatment interruption. In the induction and maintenance periods, no patients in the placebo group, 3.0% (2/67) of patients in the ozanimod 0.46 mg group, and 13.8% (9/65) of patients in the ozanimod 0.92 mg group experienced ALC reductions <200 cells/μL. Of the 7 new patients who experienced an ALC reduction <200 cells/μL and interrupted study treatment during the maintenance period, 6 patients resumed treatment after ALC levels increased to >500 cells/μL, and ALC levels increased to ≥500 cells/μL after treatment interruption for the 1 patient who discontinued treatment.

In the induction and maintenance periods, no clinically significant abnormalities were noted in any treatment group for electrocardiograms (Tables A8, A9), vital signs (including heart rate, pulse rate, and blood pressure) (Table A10), or physical findings. No patients receiving ozanimod 0.46 mg or ozanimod 0.92 mg reported a mean supine and standing heart rate <50 bpm throughout the induction period and maintenance period.

Discussion

This phase 2/3 study demonstrated that ozanimod was effective and well tolerated in Japanese patients with moderately to severely active UC. During the induction period, high efficacy rates were observed with ozanimod treatment, and significantly greater proportions of patients who received either ozanimod dose achieved the primary endpoint of clinical response versus placebo at week 12. Similar trends were observed for other endpoints, including clinical remission, endoscopic improvement, and mucosal healing. Notably, ozanimod 0.92 mg achieved higher efficacy rates for all evaluated endpoints than ozanimod 0.46 mg. Efficacy was well maintained during the study, with greater response rates in the ozanimod groups than in the placebo group.

As a phase 2/3 study, 2 ozanimod doses were assessed. Dose-dependent effects were observed for efficacy but not safety. Efficacy results in the maintenance period suggest that ozanimod 0.92 mg is the most effective dose to maintain remission. This is consistent with ozanimod labeling in the United States and other countries, including Japan, where ozanimod 0.92 mg is the recommended maintenance dose.[24,25,29]

The efficacy of ozanimod 0.92 mg in J-True North was consistent with that in the induction period of the global 52-week (10-week induction period and rerandomized 42-week maintenance period) True North study[22]; however, when comparing the trials qualitatively, efficacy outcomes were generally better in J-True North at week 12 than in True North at week 10.[22] In the True North induction period, clinical response was achieved in 25.9% of patients receiving placebo and 47.8% of patients receiving ozanimod 0.92 mg, with a weighted difference of 21.9% compared with a placebo-adjusted difference of 30.1% in the ozanimod 0.92 mg group in J-True North. Similarly, clinical remission was achieved in 6.0% of patients receiving placebo and 18.4% of patients receiving ozanimod 0.92 mg with a weighted difference of 12.4% in True

North compared with a placebo-adjusted difference of 22.4% in J-True North. Direct comparisons of efficacy results during the maintenance period by evaluating differences versus placebo are less meaningful due to the differences in study designs; in True North, clinical responders at week 10 were rerandomized to receive placebo or ozanimod, whereas clinical responders at week 12 continued the same treatment from induction during maintenance in J-True North. Placebo-adjusted differences cannot be compared, but the proportion of patients receiving ozanimod 0.92 mg during maintenance who achieved clinical response (60% [138/230] and 80% [32/40]) and clinical remission (37% [85/230] and 47.5% [19/40]) at week 52 in True North and J-True North, respectively, suggest that greater maintenance efficacy results were observed in J-True North. Differences in efficacy during induction may be highly attributed to the different time durations (12 weeks vs 10 weeks) between the induction periods of J-True North and True North, respectively. However, this may have a smaller influence on the greater outcomes observed in the maintenance period of J-True North. In addition, differences in patient populations may have led to better efficacy results in J-True North. Although there were no major differences in baseline characteristics, such as age or Mayo score, more patients were previously exposed to biologics in True North than in J-True North.

The efficacy of ozanimod 0.92 mg in J-True North was similar to that of the S1P modulator etrasimod in the phase 3 ELEVATE UC trials, with similar rates of placebo-adjusted treatment differences observed for clinical remission, clinical response, and endoscopic improvement at week 12; also, similar maintenance efficacy was observed at week 52 in the J-True North and ELEVATE UC 52 trials.[10] However, head-to-head clinical trials comparing the efficacy of ozanimod versus etrasimod in patients with moderately to severely active UC are needed for direct treatment comparisons.

The safety profile of ozanimod in J-True North was consistent with previous ozanimod studies, with no unexpected safety signals.[20,22,31,32] Certain S1P receptor modulators are associated with bradycardia, which may be due to $S1P_1$ receptor binding in cardiac myocytes.[33] There were no cases of bradycardia in J-True North, likely mitigated by the 7-day dose escalation upon dose initiation.[34] There were no clinically significant electrocardiogram findings. There was 1 case of macular edema in the maintenance period. However, longer observation may be needed to accurately assess risk. In True North, macular edema occurred in 3 patients receiving ozanimod during the induction (cohort 1: 0.2% [1/429] and cohort 2: 0.3% [1/367]) and maintenance (0.4% [1/230]) periods and in 1 patient (0.8%; 0.2/100 patient-years) in the True North OLE.[22,35] Herpes zoster infection occurred in 3 patients (2.3%) receiving ozanimod in J-True North, 8 patients (3 [0.4%] during induction and 5 [2.2%] during maintenance) in True North, and 7 patients (5.3%; 1.7 exposure-adjusted incidence rate per 100 patient-years) in the True North OLE. Unlike JAK inhibitors, which have previously demonstrated an increased risk of herpes zoster infection in Asians,[36–38] our results demonstrate that Asian race did not impact rates of herpes zoster infection with ozanimod treatment in comparison to those observed in True North and its OLE.[22,35] However, it is essential for future studies to collect real-world data on the safety of ozanimod in Asian patients with UC and to compare the findings with the safety of JAK inhibitors reported to date.

Infection and malignancies were not frequently observed with ozanimod even in the context of ALC reductions. This may be because ozanimod mainly reduces the distribution of naive CD4+ T cells and central memory subsets, but not effector CD8+ T cells, and has minimal impact on innate immune cells.[39,40] Accordingly, neutrophil and leukocyte changes were limited in this study. No connection between ALC reduction and infection was observed in J-True North or True North. A post hoc analysis of the phase 3 RADIANCE and SUNBEAM MS trials demonstrated that IgG levels with ozanimod treatment were maintained within normal ranges despite dose-dependent decreases in circulating IgG levels, thus suggesting that ozanimod's impact on lymphocytes does not greatly reduce circulating IgG levels.[41] Similar trends regarding ALC reductions and infection were observed with ozanimod treatment in patients with MS. In a pooled safety analysis of all ozanimod MS studies, 1 (0.5%) patient had an ALC $<0.2 \times 10^9$/L around the onset of a serious infection, and 1 (0.5%) patient had a similar ALC level around the onset of a nonserious opportunistic infection.[42] Notably, ALC reductions in patients with relapsing MS recovered to a normal range ($\geq 1 \times 10^9$/L) with a median time to recovery of 30 and 28 days after treatment discontinuation of ozanimod 0.92 mg and 0.46 mg, respectively, in a post hoc assessment of the off-treatment recovery of ALC.[43]

Although ALC reductions are a concern with ozanimod treatment, the severity of lymphopenia caused by ozanimod differs from NUDT15 gene variant thiopurine-induced leukopenia; ozanimod and thiopurine affect immune cells through different mechanisms, leading to varying degrees of immune modulation.[2,44,45] ALC reduction by S1P receptor modulators is a consequence of limiting lymphocyte egression from secondary lymph organs, which preserves lymphocyte function,[19,46,47] whereas leukopenia caused by thiopurines is a cytotoxic event with DNA damage resulting from irreversible bone marrow suppression.[48] Preserved lymphocyte function with ozanimod treatment may partly explain the lack of association observed between the decrease of ALC and serious or opportunistic infections.

Findings from this study support the efficacy and safety of ozanimod in Japanese patients with moderately to severely active UC and its approval for the UC indication in Japan.[29] Although Japanese inflammatory bowel disease treatment guidelines have not yet incorporated ozanimod positioning into the UC therapeutic armamentarium,[4] the American Gastroenterological Association clinical practice

guidelines for moderate to severe UC have suggested early use of advanced therapy, such as ozanimod, after failure of 5-aminosalicylic acid with or without immunomodulator therapy.[49] This is consistent with the approved ozanimod UC indication in Japan for patients with moderately to severely active UC who have had an inadequate response to conventional therapies,[29] thus providing Japanese clinicians with a better understanding of when to initiate ozanimod in clinical practice. This study is strengthened by its treat-through design, which allowed treatment groups to remain consistent for comparisons with placebo over 52 weeks. Although some Asian patients were included in the phase 3 True North study, this is the first analysis to verify the efficacy and safety of ozanimod in a large number of patients in Asia. A limitation of this trial is the paucity of long-term data, but the OLE phase of this study is ongoing. In addition, ozanimod has demonstrated long-term efficacy and safety for up to approximately 3 years of continuous ozanimod treatment in an interim analysis of the True North OLE.[35] Another limitation of this study is that corticosteroid-free remission results could not be interpreted due to the small number of patients receiving concomitant corticosteroid (placebo, n = 3; ozanimod 0.46 mg, n = 4; ozanimod 0.92 mg, n = 7).

Conclusions

Ozanimod was effective and well tolerated as a once-daily oral therapy in Japanese patients with moderately to severely active UC. Results of this large-scale Japanese clinical trial verified the efficacy and safety of ozanimod in a large number of patients in Asia for the first time. The efficacy and safety profile of ozanimod in J-True North was consistent with the findings of the global phase 3 True North study.

Supplementary Materials

Material associated with this article can be found, in the online version, at https://doi.org/10.1016/j.gastha.2025.100812.

References

1. Rubin DT, Ananthakrishnan AN, Siegel CA, et al. ACG Clinical Guideline: ulcerative colitis in adults. Am J Gastroenterol 2019;114:384–413.
2. Scott FL, Clemons B, Brooks J, et al. Ozanimod (RPC1063) is a potent sphingosine-1-phosphate receptor-1 (S1P1) and receptor-5 (S1P5) agonist with autoimmune disease-modifying activity. Br J Pharmacol 2016;173:1778–1792.
3. Yamazaki M, Chung H, Xu Y, et al. Trends in the prevalence and incidence of ulcerative colitis in Japan and the US. Int J Colorectal Dis 2023;38:135.
4. Nakase H, Uchino M, Shinzaki S, et al. Evidence-based clinical practice guidelines for inflammatory bowel disease 2020. J Gastroenterol 2021;56:489–526.
5. Kissei Pharmaceuticals. "CAROGRA® tablets", approved in Japan for treatment of ulcerative colitis - the world-first orally available $\alpha 4$ integrin antagonist [press release]. Kissei Pharmaceuticals, 2022.
6. Awan H, Fatima U, Eaw R, et al. The efficacy of currently licensed biologics for treatment of ulcerative colitis: a literature review. Cureus 2023;15:e37609.
7. Selinger CP, Parkes GC, Bassi A, et al. A multi-centre audit of excess steroid use in 1176 patients with inflammatory bowel disease. Aliment Pharmacol Ther 2017;46:964–973.
8. Waljee AK, Wiitala WL, Govani S, et al. Corticosteroid use and complications in a US inflammatory bowel disease cohort. PLoS One 2016;11:e0158017.
9. Dubinsky MC, Watanabe K, Molander P, et al. Ulcerative colitis narrative global survey findings: the impact of living with ulcerative colitis-patients' and physicians' view. Inflamm Bowel Dis 2021;27:1747–1755.
10. Sandborn WJ, Vermeire S, Peyrin-Biroulet L, et al. Etrasimod as induction and maintenance therapy for ulcerative colitis (ELEVATE): two randomised, double-blind, placebo-controlled, phase 3 studies. Lancet 2023;401:1159–1171.
11. Bressler B. Is there an optimal sequence of biologic therapies for inflammatory bowel disease? Therap Adv Gastroenterol 2023;16:17562848231159452.
12. Sandborn WJ, Van Assche G, Reinisch W, et al. Adalimumab induces and maintains clinical remission in patients with moderate-to-severe ulcerative colitis. Gastroenterology 2012;142:257–265.
13. Sands BE, Sandborn WJ, Panaccione R, et al. Ustekinumab as induction and maintenance therapy for ulcerative colitis. N Engl J Med 2019;381:1201–1214.
14. Núñez P, Quera R, Yarur AJ. Safety of Janus kinase inhibitors in inflammatory bowel diseases. Drugs 2023;83:299–314.
15. Kayal M, Shah S. Ulcerative colitis: current and emerging treatment strategies. J Clin Med 2019;9:94.
16. Pugliese D, Privitera G, Fiorani M, et al. Targeting IL12/23 in ulcerative colitis: update on the role of ustekinumab. Therap Adv Gastroenterol 2022;15:17562848221102283.
17. Cui G, Fan Q, Li Z, et al. Evaluation of anti-TNF therapeutic response in patients with inflammatory bowel disease: current and novel biomarkers. EBioMedicine 2021;66:103329.
18. Schwartz DM, Kanno Y, Villarino A, et al. JAK inhibition as a therapeutic strategy for immune and inflammatory diseases. Nat Rev Drug Discov 2017;16:843–862.
19. Bencardino S, D'Amico F, Faggiani I, et al. Efficacy and safety of S1P1 receptor modulator drugs for patients with moderate-to-severe ulcerative colitis. J Clin Med 2023;12:5014.
20. Sandborn WJ, Feagan BG, Wolf DC, et al. Ozanimod induction and maintenance treatment for ulcerative colitis. N Engl J Med 2016;374:1754–1762.
21. Pérez-Jeldres T, Alvarez-Lobos M, Rivera-Nieves J. Targeting sphingosine-1-phosphate signaling in immune-mediated diseases: beyond multiple sclerosis. Drugs 2021;81:985–1002.

22. Sandborn WJ, Feagan BG, D'Haens G, et al. Ozanimod as induction and maintenance therapy for ulcerative colitis. N Engl J Med 2021;385:1280–1291.
23. Bristol Myers S. U.S. Food and Drug Administration approves Bristol Myers Squibb's ZEPOSIA® (ozanimod), a new oral treatment for relapsing forms of multiple sclerosis [press release]. Princeton, NJ: Bristol Myers Squibb, 2020.
24. Zeposia [package insert]. Princeton, NJ: Bristol Myers Squibb, 2024.
25. Zeposia [summary of product characteristics]. Utrecht, Netherlands: Celgene Distribution B.V., 2024.
26. Choi D, Stewart AP, Bhat S. Ozanimod: a first-in-class sphingosine 1-phosphate receptor modulator for the treatment of ulcerative colitis. Ann Pharmacother 2022;56:592–599.
27. Bristol Myers S. Bristol Myers Squibb receives European Commission approval of Zeposia (ozanimod) for use in adults with moderately to severely active ulcerative colitis. Princeton, NJ: Bristol Myers Squibb, 2021.
28. Bristol Myers S. Bristol Myers Squibb receives European Commission approval for Zeposia (ozanimod) for the treatment of adult patients with relapsing remitting multiple sclerosis with active disease [press release]. Bristol Myers Squibb, 2020.
29. Zeposia Japanese [package insert]. Tokyo, Japan: Bristol Myers Squibb, 2024.
30. Guidance for Industry: Drug-induced liver injury: premarketing clinical evaluation. Silver Spring, MD: U.S. Department of Health and Human Services, Food and Drug Administration, 2009.
31. Cohen JA, Comi G, Selmaj KW, et al. Safety and efficacy of ozanimod versus interferon beta-1a in relapsing multiple sclerosis (RADIANCE): a multicentre, randomised, 24-month, phase 3 trial. Lancet Neurol 2019;18:1021–1033.
32. Comi G, Kappos L, Selmaj KW, et al. Safety and efficacy of ozanimod versus interferon beta-1a in relapsing multiple sclerosis (SUNBEAM): a multicentre, randomised, minimum 12-month, phase 3 trial. Lancet Neurol 2019;18:1009–1020.
33. Chun J, Giovannoni G, Hunter SF. Sphingosine 1-phosphate receptor modulator therapy for multiple sclerosis: differential downstream receptor signalling and clinical profile effects. Drugs 2021;81:207–231.
34. Tran JQ, Hartung JP, Peach RJ, et al. Results from the first-in-human study with ozanimod, a novel, selective sphingosine-1-phosphate receptor modulator. J Clin Pharmacol 2017;57:988–996.
35. Danese S, Panaccione R, Abreu MT, et al. Efficacy and safety of approximately 3 years of continuous ozanimod in moderately to severely active ulcerative colitis: interim analysis of the True North open-label extension. J Crohns Colitis 2024;18:264–274.
36. Winthrop KL, Melmed GY, Vermeire S, et al. Herpes zoster infection in patients with ulcerative colitis receiving tofacitinib. Inflamm Bowel Dis 2018;24:2258–2265.
37. Yamanaka H, Tanaka Y, Takeuchi T, et al. Tofacitinib, an oral Janus kinase inhibitor, as monotherapy or with background methotrexate, in Japanese patients with rheumatoid arthritis: an open-label, long-term extension study. Arthritis Res Ther 2016;18:34.
38. Gialouri CG, Moustafa S, Thomas K, et al. Herpes zoster in patients with inflammatory arthritides or ulcerative colitis treated with tofacitinib, baricitinib or upadacitinib: a systematic review of clinical trials and real-world studies. Rheumatol Int 2023;43:421–435.
39. Harris S, Tran JQ, Southworth H, et al. Effect of the sphingosine-1-phosphate receptor modulator ozanimod on leukocyte subtypes in relapsing MS. Neurol Neuroimmunol Neuroinflamm 2020;7:e839.
40. Harris S, Feagan BG, Hanauer S, et al. Ozanimod differentially impacts circulating lymphocyte subsets in patients with moderately to severely active Crohn's disease. Dig Dis Sci 2024;69:2044–2054.
41. Harris S, Southworth H, Sheffield JK, et al. A post hoc analysis of immunoglobulin levels in patients with relapsing multiple sclerosis treated with ozanimod in phase 3 trials [abstract P159]. Mult Scler J 2022;28(1 suppl):93–94.
42. Selmaj KW, Cohen JA, Comi G, et al. Ozanimod in relapsing multiple sclerosis: pooled safety results from the clinical development program. Mult Scler Relat Disord 2021;51:102844.
43. Zeposia assessment report. European Medicines Agency, 2020.
44. Matsuoka K. NUDT15 gene variants and thiopurine-induced leukopenia in patients with inflammatory bowel disease. Intest Res 2020;18:275–281.
45. Bayoumy AB, Ansari AR, Mulder CJJ, et al. Innovating thiopurine therapeutic drug monitoring: a systematic review and meta-analysis on DNA-thioguanine nucleotides (DNA-TG) as an inclusive biomarker in thiopurine therapy. Clin Pharmacokinet 2024;63:1089–1109.
46. Chiba K, Yanagawa Y, Masubuchi Y, et al. FTY720, a novel immunosuppressant, induces sequestration of circulating mature lymphocytes by acceleration of lymphocyte homing in rats. I. FTY720 selectively decreases the number of circulating mature lymphocytes by acceleration of lymphocyte homing. J Immunol 1998;160:5037–5044.
47. Pinschewer DD, Ochsenbein AF, Odermatt B, et al. FTY720 immunosuppression impairs effector T cell peripheral homing without affecting induction, expansion, and memory. J Immunol 2000;164:5761–5770.
48. Yamashita N, Kawahara M, Imai T, et al. Loss of Nudt15 thiopurine detoxification increases direct DNA damage in hematopoietic stem cells. Sci Rep 2023;13:11908.
49. Singh S, Loftus EV Jr, Limketkai BN, et al. AGA living clinical practice guideline on pharmacological management of moderate-to-severe ulcerative colitis. Gastroenterology 2024;167:1307–1343.

Received May 29, 2025. Accepted September 10, 2025.

Correspondence:
Address correspondence to: Hiroshi Nakase, Sapporo Medical University, Minami 1-jo Nishi 16-chome, Chuo Ward Sapporo, Sapporo, Hokkaido 060-8556, Japan. e-mail: hiropynakase@gmail.com.

Acknowledgments:
Third-party writing assistance for this manuscript was provided by Rebecca Lane, PhD, Julie Ko, PharmD, and Anny Wu, PharmD, of Peloton Advantage, LLC, an OPEN Health company.

Authors' Contributions:
Conception or design: Hiroshi Nakase, Tadakazu Hisamatsu, Yasuo Suzuki, Mamoru Watanabe, Yoko Uchikawa, Shoichiro Goto, Go Fujimoto, Changliang Zhang, AnnKatrin Petersen, Toshifumi Hibi.
Data acquisition: Hiroshi Nakase, Toshimitsu Fujii, Tadakazu Hisamatsu, Sakuma Takahashi, Makoto Ooi, Ken Takeuchi, Tsuguhiro Kimura, Ken Furuya, Nobuo Aoyama, Kenkei Hasatani, Noriyuki Horiki, Kazunari Kanke, Satoki Tokito, Souken Sai, Toshifumi Hibi.
Data analysis: Go Fujimoto.
Data interpretation: Hiroshi Nakase, Yoko Uchikawa, Shoichiro Goto, Go Fujimoto, Changliang Zhang.
Writing – review & editing: All authors.

Conflicts of Interest:
Hiroshi Nakase received personal fees from AbbVie, Daiichi Sankyo, EA Pharma, Gilead Sciences, Janssen Pharmaceutical K. K., JIMRO, Kissei Pharmaceutical, KYORIN Pharmaceutical, Mitsubishi Tanabe Pharma, Mochida Pharmaceutical, Pfizer Japan, Takeda Pharmaceutical, and Viatris; received grants for commissioned/joint research from AbbVie, Hoya Group Pentax Medical, Mitsubishi Tanabe Pharma, and Mochida Pharmaceutical; and has an endowed chair funded by JIMRO, KYORIN Pharmaceutical, Miyarisan Pharmaceutical, and Mochida Pharmaceutical. Toshimitsu Fujii received research grants from AbbVie GK, Alfresa, Boehringer Ingelheim, Bristol Myers Squibb, Celltrion, EA Pharma, Eli Lilly, Gilead Sciences, Janssen, Kissei, Mebix, Sanofi, and Takeda; received speaker fees from AbbVie GK, Boehringer Ingelheim, Bristol Myers Squibb, EA Pharma, Janssen, Kissei, KYORIN, Mitsubishi Tanabe, Mochida, Nichi-Iko Pharmaceutical, Nippon Kayaku, Pfizer, Taiho Pharma, Takeda, and Zeria. Tadakazu Hisamatsu received grant support from AbbVie GK, Abivax, Boston Scientific, Bristol Myers Squibb, EA Pharma, JIMRO, KYORIN, Mitsubishi Tanabe, Mochida, Nippon Kayaku, Pfizer, Takeda, and Zeria; consulted for AbbVie GK, EA Pharma, Eli Lilly, Gilead Sciences, Janssen, Mitsubishi Tanabe, and Pfizer; received lecture fees from AbbVie GK, EA Pharma, Janssen, JIMRO, Kissei, KYORIN, Mitsubishi Tanabe, Mochida, Pfizer, and Takeda. Yasuo Suzuki received speaker fees from AbbVie GK, Janssen, Mitsubishi Tanabe, and Takeda. Mamoru Watanabe received research support from AbbVie GK, EA Pharma, Kissei, Mitsubishi Tanabe, Nippon Kayaku, Takeda, and Zeria; received speaker fees from Gilead Sciences. Sakuma Takahashi received speaker fees from AbbVie GK, EA Pharma, Janssen Pharmaceutical K. K., KYORIN Pharmaceutical, Mitsubishi Tanabe, Mochida, and Takeda. Makoto Ooi received lecture fees from AbbVie GK, Bristol Myers Squibb, EA Pharma, Janssen, JIMRO, Kissei, KYORIN, Mitsubishi Tanabe Pharma, Mochida Pharmaceutical, Nippon Kayaku, ONO Pharma, Pfizer, and Takeda. Ken Takeuchi received grants/contracts from AbbVie GK, Amgen K. K., AstraZeneca K. K., Bristol-Myers Squibb K. K., EA Pharma Co., Ltd, Eli Lilly Japan K. K., Ferring Pharmaceutical Co., Ltd., IQVIA Inc, Nippon Shinyaku Co., Ltd., and Takeda Pharmaceutical Company Limited; consulting fees from Thermo Fisher Diagnostics K. K.; payments/honoraria from AbbVie GK, Ayumi Pharmaceutical Corporation, Celltrion Healthcare, EA Pharma Co., Ltd, Gilead Sciences, Inc, Janssen Pharmaceutical K. K., JIMRO Co., Ltd., Kissei Pharmaceutical Co., Ltd, Kyorin Pharmaceutical Co., Ltd., Mitsubishi-Tanabe Pharma Corporation, Mochida Pharmaceutical Co., Ltd., Otsuka Holdings Co., Ltd, Pfizer Japan Inc., Takeda Pharmaceutical Company Limited, Viatris Inc., and Zeria Pharmaceutical Co., Ltd. Yoko Uchikawa, Shoichiro Goto, Go Fujimoto, Changliang Zhang, and AnnKatrin Petersen are employees and/or shareholders of Bristol Myers Squibb. Toshifumi Hibi received lecture fees from AbbVie GK, Janssen, JIMRO, Mitsubishi Tanabe, Mochida, Pfizer, Sand K. K., Takeda, and Zeria; received advisory/consultancy fees from AbbVie GK, Celltrion, EA Pharma, Eli Lilly, Gilead Sciences, Mitsubishi Tanabe, Takeda, and Zeria; received research grants from AbbVie GK, ActivAid, Alfresa, Bristol Myers Squibb, Eli Lilly Japan K. K., Ferring, Gilead Sciences, Janssen Pharmaceutical K. K., JMDC, Mochida, Nippon Kayaku, Pfizer Japan, and Takeda; belonged to study group sponsorship for Alfresa, JIMRO, Kyorin, Mochida, Miyarisan, and Zeria. The remaining authors disclose no conflicts.

Funding:
This work was supported by Bristol Myers Squibb, Princeton, NJ, USA.

Ethics Statement:
J-True North adhered to the Good Clinical Practices guidelines and was conducted in accordance with the ethical principles outlined in the Declaration of Helsinki. Before study initiation, the study protocol and informed consent were approved at each study site by an institutional review board or independent ethics committee. Written informed consent was obtained from each patient prior to entering the study and before the initiation of any trial-related procedure. J-True North was sponsored by Bristol Myers Squibb.

Data Transparency Statement:
Bristol Myers Squibb policy on data sharing may be found at https://www.bms.com/researchers-and-partners/independent-research/data-sharing-request-process.html. Deidentified individual patient data will not be shared.

Reporting Guidelines:
CONSORT.

Gastro Hep Advances, Volume *5*

Supplemental information

Once-Daily Oral Ozanimod for Japanese Patients With Ulcerative Colitis: Results From the Phase 2/3 J-True North Study

Hiroshi Nakase, Toshimitsu Fujii, Tadakazu Hisamatsu, Yasuo Suzuki, Mamoru Watanabe, Sakuma Takahashi, Makoto Ooi, Ken Takeuchi, Tsuguhiro Kimura, Ken Furuya, Nobuo Aoyama, Kenkei Hasatani, Noriyuki Horiki, Kazunari Kanke, Satoki Tokito, Souken Sai, Yoko Uchikawa, Shoichiro Goto, Go Fujimoto, Changliang Zhang, AnnKatrin Petersen, and Toshifumi Hibi

SUPPLEMENTARY MATERIAL

Supplementary Methods

Inclusion Criteria

1. Japanese adults aged 18 to 75 years at the time of signing the informed consent form at screening.
2. Patient has had ulcerative colitis (UC) diagnosed ≥3 months prior to first investigational product administration. The diagnosis should be confirmed by clinical and endoscopic evidence and corroborated by a histopathology report (endoscopy and histopathology may be performed at screening if no prior report is readily available).
3. Patient has evidence of UC extending ≥15 cm from the anal verge as determined by baseline endoscopy (flexible sigmoidoscopy or colonoscopy).
4. Patient has active UC defined as Mayo score of 6–12 inclusive, with Mayo endoscopy subscore of ≥2, a rectal bleeding subscore of ≥1, and a stool frequency subscore ≥1.
5. Patient must have been treated with aminosalicylates or corticosteroids. If patients are currently receiving treatment with ≥1 of the following therapies, the patient must continue on these therapies during Induction:
    a. Oral aminosalicylates at a therapeutic dose for their disease (eg, mesalazine, sulfasalazine), with the dose stable for ≥2 weeks prior to screening endoscopy.
    b. Prednisolone (doses ≤10 mg/day) or equivalent receiving a stable dose for ≥2 weeks prior to screening endoscopy.
6. Patient has undergone colonoscopy (or is willing to undergo colonoscopy during screening):
    a. Within the past 2 years, to screen for dysplasia (unless otherwise recommended by local and national guidelines) if the patient has had left-sided colitis for >12 years or total/extensive colitis for >8 years.
    b. Within the past 5 years, to screen for polyps if the patient age is >45 years.

7. If oral aminosalicylates or corticosteroids have been recently discontinued, patient must have been stopped for ≥2 weeks prior to the endoscopy used for baseline Mayo score.
8. Females of childbearing potential:
    a. Patient must agree to practice a highly effective method of contraception throughout the study until completion of the 90-day safety follow-up visit. Highly effective methods of contraception are those that alone or in combination result in a failure rate of a Pearl Index of <1% per year when used consistently and correctly. The following are acceptable methods of birth control in the study:
        i. Combined hormonal (containing estrogen and progestogen) contraception, which may be oral, intravaginal, or transdermal
        ii. Progestogen-only hormonal contraception associated with inhibition of ovulation, which may be oral, injectable, or implantable
        iii. Placement of an intrauterine device
        iv. Placement of a hormone-releasing intrauterine system
        v. Bilateral tubal occlusion
        vi. Vasectomized partner
        vii. Complete sexual abstinence
    b. A female of childbearing potential is a sexually mature female who (1) has not undergone a hysterectomy (the surgical removal of the uterus) or bilateral oophorectomy (the surgical removal of both ovaries) or (2) has not been postmenopausal for ≥24 consecutive months (ie, has had menses at any time during the preceding 24 consecutive months). Periodic abstinence (calendar, symptothermal, postovulation methods), withdrawal (coitus interruptus), spermicides only, and the lactational amenorrhea method are not acceptable methods of contraception.
9. Patient provides written informed consent and confirms compliance with the schedule of protocol assessments.

10. Patients must have documentation of positive varicella zoster virus immunoglobulin G antibody status or complete varicella zoster virus vaccination ≥30 days prior to randomization.

Exclusion Criteria

Exclusions Related to General Health

1. Patient has severe extensive colitis as evidenced by:
    a. Physician judgment that the patient is likely to require colectomy or ileostomy within 12 weeks of baseline.
    b. Current or recent (within 3 months of screening) evidence of fulminant colitis, toxic megacolon, or bowel perforation.
2. Patient has a diagnosis of Crohn’s disease, indeterminate colitis, the presence or history of a fistula consistent with Crohn’s disease, microscopic colitis, radiation colitis, or ischemic colitis.
3. Patient has a positive stool examination for pathogens (ova and parasites, bacteria) or a positive test for toxin producing Clostridioides difficile (C. difficile) at screening. Polymerase chain reaction examination of the stool for C. difficile may be used to exclude false positives. If positive, patients may be treated and retested. Documentation of a negative test result for pathogens (ova and parasites, bacteria) is required within 60 days of day 1.
4. Patient is pregnant or breastfeeding, or has a positive serum beta-human chorionic gonadotropin measured during screening.
5. Patient has clinically relevant hepatic, neurological, pulmonary (severe respiratory disease, pulmonary fibrosis and chronic obstructive pulmonary disease), ophthalmological, endocrine, psychiatric, or other major systemic disease making implementation of the protocol or interpretation of the study difficult or that would put the patient at risk by participating in the study.
6. Patient has clinically relevant cardiovascular conditions, including history or presence of:
    a. Recent (within the last 6 months of screening) occurrence of myocardial infarction, unstable angina, stroke, transient ischemic attack, symptomatic

bradycardia, decompensated heart failure requiring hospitalization, class III/IV heart failure, or severe untreated sleep apnea.

b. Second degree (Mobitz type II) atrioventricular block, third-degree atrioventricular block, sick sinus syndrome, or sinoatrial block in patients without a pacemaker in place; if second-degree Type II or third-degree atrioventricular block is due to concomitant medication, consult the medical monitor prior to screening.

c. Prolonged QT interval corrected for heart rate using Fridericia's formula (QTcF; QTcF >450 msec males, >470 msec females) at either screening or day 1 predose assessment. One recheck is allowed for patients per visit (ie, during the screening and/or day 1 predose assessment visit).

d. Resting heart rate <55 beats per minute when taking vital signs as part of the physical examination at either screening or day 1 predose assessment. One recheck is allowed for patients with heart rate <55 beats per minute per visit (ie, during the screening and/or day 1 predose assessment visit).

e. Patients with the preexisting cardiac conditions listed below must be seen by a consulting cardiologist and cleared to participate in the study without more intensive monitoring. These patients will also follow first-dose monitoring procedures.

    i. History (>6 months prior to screening) of ischemic heart disease, cardiac arrest, cerebrovascular disease, uncontrolled hypertension, history of recurrent syncope, or symptomatic bradycardia

    ii. Second-degree (Mobitz type II) atrioventricular block, third-degree atrioventricular block, sick sinus syndrome, or sinoatrial block with functional pacemaker

    iii. Patients on medicinal products that may potentiate bradycardia (other than the combination of beta-blockers and calcium channel blockers)

7. Patient has a history of diabetes mellitus type 1 or uncontrolled diabetes mellitus type 2 with glycosylated hemoglobin >9%; or diabetic patients with significant

comorbid conditions (eg, retinopathy, nephropathy).

8. Patient has a history of uveitis (within the last year) or a history of macular edema.
9. Patient has a known active bacterial, viral, or fungal infection (excluding fungal infection of nail beds, minor upper respiratory tract infections, and minor skin infections), a mycobacterial infection (including tuberculosis or atypical mycobacterial disease), or any major episode of infection that either required hospitalization or treatment with intravenous antibiotics within 30 days of screening, or treatment with oral antibiotics within 14 days of screening.
    a. In the case of a known SARS-CoV-2 infection, symptoms must have completely resolved; based on investigator assessment in consultation with the clinical trial physician/medical monitor, there are no sequelae that would place the patient at a higher risk of receiving investigational treatment.
10. Patient has a history or known presence of recurrent or chronic infection (eg, hepatitis A, B, or C; HIV); recurrent urinary tract infections are allowed.
11. Patient has a history of cancer, including solid tumors and hematological malignancies (except basal cell and in situ squamous cell carcinomas of the skin or uterine cervix that have been excised and resolved), or colonic mucosal dysplasia.
12. Patient has a history of alcohol or drug abuse within 1 year prior to randomization.
13. Patient has a history of or currently active primary or secondary immunodeficiency.

Exclusions Related to Medications

1. Patient has a history of treatment with a biologic agent within 8 weeks or 5 elimination half-lives (whichever is less) of that agent prior to randomization.
2. Patient has a history of treatment with tofacitinib within 5 elimination half-lives of that agent prior to randomization.
3. Patient has a history of treatment with an investigational agent within 5 elimination half-lives of that agent prior to randomization.

4. Patient has a history of treatment with topical rectal 5-aminosalicylic acid or topical rectal steroids within 2 weeks of screening endoscopy or antimotility medications (such as diphenoxylate/atropine) during screening.
5. Patient has received a live vaccine or live attenuated vaccine within 4 weeks prior to randomization.
6. Patient has been treated previously with lymphocyte-depleting therapies (eg, alemtuzumab, anti-CD4, cladribine, rituximab, cyclophosphamide, mitoxantrone, total body irradiation, bone marrow transplantation).
7. Patient has been treated with cyclosporine, tacrolimus, sirolimus, or mycophenolate mofetil within 16 weeks of screening. If no renal disorder or cutaneous malignancy is confirmed, the washout period of tacrolimus can be reduced to 8 weeks. To check renal disorder, serum creatinine/glomerular filtration rate, albumin-to-creatinine ratio, and serum potassium should be measured.
8. Patient has been treated previously with D-penicillamine, leflunomide, or thalidomide.
9. Patient has been treated previously with natalizumab, fingolimod, or other sphingosine 1-phosphate receptor modulators.
10. Patient has a history of treatment with intravenous immunoglobulin or plasmapheresis within 3 months prior to randomization.
11. Patient has planned concurrent treatment with antineoplastic immunosuppressive agents (ie, azathioprine or methotrexate) after randomization. Patients receiving azathioprine or methotrexate at screening must discontinue treatment with these agents 4 weeks prior to randomization.
12. Patient is treated with chronic nonsteroidal antiinflammatory drugs (occasional use of nonsteroidal antiinflammatory drugs and acetaminophen [for headache, arthritis, myalgias, or menstrual cramps] and aspirin up to 325 mg/day is permitted).
13. Patient is treated with class Ia or class III antiarrhythmic drugs or with ≥2 agents in a combination known to prolong PR interval, or treatment with additional prohibited systemic cardiac medications.

14. Patient has been treated with apheresis within 2 weeks of randomization.
15. Patients who were primary nonresponders to ≥2 biologic agents approved for the treatment of UC (ie, anti–tumor necrosis factor agents or vedolizumab).
16. Patient is receiving treatment with breast cancer resistance protein inhibitors (eg, cyclosporine, eltrombopag).
17. Patient is receiving treatment with any of the following drugs or interventions within the corresponding timeframe:
    a. At randomization
        i. CYP2C8 inhibitors (eg, clopidogrel) or inducers (eg, rifampicin)
    b. 2 weeks prior to randomization
        i. Monoamine oxidase inhibitors (eg, selegiline).

Exclusions Related to Laboratory Results and Other Assessments

18. Patient had the following laboratory results:
    a. Serum creatinine >1.4 mg/dL for females or >1.6 mg/dL for males
    b. Liver function impairment or persisting elevations of aspartate aminotransferase or alanine aminotransferase >2 × the upper limit of normal (ULN) or direct bilirubin >1.5 × ULN
    c. Platelet count <100,000/µL
    d. Hemoglobin <8.0 g/dL
    e. Neutrophils <1500 /µL
    f. Absolute white blood cell count <3500/µL
    g. Absolute lymphocyte count <800/µL
    h. ECG showing any clinically significant abnormality

Study Design

Open-label extension (OLE)

Patients who experienced disease relapse during the maintenance period were eligible to enter the OLE if they met all of the following criteria: increase in UC disease activity as defined by an increase in partial Mayo score of ≥2 points compared with the week 12 partial Mayo score with an absolute partial Mayo score ≥4 points, Mayo endoscopy subscore ≥2 points, and exclusion of other causes of an increase in disease activity

unrelated to underlying UC (eg, infections, change in medication). Patients withdrew from the OLE if they did not achieve clinical improvement after 12 weeks of ozanimod treatment in the OLE. The OLE will continue until marketing launch or until the sponsor discontinues the development program.

Supplementary Table 1. List of Japan-True North Study Principal Investigators, Sites, and Institutional or Ethical Review Boards

Site No.	Principal investigator name	Name/address of EIC/IRB
101	Kawaratani, Hideto	Nara Medical University Hospital
102	Matsuoka, Katsuyoshi	Toho University Medical Center Sakura Hospital
103	Motoya, Satoshi	Sapporo-Kosei General Hospital
104	Ishigami, Keisuke	Sapporo Medical University Hospital
105	Watanabe, Kenji	Hyogo College of Medicine Hospital
106	Oka, Shiro	Hiroshima University Hospital
107	Nakano, Masaru	Kitasato University Kitasato Institute Hospital
108	Saruta, Masayuki	Jikei University Hospital
109	Hisamatsu, Tadakazu	Kyorin University Hospital
110	Matsumoto, Takayuki	Iwate Medical University Uchimaru Medical Center
111	Takedatsu, Hidetoshi	Kurume University Hospital
112	Inaba, Tomoki	Kagawa Prefectural Central Hospital
113	Bamba, Shigeki	Shiga University of Medical Science Hospital
114	Hasatani, Kenkei	Fukui Prefectural Hospital
115	Kimura, Tsuguhiro	Medical Corporation Shoyu-Kai Fujita Gastroenterology Hospital
116	Tokito, Satoki	Tokitokai Tokito Clinic
117	Fukata, Masayuki	Tokyo Yamate Medical Center
118	Ninomiya, Tomoyuki	Ehime Prefectural Central Hospital
119	Hisanaga, Yasuhiro	Ogaki Municipal Hospital
120	Ito, Toru	Kanazawa Medical University Hospital
121	Matano, Yutaka	Komatsu Municipal Hospital
122	Hisabe, Takashi	Fukuoka University Chikushi Hospital
123	Horiki, Noriyuki	Mie University hospital
124	Munemoto, Yoshinori	Fukui-ken Saiseikai Hospital
125	Takamura, Masaaki	Nagaoka Chuo General Hospital
126	Kamoshida, Toshiro	Hitachi General Hospital
127	Sato, Yuichiro	Osaki Citizen Hospital
128	Minato, Yohei	NTT Medical Center Tokyo
129	Kamiyamamoto, Shinji	Toyama City Hospital
130	Kodama, Yuzo	Kobe University Hospital
131	Imaeda, Hiroyuki	Saitama Medical University Hospital
132	Hosomi, Shuhei	Osaka Metropolitan University
133	Sakuraba, Hirotake	Hirosaki University Hospital
134	Matsushima, Masashi	Tokai University Hospital
135	Nakatsu, Morihito	Mitoyo General Hospital
136	Yoshioka, Masao	Okayama Saiseikai Outpatient Center Hospital
137	Tanaka, Toshio	Shizuoka City Shizuoka Hospital
138	Fujii, Toshimitsu	Institute of Science Tokyo
139	Arai, Takehiro	Tokatsu Tsujinaka Hospital
140	Yamazaki, Kenji	Gifu Prefectural General Medical Center
141	Naito, Yuji	University Hospital Kyoto Prefectural University of Medicine
142	Kawano, Hiroshi	Our Lady of the Snow Social Medical Corporation St. Mary's Hospital
143	Kanda, Naoki	Takatsuki Red Cross Hospital
144	Ishino, Atsushi	Hoshi General Hospital
145	Hiramatsu, Naoki	Osaka Rosai Hospital

146	Kumagai, Shinji	IMS Meirikai Sendai General Hospital
147	Furuya, Ken	Japan Community Health Care Organization Hokkaido Hospital
148	Kanke, Kazunari	Kanke Gastrointestinal Clinic
149	Ohnishi, Yoshifumi	National Hospital Organization Shizuoka Medical Center
150	Sai, Souken	Sai Gastroenterology and Proctology Clinic
151	Kobayashi, Toshihisa	Hakodate Goryoukaku Hospital
152	Kuroda, Tsuyoshi	Mazda Hospital of Mazda Motor Corporation
153	Fukuchi, Takumi	Iseikai Hospital
154	Sakata, Yasuhisa	Saga University Hospital
155	Aoyagi, Kunihiko	Japanese Red Cross Fukuoka Hospital
156	Hiraoka, Sakiko	Okayama University Hospital
157	Kato, Shingo	Saitama Medical Center, Saitama Medical University
158	Takeuchi, Ken	Tsujinaka Hospital Kashiwanoha
159	Ishida, Tetsuya	Ishida Clinic of IBD and Gastroenterology
160	Watanabe, Chiyuki	Hiroshima Prefectural Hospital
161	Ochiai, Toshiaki	Saiseikai Fukuoka General Hospital
162	Kubokawa, Masaru	Aso Iizuka Hospital
163	Aoyama, Nobuo	Aoyama Clinic GI Endoscopy and IBD Center
164	Haraguchi, Kazuhiro	Hara Sanshin Hospital
165	Hidaka, Hisamitsu	Hidaka Coloproctology Clinic
166	Tobita, Kouji	Hiratsuka Gastroenterological Hospital
167	Yamamura, Takeshi	Nagoya University Hospital

EIC, ethical review board; IRB, institutional review board.

Supplementary Table 2. Definitions of Efficacy Endpoints

**Efficacy endpoints**	**Definition**
Clinical response	• Complete Mayo score definition: a reduction from baseline in the complete Mayo score of ≥3 points and ≥30% and a reduction from baseline in the RBS of ≥1 point or an absolute RBS of ≤1 point<br>− Complete Mayo score: sum of SFS, RBS, Mayo endoscopy subscore, Physician Global Assessment (each assessment rated from 0–3)<br>• 9-point Mayo score definition: reduction from baseline in the 9-point Mayo score of ≥2 points and ≥35%, and a reduction from baseline in the RBS of ≥1 point or an absolute RBS of ≤1 point<br>− 9-point Mayo score: sum of RBS, SFS, and Mayo endoscopy subscore (each assessment rated from 0–3)
Clinical remission	• Definition 1: complete Mayo score of ≤2 points with no individual subscore ≥1 point<br>• Definition 2: RBS = 0 and SFS ≤1 (and a decrease of ≥1 point from baseline SFS) and Mayo endoscopy subscore ≤1<br>• Definition 3: SFS = 0 or 1 (without a requirement of a decrease of ≥1 from baseline SFS), RBS = 0, endoscopy subscore = 0 or 1

Endoscopic improvement	• Endoscopy subscore ≤1
Mucosal healing	• Endoscopy subscore ≤1 with a Geboes score <2.0
Histologic remission	• Geboes score <2.0

RBS, rectal bleeding subscore; SFS, stool frequency subscore.

Supplementary Table 3. Change From Baseline in FCP and CRP Levels at Weeks 12 and 52

	Induction (Week 12)			Maintenance (Week 52)		
	**Placebo**	**Ozanimod 0.46 mg**	**Ozanimod 0.92 mg**	**Placebo**	**Ozanimod 0.46 mg**	**Ozanimod 0.92 mg**
**FCP, μg/g**						
n	65	68	65	65	68	65
Mean (SD) at baseline	2168.6 (3173.2)	2649.0 (3532.7)	2784.4 (4418.4)	2168.6 (3173.2)	2649.0 (3532.7)	2784.4 (4418.4)
Median (range) at baseline	1060.0 (12.7, 16,800.0)	1500.0 (26.6, 15,200.0)	885.0 (19.6, 22,200.0)	1060.0 (12.7, 16,800.0)	1500.0 (26.6, 15,200.0)	885.0 (19.6, 22,200.0)
n	59	59	59	12	34	35
Mean (SD) change from baseline	−230.1 (4248.0)	−1693.2 (3674.3)	−1581.0 (5379.3)	−1445.4 (1504.7)	−2015.9 (3426.2)	−2449.1 (6344.0)
Median (range) change from baseline	−28.4 (−16,781.7, 10,340.0)	−567.0 (−15,190.0, 7671.5)	−494.2 (−21,340.0, 17,100.0)	−986.0 (−3961.0, 495.3)	−1168.5 (−15,190.0, 6963.0)	−638.3 (−22,185.2, 17,608.0)
**CRP, mg/L**						
n	65	68	65	65	68	65
Mean (SD) at baseline	3.7 (7.6)	4.5 (8.4)	3.5 (6.4)	3.7 (7.6)	4.5 (8.4)	3.5 (6.4)
Median (range) at baseline	1.4 (0.1, 49.7)	1.7 (0.1, 54.2)	1.6 (0.1, 42.7)	1.4 (0.1, 49.7)	1.7 (0.1, 54.2)	1.6 (0.1, 42.7)
n	59	59	59	12	34	35
Mean (SD) change from baseline	−0.5 (5.5)	−2.3 (7.9)	−1.2 (7.2)	−0.4 (6.1)	−1.4 (5.8)	−1.5 (3.6)
Median (range) change from baseline	−0.0 (−25.5, 15.6)	−0.7 (−52.3, 2.3)	−0.2 (−30.8, 35.1)	−0.6 (−8.7, 17.0)	−0.6 (−28.4, 9.5)	−0.5 (−14.7, 4.4)

CRP, C-reactive protein; FCP, fecal calprotectin; SD, standard deviation.

Supplementary Table 4. FCP Response at Week 12

FCP, μg/g	Placebo (N=65)	Ozanimod 0.46 mg (N=68)	Ozanimod 0.92 mg (N=65)
>50 to ≤50 μg/g			
Number of patients with change in FCP levels from baseline, n/N (%)	3/55 (5.5)	19/57 (33.3)	13/57 (22.8)
>100 to ≤100 μg/g			
Number of patients with change in FCP levels from baseline, n/N (%)	7/49 (14.3)	24/56 (42.9)	18/53 (34.0)
>150 to ≤150 μg/g			
Number of patients with change in FCP levels from baseline, n/N (%)	9/47 (19.1)	24/52 (46.2)	20/52 (38.5)

FCP, fecal calprotectin.

Supplementary Table 5. Change From Baseline in Leukocyte and Neutrophil Counts at Weeks 12 and 52

	Induction (Week 12)			Maintenance (Week 52)		
	**Placebo**	**Ozanimod 0.46 mg**	**Ozanimod 0.92 mg**	**Placebo**	**Ozanimod 0.46 mg**	**Ozanimod 0.92 mg**
**Leukocytes, 10^9/L**						
n	65	68	65	65	68	65
Mean (SD) at baseline	6.5 (1.8)	6.7 (2.3)	6.7 (2.1)	6.5 (1.8)	6.7 (2.3)	6.7 (2.1)
n	58	59	59	12	34	35
Mean (SD) change from baseline	0.9 (2.4)	−1.8 (2.0)	−2.1 (1.7)	−0.2 (1.3)	−1.9 (2.1)	−2.0 (1.6)
**Neutrophils, 10^9/L**						
n	65	68	65	65	68	65
Mean (SD) at baseline	4.2 (1.5)	4.4 (2.0)	4.5 (2.0)	4.2 (1.5)	4.4 (2.0)	4.5 (2.0)
n	58	59	59	12	34	35
Mean (SD) change from baseline	1.1 (2.3)	−0.7 (1.8)	−0.9 (1.5)	−0.3 (1.3)	−0.6 (2.0)	−0.9 (1.5)

SD, standard deviation.

Supplementary Table 6. Patients With Abnormal Neutrophil Count or Leukocyte Count

	Induction (Week 12)			Maintenance (Week 52)		
	**Placebo**	**Ozanimod 0.46 mg**	**Ozanimod 0.92 mg**	**Placebo**	**Ozanimod 0.46 mg**	**Ozanimod 0.92 mg**
Baseline[a]						
n	65	68	65	65	68	65
ANC <1000 cells/mL	0	1 (1.5)	1 (1.5)	0	1 (1.5)	1 (1.5)
Total WBC >20,000 cells/μL	0	0	0	0	0	0
Overall[b]						
n	65	67	65	65	67	65
ANC <1000 cells/mL	0	1 (1.5)	0	0	1 (1.5)	0
Total WBC >20,000 cells/μL	1 (1.5)	0	0	1 (1.5)	0	0

[a]Baseline is defined as the last nonmissing record on or before the first dose of study drug.

[b]Patients' postbaseline assessments are used to derive abnormality.

ANC, absolute neutrophil count; WBC, white blood cell.

Supplementary Table 7. TEAEs With Incidence >2% (safety population)

	IP			IP and MP		
	**Placebo (n=65)**	**Ozanimod 0.46 mg (n=68)**	**Ozanimod 0.92 mg (n=65)**	**Placebo (n=65)**	**Ozanimod 0.46 mg (n=68)**	**Ozanimod 0.92 mg (n=65)**
TEAE with incidence ≥2%						
Nasopharyngitis	4 (6.2)	5 (7.4)	5 (7.7)	6 (9.2)	10 (14.7)	9 (13.8)
Pyrexia	2 (3.1)	6 (8.8)	2 (3.1)	3 (4.6)	11 (16.2)	7 (10.8)
Headache	4 (6.2)	4 (5.9)	4 (6.2)	4 (6.2)	8 (11.8)	6 (9.2)
Back pain	5 (7.7)	2 (2.9)	3 (4.6)	5 (7.7)	5 (7.4)	6 (9.2)
COVID-19	2 (3.1)	2 (2.9)	1 (1.5)	3 (4.6)	4 (5.9)	5 (7.7)
Colitis ulcerative	1 (1.5)	4 (5.9)	4 (6.2)	1 (1.5)	4 (5.9)	4 (6.2)
Arthralgia	1 (1.5)	2 (2.9)	3 (4.6)	2 (3.1)	4 (5.9)	4 (6.2)
Abdominal pain	1 (1.5)	2 (2.9)	0	1 (1.5)	4 (5.9)	2 (3.1)
GGT increased	0	1 (1.5)	1 (1.5)	0	5 (7.4)	2 (3.1)
Dental caries	1 (1.5)	3 (4.4)	0	2 (3.1)	5 (7.4)	0
Nausea	1 (1.5)	1 (1.5)	1 (1.5)	1 (1.5)	2 (2.9)	3 (4.6)
ALT increased	0	1 (1.5)	2 (3.1)	1 (1.5)	1 (1.5)	3 (4.6)
Constipation	0	1 (1.5)	1 (1.5)	0	1 (1.5)	3 (4.6)
Insomnia	1 (1.5)	1 (1.5)	1 (1.5)	1 (1.5)	1 (1.5)	3 (4.6)
Malaise	1 (1.5)	1 (1.5)	2 (3.1)	1 (1.5)	1 (1.5)	3 (4.6)
Hepatic function abnormal	1 (1.5)	0	2 (3.1)	1 (1.5)	0	3 (4.6)
Vertigo	0	0	1 (1.5)	0	0	3 (4.6)
Periodontal disease	0	0	1 (1.5)	0	2 (2.9)	2 (3.1)
Rash	1 (1.5)	2 (2.9)	1 (1.5)	1 (1.5)	2 (2.9)	2 (3.1)
Wound	0	0	2 (3.1)	0	2 (2.9)	2 (3.1)
AST increased	0	0	2 (3.1)	0	1 (1.5)	2 (3.1)
Contusion	0	0	1 (1.5)	0	1 (1.5)	2 (3.1)
Diarrhea	0	0	1 (1.5)	0	1 (1.5)	2 (3.1)
Herpes zoster	1 (1.5)	0	1 (1.5)	1 (1.5)	1 (1.5)	2 (3.1)
Liver function test increased	0	2 (2.9)	1 (1.5)	0	2 (2.9)	2 (3.1)
SARS-CoV-2 test positive	0	0	0	1 (1.5)	1 (1.5)	2 (3.1)
Cough	0	0	1 (1.5)	0	0	2 (3.1)
Dizziness	1 (1.5)	0	0	2 (3.1)	0	2 (3.1)
Ocular hypertension	0	0	1 (1.5)	0	0	2 (3.1)
Peripheral edema	2 (3.1)	0	1 (1.5)	3 (4.6)	0	2 (3.1)
Oropharyngeal discomfort	0	0	2 (3.1)	0	0	2 (3.1)
Orthostatic hypotension	0	0	0	0	0	2 (3.1)
Abdominal pain upper	1 (1.5)	2 (2.9)	1 (1.5)	1 (1.5)	2 (2.9)	1 (1.5)
Oropharyngeal pain	1 (1.5)	2 (2.9)	0	1 (1.5)	3 (4.4)	1 (1.5)
Oral herpes	0	0	0	0	2 (2.9)	1 (1.5)

Stomatitis	0	2 (2.9)	0	0	3 (4.4)	0
Animal bite	0	1 (1.5)	0	0	2 (2.9)	0
Cystitis	0	0	0	0	2 (2.9)	0
Dyspepsia	0	2 (2.9)	0	0	2 (2.9)	0
Epistaxis	0	2 (2.9)	0	0	2 (2.9)	0
Gastroenteritis	0	0	0	2 (3.1)	2 (2.9)	0
Hepatic enzyme increased	0	2 (2.9)	0	0	2 (2.9)	0
Hypertension	0	2 (2.9)	0	0	2 (2.9)	0
Large intestine polyp	0	1 (1.5)	0	0	2 (2.9)	0
Nasal vestibulitis	0	2 (2.9)	0	0	2 (2.9)	0
Noncardiac chest pain	0	1 (1.5)	0	0	2 (2.9)	0
Pain in extremity	1 (1.5)	1 (1.5)	0	1 (1.5)	2 (2.9)	0
Hordeolum	1 (1.5)	0	0	2 (3.1)	1 (1.5)	0
Immunization reaction	3 (4.6)	1 (1.5)	0	3 (4.6)	1 (1.5)	0

ALT, alanine aminotransferase; AST, aspartate aminotransferase; GGT, gamma-glutamyl transferase; IP, induction period; MP, maintenance period; TEAE, treatment-emergent adverse event.

Supplementary Table 8. Abnormal Electrocardiogram Values at Hour 6 in Patients With 6-Hour Cardiac Monitoring on Day 1 (induction period, safety population)

	**Placebo (N=65)**	**Ozanimod 0.46 mg (N=68)**	**Ozanimod 0.92 mg (N=65)**
n	24	21	22
QT >480 ms	0	0	0
QT >500 ms	0	0	0
QTcF >480 ms	0	1 (4.8)	0
QTcF >500 ms	0	1 (4.8)	0
Change from baseline in QT of >30 ms	1 (4.2)	4 (19.0)	7 (31.8)
Change from baseline in QT of >60 ms	0	1 (4.8)	0
Change from baseline in QTcF of >30 ms	0	1 (4.8)	1 (4.5)
Change from baseline in QTcF of >60 ms	0	1 (4.8)	0

Data are n (%).

QTcF, QT interval corrected for heart rate using Fridericia’s formula.

Supplementary Table 9. Electrocardiogram Findings in the Induction and Maintenance Periods (safety population)

	IP			IP and MP		
	**Placebo (N=65)**	**Ozanimod 0.46 mg (N=68)**	**Ozanimod 0.92 mg (N=65)**	**Placebo (N=65)**	**Ozanimod 0.46 mg (N=68)**	**Ozanimod 0.92 mg (N=645)**
n	65	67	64	65	67	64
QT >480 ms	0	0	0	0	1 (1.5)	0
QT >500 ms	0	0	0	0	1 (1.5)	0
QTcF >480 ms	0	1 (1.5)	0	0	2 (3.0)	0
QTcF >500 ms	0	1 (1.5)	0	0	2 (3.0)	0
Change from baseline in QT of >30 ms	6 (9.2)	15 (22.4)	14 (21.9)	6 (9.2)	18 (26.9)	17 (26.6)
Change from baseline in QT of >60 ms	0	2 (3.0)	0	0	4 (6.0)	0
Change from baseline in QTcF of >30 ms	0	3 (4.5)	3 (4.7)	0	5 (7.5)	4 (6.3)
Change from baseline in QTcF of >60 ms	0	1 (1.5)	0	0	2 (3.0)	0

Data are n (%).

IP, induction period; MP, maintenance period; QTcF, QT interval corrected for heart rate using Fridericia's formula.

Supplementary Table 10. Mean (standard deviation) Heart Rate and Change From Baseline in Heart Rate During Cardiac Monitoring on Study Day 1 Hours 1–6 (induction period, safety population)

	**Placebo (N=65)**	**Ozanimod 0.46 mg (N=68)**	**Ozanimod 0.92 mg (N=65)**
Pulse rate, supine (bpm), n	24	21	22
Mean (SD) HR			
Baseline	68.5 (10.0)	71.3 (10.3)	69.1 (8.8)
Hour 1	66.7 (9.1)	69.3 (9.9)	73.0 (9.2)
Hour 2	69.4 (9.5)	72.3 (10.4)	70.1 (8.9)
Hour 3	70.0 (9.5)	72.2 (9.4)	67.7 (10.3)
Hour 4	68.5 (7.6)	70.1 (9.0)	70.2 (8.0)
Hour 5	66.9 (7.1)	69.3 (9.6)	68.2 (9.5)
Hour 6	67.8 (9.9)	68.3 (8.6)	67.2 (8.2)
Mean (SD) change from baseline in HR			
Hour 1	−0.5 (5.6)	−2.2 (7.5)	0.4 (8.5)
Hour 2	2.2 (7.9)	0.7 (8.6)	−2.4 (7.3)
Hour 3	2.8 (8.5)	0.7 (8.4)	−4.8 (7.3)
Hour 4	1.3 (7.4)	−1.5 (9.7)	−2.4 (8.3)
Hour 5	−0.3 (6.2)	−2.3 (9.7)	−4.3 (7.7)
Hour 6	0.6 (8.0)	−3.3 (9.1)	−5.3 (7.8)
Pulse rate, standing (bpm), n	24	21	22
Mean (SD) HR			
Baseline	81.0 (14.0)	85.0 (12.2)	82.8 (11.3)
Hour 1	78.6 (11.6)	82.5 (12.4)	84.5 (9.5)
Hour 2	84.4 (12.5)	85.8 (14.7)	80.2 (9.7)
Hour 3	87.7 (13.1)	83.7 (13.1)	80.4 (8.7)
Hour 4	85.2 (9.4)	82.2 (10.1)	80.8 (9.6)
Hour 5	80.4 (9.6)	80.2 (9.7)	79.6 (11.3)
Hour 6	79.3 (9.7)	80.1 (9.3)	76.0 (9.4)
Mean (SD) change from baseline in HR			
Hour 1	−1.3 (7.8)	−1.4 (9.3)	1.9 (9.8)
Hour 2	4.5 (10.7)	1.9 (11.6)	−2.4 (8.7)
Hour 3	7.9 (11.8)	−0.2 (10.4)	−2.2 (8.5)
Hour 4	5.3 (10.2)	−1.7 (10.9)	−1.8 (9.6)
Hour 5	0.5 (10.3)	−3.7 (9.8)	−2.9 (12.0)
Hour 6	−0.6 (8.7)	−3.8 (8.6)	−6.5 (10.6)

bpm, beats per minute; HR, heart rate; SD, standard deviation.

Supplementary Figure 1. Study design of the phase 3 Japan-True North study.

[a]The maintenance period was initially planned as 52 weeks but was shortened to 40 weeks in an amendment to the protocol. This did not affect the timing of efficacy endpoints at week 52 for the maintenance period. [b]Patients were stratified by corticosteroid use at screening (yes or no) and prior biologic use (yes or no). [c]Ozanimod was initiated at a dose of 0.23 mg for 4 days, then ozanimod 0.46 mg for 3 days, followed thereafter by the assigned treatment level (ie, ozanimod 0.46 mg or ozanimod 0.92 mg). [d]Patients who did not achieve clinical response (defined as a reduction from baseline in the complete Mayo score ≥3 points and ≥30% and a reduction from baseline in the RBS of ≥1 point or an absolute RBS of ≤1 point) at week 12. [e]Disease relapse: increase in UC disease activity as defined by an increase in partial Mayo score ≥2 points compared with the week 12 partial Mayo score with an absolute partial Mayo score ≥4 points, an endoscopic subscore of ≥2 points, and exclusion of other causes of an increase in disease activity unrelated to underlying UC (eg, infections, change in medication). OLE, open-label extension; RBS, rectal bleeding subscore; UC, ulcerative colitis.

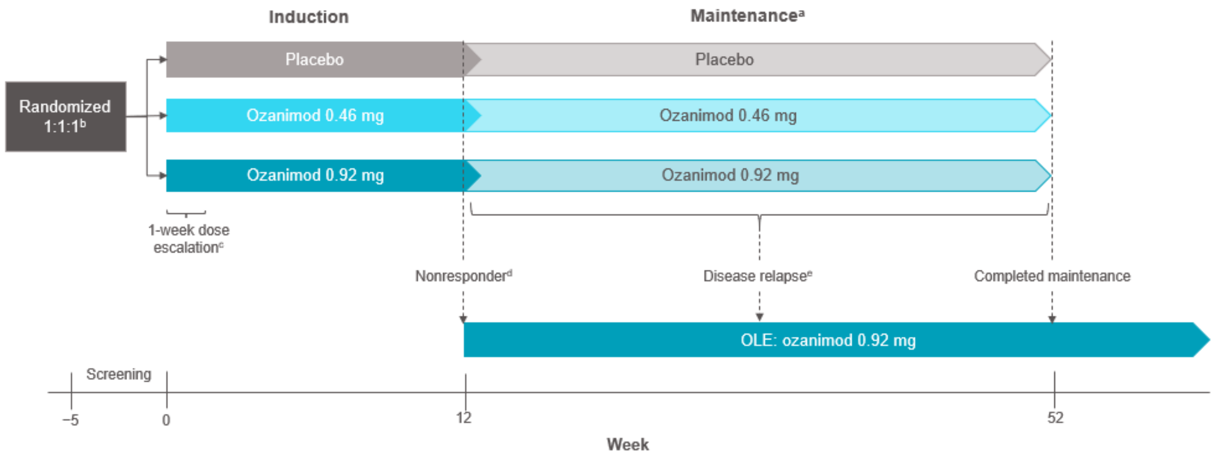

Supplementary Figure 2. Additional secondary and exploratory endpoints at weeks 12 and 52. Nonresponder imputation approach was used for handling of missing data. Weighted differences, 95% CIs, and *P* values for comparison between groups were based on the Cochran-Mantel-Haenszel test and were stratified by prior biologic agents and corticosteroid use (yes/no). [a]Clinical response: A reduction from baseline in the 9-point Mayo score of ≥2 points and ≥35%, and a reduction from baseline in the RBS of ≥1 point or an absolute RBS of ≤1 point. [b]Clinical remission: complete Mayo score of ≤2 points and with no individual subscore of >1 point. [c]Clinical remission: SFS = 0 or 1 (without a requirement of a decrease of ≥1 point from the baseline SFS), RBS = 0, and endoscopy subscore = 0 or 1. [d]Histologic remission: Geboes score <2.0. CI, confidence interval; RBS, rectal bleeding subscore; SFS, stool frequency subscore.

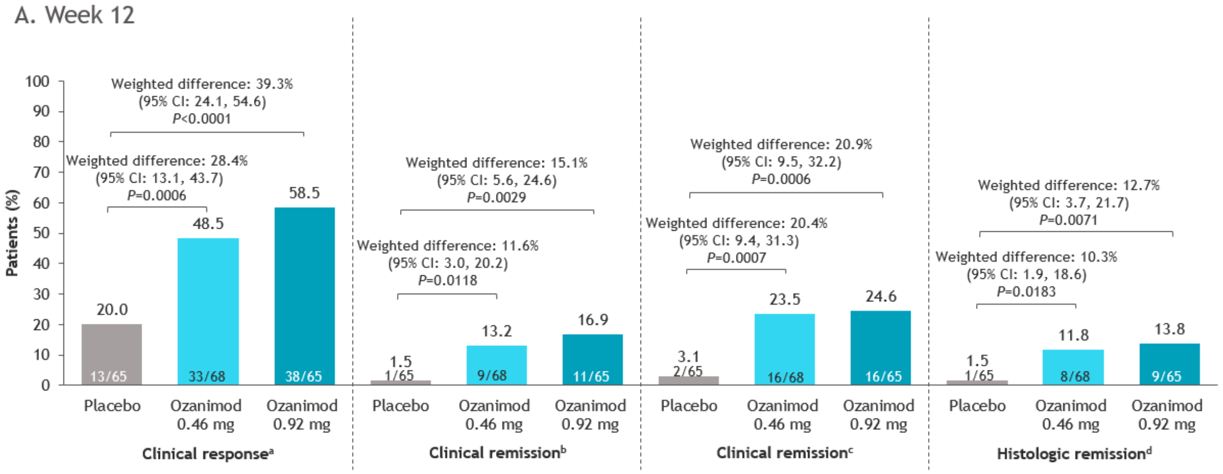

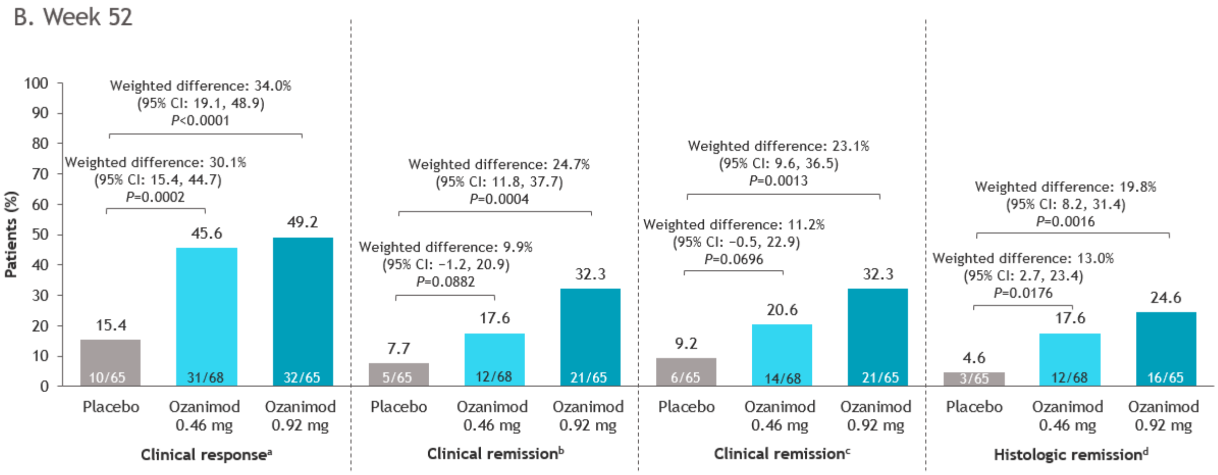
B. Week 52
Patients (%)
Weighted difference: 34.0%
(95% CI: 19.1, 48.9)
P<0.0001
Weighted difference: 30.1%
(95% CI: 15.4, 44.7)
P=0.0002
15.4
45.6
49.2
10/65
31/68
32/65
Placebo
Ozanimod 0.46 mg
Ozanimod 0.92 mg
Clinical response[a]
Weighted difference: 24.7%
(95% CI: 11.8, 37.7)
P=0.0004
Weighted difference: 9.9%
(95% CI: −1.2, 20.9)
P=0.0882
7.7
17.6
32.3
5/65
12/68
21/65
Clinical remission[b]
Weighted difference: 23.1%
(95% CI: 9.6, 36.5)
P=0.0013
Weighted difference: 11.2%
(95% CI: −0.5, 22.9)
P=0.0696
9.2
20.6
32.3
6/65
14/68
21/65
Clinical remission[c]
Weighted difference: 19.8%
(95% CI: 8.2, 31.4)
P=0.0016
Weighted difference: 13.0%
(95% CI: 2.7, 23.4)
P=0.0176
4.6
17.6
24.6
3/65
12/68
16/65
Histologic remission[d]

Supplementary Figure 3. Clinical response at week 12 by baseline characteristic subgroups. Nonresponder imputation approach is used for handling missing data. Treatment differences and *P* values for comparison between the active and placebo groups are based on the Cochran-Mantel-Haenszel test, stratified by prior biologic agents and CS use (yes or no). If the subgroup is the stratification factor, the Cochran-Mantel-Haenszel test is not stratified by this subgroup factor. 5-ASA, 5-aminosalicylic acid; ALC, absolute lymphocyte count; BL, baseline; CI, confidence interval; CS, corticosteroid; UC, ulcerative colitis.

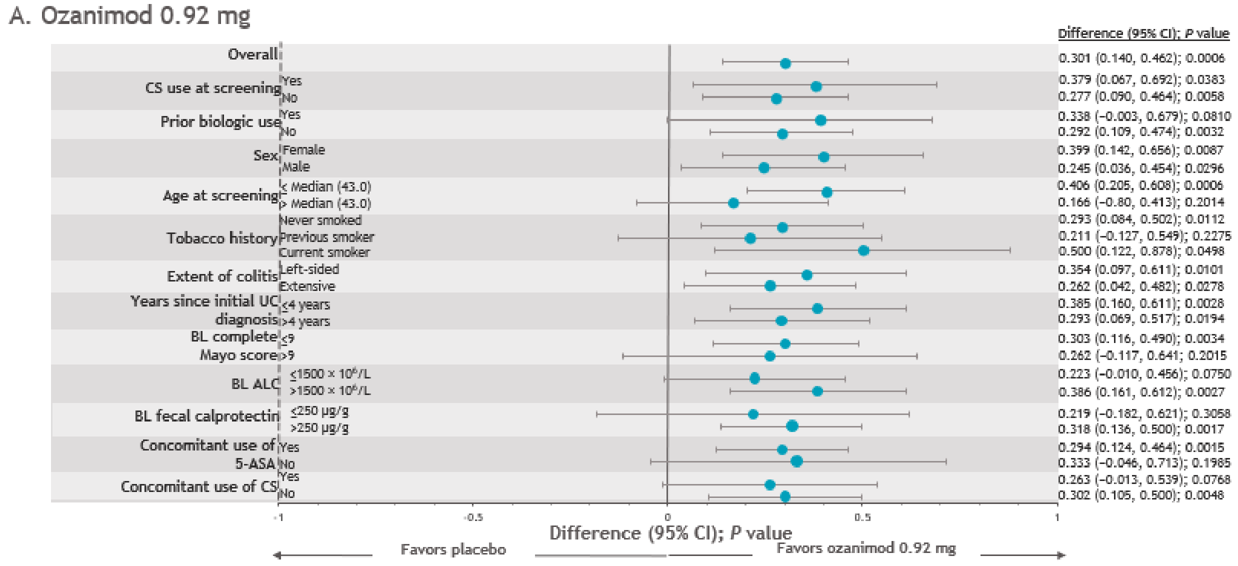

B. Ozanimod 0.46 mg

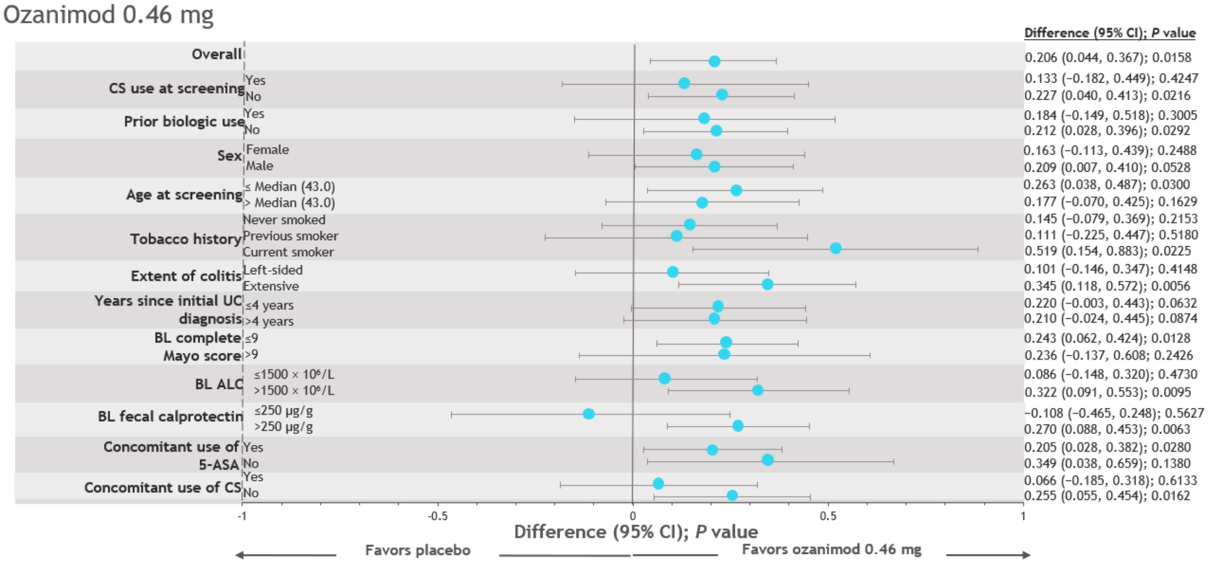

Supplementary Figure 4. Changes in absolute lymphocyte count over time. ALC, absolute lymphocyte count; SD, standard deviation; W, week.

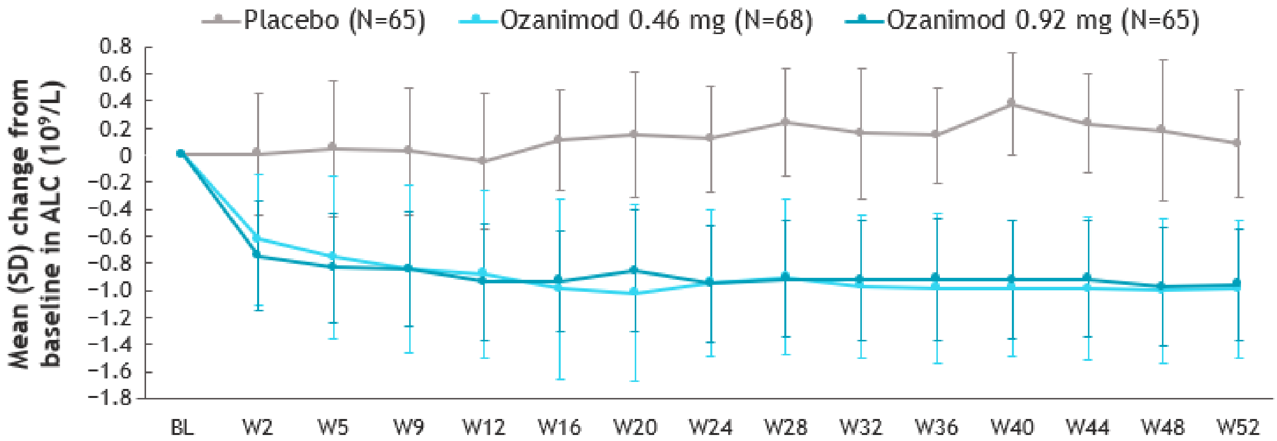

n	BL	W2	W5	W9	W12	W16	W20	W24	W28	W32	W36	W40	W44	W48	W52
Placebo	65	62	61	59	58	17	16	15	14	13	13	13	13	13	12
Ozanimod 0.46 mg	68	63	61	60	59	37	37	36	36	36	36	36	35	35	34
Ozanimod 0.92 mg	65	62	61	59	59	39	39	38	37	36	36	36	36	35	35

Plain Language Summary

Publication Plain Language Summary

Ozanimod Was Effective and Well Tolerated in Japanese Patients With Moderate to Severe Ulcerative Colitis

The full title of the article is "Once-Daily Oral Ozanimod for Japanese Patients With Ulcerative Colitis: Results From the Phase 2/3 J-True North Study"

You can find the full article here: [XXX]. You can access the full article for free

This publication plain language summary has been developed to accompany the article and is not intended for any other use.

What did this study look at?

Ulcerative colitis: What is it?

- Ulcerative colitis is a disease in which the lining of the colon and rectum becomes inflamed. Symptoms of ulcerative colitis include blood in the stool, diarrhea, and a feeling that you need to pass stools even though your bowels are already empty

People with untreated ulcerative colitis have a higher risk for

Anxiety and depression

Cancer of the colon and rectum

This disease can also negatively affect a person's job and social interactions

- To treat ulcerative colitis, doctors commonly prescribe anti-inflammatory medications, such as aminosalicylates and corticosteroids, as the first step. Medications known as immunomodulators, which reduce inflammation by decreasing the immune system response that starts the process of inflammation, may also be prescribed. If those medications do not work, medications known as biologics and Janus kinase inhibitors may be prescribed

The study: What is it about and why was it done?

- The efficacy and safety of ozanimod have not yet been evaluated in a large number of Japanese people
- Therefore, researchers conducted this J-True North study to see how once-daily ozanimod 0.46 mg or ozanimod 0.92 mg works in Japanese people (hereafter referred to as participants) with moderate to severe ulcerative colitis and to see what side effects it caused
- Based on findings from J-True North, once-daily ozanimod 0.92 mg was approved in Japan in December 2024 for the treatment of moderate to severe ulcerative colitis in participants who have had an inadequate response to standard treatments

Ozanimod: How does it work?

- Ozanimod is a sphingosine 1-phosphate (S1P) receptor 1 and 5 modulator that works to reduce inflammation by preventing lymphocytes, a kind of white blood cell, from moving into inflamed tissues

Ozanimod is a capsule taken by mouth

Participants who start ozanimod need to slowly increase the dose of ozanimod over a week to reach the once-daily dose of 0.92 mg

Ozanimod 0.23 mg once daily	Ozanimod 0.46 mg once daily	Ozanimod 0.92 mg once daily
Days 1-4	Days 5-7	Day 8 and beyond

The dose of ozanimod is increased slowly to reduce the chance of side effects on the heart.

- Ozanimod is approved to treat moderate to severe ulcerative colitis and multiple sclerosis in the United States and several other countries

The summary: What will this cover?

- This plain language summary describes a clinical study called J-True North, which looked at the effects of ozanimod in Japanese people with moderate to severe ulcerative colitis. This study compared changes in symptoms between people who took placebo and ozanimod to see if ozanimod worked. The placebo looked like ozanimod but did not have the active medication in it. Researchers also studied the side effects of ozanimod. This allowed them to determine whether ozanimod is safe and if it improved symptoms, such as blood in the stool and diarrhea

How was the study done?

Study start and end date
June 3, 2019, to August 28, 2023

Phase of study
Phase 2/3

Study status
Results reported within the study are final

Key inclusion criteria

- Japanese adults (18-75 years old) with ulcerative colitis for at least the past 3 months
- These adults must have previously taken aminosalicylates or corticosteroids to treat their ulcerative colitis

Key exclusion criteria

- People were excluded if they had serious inflammation of a large portion of their colon, known as severe extensive colitis, or if they had Crohn's disease, certain heart conditions, or a history of type 1 diabetes or uncontrolled type 2 diabetes

Randomization: Participants with ulcerative colitis were randomly assigned (or "randomized") to take once-daily ozanimod 0.46 mg, ozanimod 0.92 mg, or placebo

The placebo looks like ozanimod, but it does not have any medicine in it

This study was "blinded," meaning that participants in the study and the researchers did not know who took ozanimod and who took placebo

Screening (5 weeks)

Researchers checked to see whether people with ulcerative colitis could take part in this study based on the study's inclusion and exclusion criteria

Induction period (12 weeks)

Participants with ulcerative colitis took ozanimod or placebo for 12 weeks

After the 12 weeks, participants with ulcerative colitis who had clinical response, meaning whose symptoms were better, could enter the maintenance period of the study

Maintenance period (40 weeks)

Participants with ulcerative colitis continued to take the same medicine (ozanimod or placebo) as they did during the induction period for an additional 40 weeks

Week −5 | 0 | 12 | 52

In this study, researchers determined whether people with ulcerative colitis had clinical response by seeing whether their symptoms improved (for example, by having less blood in the stool, less diarrhea, or less abdominal pain) and by using a small camera to see whether the colon looked less swollen

- The study looked at the percentages of participants with clinical response after 12 weeks of treatment and after 52 weeks of taking ozanimod
- These percentages of participants with clinical response after taking ozanimod were compared with the percentages of participants with clinical response after taking placebo
- Researchers also looked at the side effects that participants had during the study

Who took part in this study?

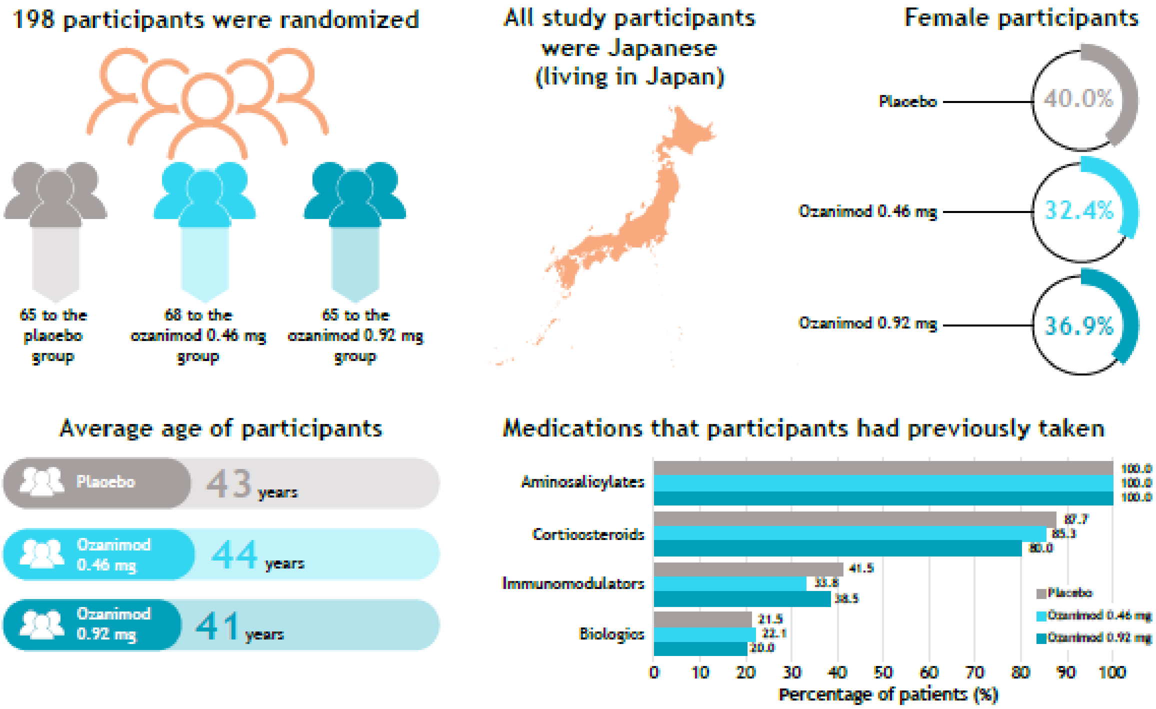

What were the results of the study?

- After taking ozanimod for 12 weeks (either dose), more than half of the participants had clinical response compared with less than one-third of those in the placebo group

32.3%
52.9%
61.5%

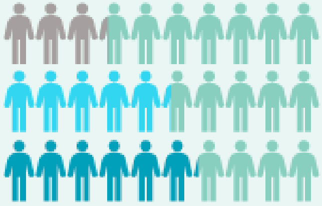

- After taking ozanimod for 52 weeks (either dose), almost half of the people had clinical response compared with only 16.9% in the placebo group

16.9%
47.1%
49.2%

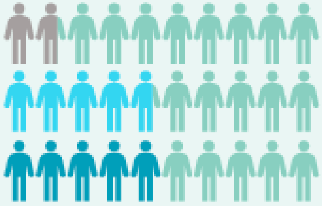

- ≥80% of participants who achieved clinical response receiving ozanimod 0.46 mg or ozanimod 0.92 mg at the end of the induction period showed clinical response at the end of the maintenance period

≥80%

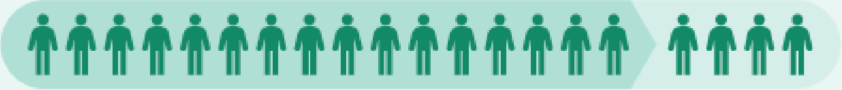

- In the induction and maintenance periods of the study, the following were the most common side effects in participants taking ozanimod:
- Researchers were interested in knowing whether participants taking ozanimod had side effects seen in patients who took S1P modulators, the same class of medications as ozanimod

	Cold symptoms	Fever	Headache	Low heart rate	Cancer	Shingles	Swelling in part of the retina
Placebo	9.2%	4.6%	6.2%	None	None	1.5%	None
Ozanimod 0.46 mg	14.7%	16.2%	11.8%	None	None	1.5%	None
Ozanimod 0.92 mg	13.8%	10.8%	9.2%	None	None	3.1%	1.5%

What were the study's main conclusions?

- Ozanimod was effective and well tolerated as a once-daily oral medication in Japanese participants with moderate to severe ulcerative colitis
- This Japanese clinical study was the first large-scale study examining ozanimod in an Asian population. The results suggest that ozanimod is effective and safe for Asians
- The efficacy and safety results of ozanimod in the J-True North study were similar to the findings from a global study of ozanimod called True North

Who sponsored this study?

This clinical study was sponsored by Bristol Myers Squibb, which thanks everybody who participated in the study

Where can I find additional information?

The original article was published in the medical journal *Gastro Hep Advances*. Please refer to the original article for complete author-disclosure information

You can find more information about this study here:
Study Details | To Evaluate Efficacy and Long-term Safety of Ozanimod in Japanese Subjects With Moderately to Severely Active Ulcerative Colitis | https://clinicaltrials.gov/study/NCT03915769

SUPPLEMENTARY MATERIAL

Supplementary Methods

Inclusion Criteria

1. Japanese adults aged 18 to 75 years at the time of signing the informed consent form at screening.
2. Patient has had ulcerative colitis (UC) diagnosed ≥3 months prior to first investigational product administration. The diagnosis should be confirmed by clinical and endoscopic evidence and corroborated by a histopathology report (endoscopy and histopathology may be performed at screening if no prior report is readily available).
3. Patient has evidence of UC extending ≥15 cm from the anal verge as determined by baseline endoscopy (flexible sigmoidoscopy or colonoscopy).
4. Patient has active UC defined as Mayo score of 6–12 inclusive, with Mayo endoscopy subscore of ≥2, a rectal bleeding subscore of ≥1, and a stool frequency subscore ≥1.
5. Patient must have been treated with aminosalicylates or corticosteroids. If patients are currently receiving treatment with ≥1 of the following therapies, the patient must continue on these therapies during Induction:
    a. Oral aminosalicylates at a therapeutic dose for their disease (eg, mesalazine, sulfasalazine), with the dose stable for ≥2 weeks prior to screening endoscopy.
    b. Prednisolone (doses ≤10 mg/day) or equivalent receiving a stable dose for ≥2 weeks prior to screening endoscopy.
6. Patient has undergone colonoscopy (or is willing to undergo colonoscopy during screening):
    a. Within the past 2 years, to screen for dysplasia (unless otherwise recommended by local and national guidelines) if the patient has had left-sided colitis for >12 years or total/extensive colitis for >8 years.
    b. Within the past 5 years, to screen for polyps if the patient age is >45 years.

7. If oral aminosalicylates or corticosteroids have been recently discontinued, patient must have been stopped for ≥2 weeks prior to the endoscopy used for baseline Mayo score.
8. Females of childbearing potential:
    a. Patient must agree to practice a highly effective method of contraception throughout the study until completion of the 90-day safety follow-up visit. Highly effective methods of contraception are those that alone or in combination result in a failure rate of a Pearl Index of <1% per year when used consistently and correctly. The following are acceptable methods of birth control in the study:
        i. Combined hormonal (containing estrogen and progestogen) contraception, which may be oral, intravaginal, or transdermal
        ii. Progestogen-only hormonal contraception associated with inhibition of ovulation, which may be oral, injectable, or implantable
        iii. Placement of an intrauterine device
        iv. Placement of a hormone-releasing intrauterine system
        v. Bilateral tubal occlusion
        vi. Vasectomized partner
        vii. Complete sexual abstinence
    b. A female of childbearing potential is a sexually mature female who (1) has not undergone a hysterectomy (the surgical removal of the uterus) or bilateral oophorectomy (the surgical removal of both ovaries) or (2) has not been postmenopausal for ≥24 consecutive months (ie, has had menses at any time during the preceding 24 consecutive months). Periodic abstinence (calendar, symptothermal, postovulation methods), withdrawal (coitus interruptus), spermicides only, and the lactational amenorrhea method are not acceptable methods of contraception.
9. Patient provides written informed consent and confirms compliance with the schedule of protocol assessments.

10. Patients must have documentation of positive varicella zoster virus immunoglobulin G antibody status or complete varicella zoster virus vaccination ≥30 days prior to randomization.

Exclusion Criteria

Exclusions Related to General Health

1. Patient has severe extensive colitis as evidenced by:
   a. Physician judgment that the patient is likely to require colectomy or ileostomy within 12 weeks of baseline.
   b. Current or recent (within 3 months of screening) evidence of fulminant colitis, toxic megacolon, or bowel perforation.
2. Patient has a diagnosis of Crohn’s disease, indeterminate colitis, the presence or history of a fistula consistent with Crohn’s disease, microscopic colitis, radiation colitis, or ischemic colitis.
3. Patient has a positive stool examination for pathogens (ova and parasites, bacteria) or a positive test for toxin producing Clostridioides difficile (C. difficile) at screening. Polymerase chain reaction examination of the stool for C. difficile may be used to exclude false positives. If positive, patients may be treated and retested. Documentation of a negative test result for pathogens (ova and parasites, bacteria) is required within 60 days of day 1.
4. Patient is pregnant or breastfeeding, or has a positive serum beta-human chorionic gonadotropin measured during screening.
5. Patient has clinically relevant hepatic, neurological, pulmonary (severe respiratory disease, pulmonary fibrosis and chronic obstructive pulmonary disease), ophthalmological, endocrine, psychiatric, or other major systemic disease making implementation of the protocol or interpretation of the study difficult or that would put the patient at risk by participating in the study.
6. Patient has clinically relevant cardiovascular conditions, including history or presence of:
   a. Recent (within the last 6 months of screening) occurrence of myocardial infarction, unstable angina, stroke, transient ischemic attack, symptomatic

bradycardia, decompensated heart failure requiring hospitalization, class III/IV heart failure, or severe untreated sleep apnea.

b. Second degree (Mobitz type II) atrioventricular block, third-degree atrioventricular block, sick sinus syndrome, or sinoatrial block in patients without a pacemaker in place; if second-degree Type II or third-degree atrioventricular block is due to concomitant medication, consult the medical monitor prior to screening.

c. Prolonged QT interval corrected for heart rate using Fridericia's formula (QTcF; QTcF >450 msec males, >470 msec females) at either screening or day 1 predose assessment. One recheck is allowed for patients per visit (ie, during the screening and/or day 1 predose assessment visit).

d. Resting heart rate <55 beats per minute when taking vital signs as part of the physical examination at either screening or day 1 predose assessment. One recheck is allowed for patients with heart rate <55 beats per minute per visit (ie, during the screening and/or day 1 predose assessment visit).

e. Patients with the preexisting cardiac conditions listed below must be seen by a consulting cardiologist and cleared to participate in the study without more intensive monitoring. These patients will also follow first-dose monitoring procedures.

    i. History (>6 months prior to screening) of ischemic heart disease, cardiac arrest, cerebrovascular disease, uncontrolled hypertension, history of recurrent syncope, or symptomatic bradycardia

    ii. Second-degree (Mobitz type II) atrioventricular block, third-degree atrioventricular block, sick sinus syndrome, or sinoatrial block with functional pacemaker

    iii. Patients on medicinal products that may potentiate bradycardia (other than the combination of beta-blockers and calcium channel blockers)

7. Patient has a history of diabetes mellitus type 1 or uncontrolled diabetes mellitus type 2 with glycosylated hemoglobin >9%; or diabetic patients with significant

comorbid conditions (eg, retinopathy, nephropathy).

8. Patient has a history of uveitis (within the last year) or a history of macular edema.
9. Patient has a known active bacterial, viral, or fungal infection (excluding fungal infection of nail beds, minor upper respiratory tract infections, and minor skin infections), a mycobacterial infection (including tuberculosis or atypical mycobacterial disease), or any major episode of infection that either required hospitalization or treatment with intravenous antibiotics within 30 days of screening, or treatment with oral antibiotics within 14 days of screening.
    a. In the case of a known SARS-CoV-2 infection, symptoms must have completely resolved; based on investigator assessment in consultation with the clinical trial physician/medical monitor, there are no sequelae that would place the patient at a higher risk of receiving investigational treatment.
10. Patient has a history or known presence of recurrent or chronic infection (eg, hepatitis A, B, or C; HIV); recurrent urinary tract infections are allowed.
11. Patient has a history of cancer, including solid tumors and hematological malignancies (except basal cell and in situ squamous cell carcinomas of the skin or uterine cervix that have been excised and resolved), or colonic mucosal dysplasia.
12. Patient has a history of alcohol or drug abuse within 1 year prior to randomization.
13. Patient has a history of or currently active primary or secondary immunodeficiency.

Exclusions Related to Medications

1. Patient has a history of treatment with a biologic agent within 8 weeks or 5 elimination half-lives (whichever is less) of that agent prior to randomization.
2. Patient has a history of treatment with tofacitinib within 5 elimination half-lives of that agent prior to randomization.
3. Patient has a history of treatment with an investigational agent within 5 elimination half-lives of that agent prior to randomization.

4. Patient has a history of treatment with topical rectal 5-aminosalicylic acid or topical rectal steroids within 2 weeks of screening endoscopy or antimotility medications (such as diphenoxylate/atropine) during screening.
5. Patient has received a live vaccine or live attenuated vaccine within 4 weeks prior to randomization.
6. Patient has been treated previously with lymphocyte-depleting therapies (eg, alemtuzumab, anti-CD4, cladribine, rituximab, cyclophosphamide, mitoxantrone, total body irradiation, bone marrow transplantation).
7. Patient has been treated with cyclosporine, tacrolimus, sirolimus, or mycophenolate mofetil within 16 weeks of screening. If no renal disorder or cutaneous malignancy is confirmed, the washout period of tacrolimus can be reduced to 8 weeks. To check renal disorder, serum creatinine/glomerular filtration rate, albumin-to-creatinine ratio, and serum potassium should be measured.
8. Patient has been treated previously with D-penicillamine, leflunomide, or thalidomide.
9. Patient has been treated previously with natalizumab, fingolimod, or other sphingosine 1-phosphate receptor modulators.
10. Patient has a history of treatment with intravenous immunoglobulin or plasmapheresis within 3 months prior to randomization.
11. Patient has planned concurrent treatment with antineoplastic immunosuppressive agents (ie, azathioprine or methotrexate) after randomization. Patients receiving azathioprine or methotrexate at screening must discontinue treatment with these agents 4 weeks prior to randomization.
12. Patient is treated with chronic nonsteroidal antiinflammatory drugs (occasional use of nonsteroidal antiinflammatory drugs and acetaminophen [for headache, arthritis, myalgias, or menstrual cramps] and aspirin up to 325 mg/day is permitted).
13. Patient is treated with class Ia or class III antiarrhythmic drugs or with ≥2 agents in a combination known to prolong PR interval, or treatment with additional prohibited systemic cardiac medications.

14. Patient has been treated with apheresis within 2 weeks of randomization.
15. Patients who were primary nonresponders to ≥2 biologic agents approved for the treatment of UC (ie, anti–tumor necrosis factor agents or vedolizumab).
16. Patient is receiving treatment with breast cancer resistance protein inhibitors (eg, cyclosporine, eltrombopag).
17. Patient is receiving treatment with any of the following drugs or interventions within the corresponding timeframe:
    a. At randomization
        i. CYP2C8 inhibitors (eg, clopidogrel) or inducers (eg, rifampicin)
    b. 2 weeks prior to randomization
        i. Monoamine oxidase inhibitors (eg, selegiline).

Exclusions Related to Laboratory Results and Other Assessments

18. Patient had the following laboratory results:
    a. Serum creatinine >1.4 mg/dL for females or >1.6 mg/dL for males
    b. Liver function impairment or persisting elevations of aspartate aminotransferase or alanine aminotransferase >2 × the upper limit of normal (ULN) or direct bilirubin >1.5 × ULN
    c. Platelet count <100,000/μL
    d. Hemoglobin <8.0 g/dL
    e. Neutrophils <1500 /μL
    f. Absolute white blood cell count <3500/μL
    g. Absolute lymphocyte count <800/μL
    h. ECG showing any clinically significant abnormality

Study Design

Open-label extension (OLE)

Patients who experienced disease relapse during the maintenance period were eligible to enter the OLE if they met all of the following criteria: increase in UC disease activity as defined by an increase in partial Mayo score of ≥2 points compared with the week 12 partial Mayo score with an absolute partial Mayo score ≥4 points, Mayo endoscopy subscore ≥2 points, and exclusion of other causes of an increase in disease activity

unrelated to underlying UC (eg, infections, change in medication). Patients withdrew from the OLE if they did not achieve clinical improvement after 12 weeks of ozanimod treatment in the OLE. The OLE will continue until marketing launch or until the sponsor discontinues the development program.

Supplementary Table 1. List of Japan-True North Study Principal Investigators, Sites, and Institutional or Ethical Review Boards

Site No.	Principal investigator name	Name/address of EIC/IRB
101	Kawaratani, Hideto	Nara Medical University Hospital
102	Matsuoka, Katsuyoshi	Toho University Medical Center Sakura Hospital
103	Motoya, Satoshi	Sapporo-Kosei General Hospital
104	Ishigami, Keisuke	Sapporo Medical University Hospital
105	Watanabe, Kenji	Hyogo College of Medicine Hospital
106	Oka, Shiro	Hiroshima University Hospital
107	Nakano, Masaru	Kitasato University Kitasato Institute Hospital
108	Saruta, Masayuki	Jikei University Hospital
109	Hisamatsu, Tadakazu	Kyorin University Hospital
110	Matsumoto, Takayuki	Iwate Medical University Uchimaru Medical Center
111	Takedatsu, Hidetoshi	Kurume University Hospital
112	Inaba, Tomoki	Kagawa Prefectural Central Hospital
113	Bamba, Shigeki	Shiga University of Medical Science Hospital
114	Hasatani, Kenkei	Fukui Prefectural Hospital
115	Kimura, Tsuguhiro	Medical Corporation Shoyu-Kai Fujita Gastroenterology Hospital
116	Tokito, Satoki	Tokitokai Tokito Clinic
117	Fukata, Masayuki	Tokyo Yamate Medical Center
118	Ninomiya, Tomoyuki	Ehime Prefectural Central Hospital
119	Hisanaga, Yasuhiro	Ogaki Municipal Hospital
120	Ito, Toru	Kanazawa Medical University Hospital
121	Matano, Yutaka	Komatsu Municipal Hospital
122	Hisabe, Takashi	Fukuoka University Chikushi Hospital
123	Horiki, Noriyuki	Mie University hospital
124	Munemoto, Yoshinori	Fukui-ken Saiseikai Hospital
125	Takamura, Masaaki	Nagaoka Chuo General Hospital
126	Kamoshida, Toshiro	Hitachi General Hospital
127	Sato, Yuichiro	Osaki Citizen Hospital
128	Minato, Yohei	NTT Medical Center Tokyo
129	Kamiyamamoto, Shinji	Toyama City Hospital
130	Kodama, Yuzo	Kobe University Hospital
131	Imaeda, Hiroyuki	Saitama Medical University Hospital
132	Hosomi, Shuhei	Osaka Metropolitan University
133	Sakuraba, Hirotake	Hirosaki University Hospital
134	Matsushima, Masashi	Tokai University Hospital
135	Nakatsu, Morihito	Mitoyo General Hospital
136	Yoshioka, Masao	Okayama Saiseikai Outpatient Center Hospital
137	Tanaka, Toshio	Shizuoka City Shizuoka Hospital
138	Fujii, Toshimitsu	Institute of Science Tokyo
139	Arai, Takehiro	Tokatsu Tsujinaka Hospital
140	Yamazaki, Kenji	Gifu Prefectural General Medical Center
141	Naito, Yuji	University Hospital Kyoto Prefectural University of Medicine
142	Kawano, Hiroshi	Our Lady of the Snow Social Medical Corporation St. Mary's Hospital
143	Kanda, Naoki	Takatsuki Red Cross Hospital
144	Ishino, Atsushi	Hoshi General Hospital
145	Hiramatsu, Naoki	Osaka Rosai Hospital

146	Kumagai, Shinji	IMS Meirikai Sendai General Hospital
147	Furuya, Ken	Japan Community Health Care Organization Hokkaido Hospital
148	Kanke, Kazunari	Kanke Gastrointestinal Clinic
149	Ohnishi, Yoshifumi	National Hospital Organization Shizuoka Medical Center
150	Sai, Souken	Sai Gastroenterology and Proctology Clinic
151	Kobayashi, Toshihisa	Hakodate Goryoukaku Hospital
152	Kuroda, Tsuyoshi	Mazda Hospital of Mazda Motor Corporation
153	Fukuchi, Takumi	Iseikai Hospital
154	Sakata, Yasuhisa	Saga University Hospital
155	Aoyagi, Kunihiko	Japanese Red Cross Fukuoka Hospital
156	Hiraoka, Sakiko	Okayama University Hospital
157	Kato, Shingo	Saitama Medical Center, Saitama Medical University
158	Takeuchi, Ken	Tsujinaka Hospital Kashiwanoha
159	Ishida, Tetsuya	Ishida Clinic of IBD and Gastroenterology
160	Watanabe, Chiyuki	Hiroshima Prefectural Hospital
161	Ochiai, Toshiaki	Saiseikai Fukuoka General Hospital
162	Kubokawa, Masaru	Aso Iizuka Hospital
163	Aoyama, Nobuo	Aoyama Clinic GI Endoscopy and IBD Center
164	Haraguchi, Kazuhiro	Hara Sanshin Hospital
165	Hidaka, Hisamitsu	Hidaka Coloproctology Clinic
166	Tobita, Kouji	Hiratsuka Gastroenterological Hospital
167	Yamamura, Takeshi	Nagoya University Hospital

EIC, ethical review board; IRB, institutional review board.

Supplementary Table 2. Definitions of Efficacy Endpoints

**Efficacy endpoints**	**Definition**
Clinical response	• Complete Mayo score definition: a reduction from baseline in the complete Mayo score of ≥3 points and ≥30% and a reduction from baseline in the RBS of ≥1 point or an absolute RBS of ≤1 point<br>− Complete Mayo score: sum of SFS, RBS, Mayo endoscopy subscore, Physician Global Assessment (each assessment rated from 0–3)<br>• 9-point Mayo score definition: reduction from baseline in the 9-point Mayo score of ≥2 points and ≥35%, and a reduction from baseline in the RBS of ≥1 point or an absolute RBS of ≤1 point<br>− 9-point Mayo score: sum of RBS, SFS, and Mayo endoscopy subscore (each assessment rated from 0–3)
Clinical remission	• Definition 1: complete Mayo score of ≤2 points with no individual subscore ≥1 point<br>• Definition 2: RBS = 0 and SFS ≤1 (and a decrease of ≥1 point from baseline SFS) and Mayo endoscopy subscore ≤1<br>• Definition 3: SFS = 0 or 1 (without a requirement of a decrease of ≥1 from baseline SFS), RBS = 0, endoscopy subscore = 0 or 1

Endoscopic improvement	• Endoscopy subscore ≤1
Mucosal healing	• Endoscopy subscore ≤1 with a Geboes score <2.0
Histologic remission	• Geboes score <2.0

RBS, rectal bleeding subscore; SFS, stool frequency subscore.

Supplementary Table 3. Change From Baseline in FCP and CRP Levels at Weeks 12 and 52

	Induction (Week 12)			Maintenance (Week 52)		
	**Placebo**	**Ozanimod 0.46 mg**	**Ozanimod 0.92 mg**	**Placebo**	**Ozanimod 0.46 mg**	**Ozanimod 0.92 mg**
**FCP, µg/g**						
n	65	68	65	65	68	65
Mean (SD) at baseline	2168.6 (3173.2)	2649.0 (3532.7)	2784.4 (4418.4)	2168.6 (3173.2)	2649.0 (3532.7)	2784.4 (4418.4)
Median (range) at baseline	1060.0 (12.7, 16,800.0)	1500.0 (26.6, 15,200.0)	885.0 (19.6, 22,200.0)	1060.0 (12.7, 16,800.0)	1500.0 (26.6, 15,200.0)	885.0 (19.6, 22,200.0)
n	59	59	59	12	34	35
Mean (SD) change from baseline	−230.1 (4248.0)	−1693.2 (3674.3)	−1581.0 (5379.3)	−1445.4 (1504.7)	−2015.9 (3426.2)	−2449.1 (6344.0)
Median (range) change from baseline	−28.4 (−16,781.7, 10,340.0)	−567.0 (−15,190.0, 7671.5)	−494.2 (−21,340.0, 17,100.0)	−986.0 (−3961.0, 495.3)	−1168.5 (−15,190.0, 6963.0)	−638.3 (−22,185.2, 17,608.0)
**CRP, mg/L**						
n	65	68	65	65	68	65
Mean (SD) at baseline	3.7 (7.6)	4.5 (8.4)	3.5 (6.4)	3.7 (7.6)	4.5 (8.4)	3.5 (6.4)
Median (range) at baseline	1.4 (0.1, 49.7)	1.7 (0.1, 54.2)	1.6 (0.1, 42.7)	1.4 (0.1, 49.7)	1.7 (0.1, 54.2)	1.6 (0.1, 42.7)
n	59	59	59	12	34	35
Mean (SD) change from baseline	−0.5 (5.5)	−2.3 (7.9)	−1.2 (7.2)	−0.4 (6.1)	−1.4 (5.8)	−1.5 (3.6)
Median (range) change from baseline	−0.0 (−25.5, 15.6)	−0.7 (−52.3, 2.3)	−0.2 (−30.8, 35.1)	−0.6 (−8.7, 17.0)	−0.6 (−28.4, 9.5)	−0.5 (−14.7, 4.4)

CRP, C-reactive protein; FCP, fecal calprotectin; SD, standard deviation.

Supplementary Table 4. FCP Response at Week 12

**FCP, µg/g**	**Placebo (N=65)**	**Ozanimod 0.46 mg (N=68)**	**Ozanimod 0.92 mg (N=65)**
>50 to ≤50 µg/g			
Number of patients with change in FCP levels from baseline, n/N (%)	3/55 (5.5)	19/57 (33.3)	13/57 (22.8)
>100 to ≤100 µg/g			
Number of patients with change in FCP levels from baseline, n/N (%)	7/49 (14.3)	24/56 (42.9)	18/53 (34.0)
>150 to ≤150 µg/g			
Number of patients with change in FCP levels from baseline, n/N (%)	9/47 (19.1)	24/52 (46.2)	20/52 (38.5)

FCP, fecal calprotectin.

Supplementary Table 5. Change From Baseline in Leukocyte and Neutrophil Counts at Weeks 12 and 52

	Induction (Week 12)			Maintenance (Week 52)		
	**Placebo**	**Ozanimod 0.46 mg**	**Ozanimod 0.92 mg**	**Placebo**	**Ozanimod 0.46 mg**	**Ozanimod 0.92 mg**
**Leukocytes, 10^9/L**						
n	65	68	65	65	68	65
Mean (SD) at baseline	6.5 (1.8)	6.7 (2.3)	6.7 (2.1)	6.5 (1.8)	6.7 (2.3)	6.7 (2.1)
n	58	59	59	12	34	35
Mean (SD) change from baseline	0.9 (2.4)	−1.8 (2.0)	−2.1 (1.7)	−0.2 (1.3)	−1.9 (2.1)	−2.0 (1.6)
**Neutrophils, 10^9/L**						
n	65	68	65	65	68	65
Mean (SD) at baseline	4.2 (1.5)	4.4 (2.0)	4.5 (2.0)	4.2 (1.5)	4.4 (2.0)	4.5 (2.0)
n	58	59	59	12	34	35
Mean (SD) change from baseline	1.1 (2.3)	−0.7 (1.8)	−0.9 (1.5)	−0.3 (1.3)	−0.6 (2.0)	−0.9 (1.5)

SD, standard deviation.

Supplementary Table 6. Patients With Abnormal Neutrophil Count or Leukocyte Count

	Induction (Week 12)			Maintenance (Week 52)		
	**Placebo**	**Ozanimod 0.46 mg**	**Ozanimod 0.92 mg**	**Placebo**	**Ozanimod 0.46 mg**	**Ozanimod 0.92 mg**
Baseline[a]						
n	65	68	65	65	68	65
ANC <1000 cells/mL	0	1 (1.5)	1 (1.5)	0	1 (1.5)	1 (1.5)
Total WBC >20,000 cells/μL	0	0	0	0	0	0
Overall[b]						
n	65	67	65	65	67	65
ANC <1000 cells/mL	0	1 (1.5)	0	0	1 (1.5)	0
Total WBC >20,000 cells/μL	1 (1.5)	0	0	1 (1.5)	0	0

[a]Baseline is defined as the last nonmissing record on or before the first dose of study drug.

[b]Patients' postbaseline assessments are used to derive abnormality.

ANC, absolute neutrophil count; WBC, white blood cell.

Supplementary Table 7. TEAEs With Incidence >2% (safety population)

	IP			IP and MP		
	**Placebo (n=65)**	**Ozanimod 0.46 mg (n=68)**	**Ozanimod 0.92 mg (n=65)**	**Placebo (n=65)**	**Ozanimod 0.46 mg (n=68)**	**Ozanimod 0.92 mg (n=65)**
TEAE with incidence ≥2%						
Nasopharyngitis	4 (6.2)	5 (7.4)	5 (7.7)	6 (9.2)	10 (14.7)	9 (13.8)
Pyrexia	2 (3.1)	6 (8.8)	2 (3.1)	3 (4.6)	11 (16.2)	7 (10.8)
Headache	4 (6.2)	4 (5.9)	4 (6.2)	4 (6.2)	8 (11.8)	6 (9.2)
Back pain	5 (7.7)	2 (2.9)	3 (4.6)	5 (7.7)	5 (7.4)	6 (9.2)
COVID-19	2 (3.1)	2 (2.9)	1 (1.5)	3 (4.6)	4 (5.9)	5 (7.7)
Colitis ulcerative	1 (1.5)	4 (5.9)	4 (6.2)	1 (1.5)	4 (5.9)	4 (6.2)
Arthralgia	1 (1.5)	2 (2.9)	3 (4.6)	2 (3.1)	4 (5.9)	4 (6.2)
Abdominal pain	1 (1.5)	2 (2.9)	0	1 (1.5)	4 (5.9)	2 (3.1)
GGT increased	0	1 (1.5)	1 (1.5)	0	5 (7.4)	2 (3.1)
Dental caries	1 (1.5)	3 (4.4)	0	2 (3.1)	5 (7.4)	0
Nausea	1 (1.5)	1 (1.5)	1 (1.5)	1 (1.5)	2 (2.9)	3 (4.6)
ALT increased	0	1 (1.5)	2 (3.1)	1 (1.5)	1 (1.5)	3 (4.6)
Constipation	0	1 (1.5)	1 (1.5)	0	1 (1.5)	3 (4.6)
Insomnia	1 (1.5)	1 (1.5)	1 (1.5)	1 (1.5)	1 (1.5)	3 (4.6)
Malaise	1 (1.5)	1 (1.5)	2 (3.1)	1 (1.5)	1 (1.5)	3 (4.6)
Hepatic function abnormal	1 (1.5)	0	2 (3.1)	1 (1.5)	0	3 (4.6)
Vertigo	0	0	1 (1.5)	0	0	3 (4.6)
Periodontal disease	0	0	1 (1.5)	0	2 (2.9)	2 (3.1)
Rash	1 (1.5)	2 (2.9)	1 (1.5)	1 (1.5)	2 (2.9)	2 (3.1)
Wound	0	0	2 (3.1)	0	2 (2.9)	2 (3.1)
AST increased	0	0	2 (3.1)	0	1 (1.5)	2 (3.1)
Contusion	0	0	1 (1.5)	0	1 (1.5)	2 (3.1)
Diarrhea	0	0	1 (1.5)	0	1 (1.5)	2 (3.1)
Herpes zoster	1 (1.5)	0	1 (1.5)	1 (1.5)	1 (1.5)	2 (3.1)
Liver function test increased	0	2 (2.9)	1 (1.5)	0	2 (2.9)	2 (3.1)
SARS-CoV-2 test positive	0	0	0	1 (1.5)	1 (1.5)	2 (3.1)
Cough	0	0	1 (1.5)	0	0	2 (3.1)
Dizziness	1 (1.5)	0	0	2 (3.1)	0	2 (3.1)
Ocular hypertension	0	0	1 (1.5)	0	0	2 (3.1)
Peripheral edema	2 (3.1)	0	1 (1.5)	3 (4.6)	0	2 (3.1)
Oropharyngeal discomfort	0	0	2 (3.1)	0	0	2 (3.1)
Orthostatic hypotension	0	0	0	0	0	2 (3.1)
Abdominal pain upper	1 (1.5)	2 (2.9)	1 (1.5)	1 (1.5)	2 (2.9)	1 (1.5)
Oropharyngeal pain	1 (1.5)	2 (2.9)	0	1 (1.5)	3 (4.4)	1 (1.5)
Oral herpes	0	0	0	0	2 (2.9)	1 (1.5)

Stomatitis	0	2 (2.9)	0	0	3 (4.4)	0
Animal bite	0	1 (1.5)	0	0	2 (2.9)	0
Cystitis	0	0	0	0	2 (2.9)	0
Dyspepsia	0	2 (2.9)	0	0	2 (2.9)	0
Epistaxis	0	2 (2.9)	0	0	2 (2.9)	0
Gastroenteritis	0	0	0	2 (3.1)	2 (2.9)	0
Hepatic enzyme increased	0	2 (2.9)	0	0	2 (2.9)	0
Hypertension	0	2 (2.9)	0	0	2 (2.9)	0
Large intestine polyp	0	1 (1.5)	0	0	2 (2.9)	0
Nasal vestibulitis	0	2 (2.9)	0	0	2 (2.9)	0
Noncardiac chest pain	0	1 (1.5)	0	0	2 (2.9)	0
Pain in extremity	1 (1.5)	1 (1.5)	0	1 (1.5)	2 (2.9)	0
Hordeolum	1 (1.5)	0	0	2 (3.1)	1 (1.5)	0
Immunization reaction	3 (4.6)	1 (1.5)	0	3 (4.6)	1 (1.5)	0

ALT, alanine aminotransferase; AST, aspartate aminotransferase; GGT, gamma-glutamyl transferase; IP, induction period; MP, maintenance period; TEAE, treatment-emergent adverse event.

Supplementary Table 8. Abnormal Electrocardiogram Values at Hour 6 in Patients With 6-Hour Cardiac Monitoring on Day 1 (induction period, safety population)

	**Placebo (N=65)**	**Ozanimod 0.46 mg (N=68)**	**Ozanimod 0.92 mg (N=65)**
n	24	21	22
QT >480 ms	0	0	0
QT >500 ms	0	0	0
QTcF >480 ms	0	1 (4.8)	0
QTcF >500 ms	0	1 (4.8)	0
Change from baseline in QT of >30 ms	1 (4.2)	4 (19.0)	7 (31.8)
Change from baseline in QT of >60 ms	0	1 (4.8)	0
Change from baseline in QTcF of >30 ms	0	1 (4.8)	1 (4.5)
Change from baseline in QTcF of >60 ms	0	1 (4.8)	0

Data are n (%).

QTcF, QT interval corrected for heart rate using Fridericia's formula.

Supplementary Table 9. Electrocardiogram Findings in the Induction and Maintenance Periods (safety population)

	IP			IP and MP		
	**Placebo (N=65)**	**Ozanimod 0.46 mg (N=68)**	**Ozanimod 0.92 mg (N=65)**	**Placebo (N=65)**	**Ozanimod 0.46 mg (N=68)**	**Ozanimod 0.92 mg (N=645)**
n	65	67	64	65	67	64
QT >480 ms	0	0	0	0	1 (1.5)	0
QT >500 ms	0	0	0	0	1 (1.5)	0
QTcF >480 ms	0	1 (1.5)	0	0	2 (3.0)	0
QTcF >500 ms	0	1 (1.5)	0	0	2 (3.0)	0
Change from baseline in QT of >30 ms	6 (9.2)	15 (22.4)	14 (21.9)	6 (9.2)	18 (26.9)	17 (26.6)
Change from baseline in QT of >60 ms	0	2 (3.0)	0	0	4 (6.0)	0
Change from baseline in QTcF of >30 ms	0	3 (4.5)	3 (4.7)	0	5 (7.5)	4 (6.3)
Change from baseline in QTcF of >60 ms	0	1 (1.5)	0	0	2 (3.0)	0

Data are n (%).

IP, induction period; MP, maintenance period; QTcF, QT interval corrected for heart rate using Fridericia's formula.

Supplementary Table 10. Mean (standard deviation) Heart Rate and Change From Baseline in Heart Rate During Cardiac Monitoring on Study Day 1 Hours 1–6 (induction period, safety population)

	**Placebo (N=65)**	**Ozanimod 0.46 mg (N=68)**	**Ozanimod 0.92 mg (N=65)**
Pulse rate, supine (bpm), n	24	21	22
Mean (SD) HR			
Baseline	68.5 (10.0)	71.3 (10.3)	69.1 (8.8)
Hour 1	66.7 (9.1)	69.3 (9.9)	73.0 (9.2)
Hour 2	69.4 (9.5)	72.3 (10.4)	70.1 (8.9)
Hour 3	70.0 (9.5)	72.2 (9.4)	67.7 (10.3)
Hour 4	68.5 (7.6)	70.1 (9.0)	70.2 (8.0)
Hour 5	66.9 (7.1)	69.3 (9.6)	68.2 (9.5)
Hour 6	67.8 (9.9)	68.3 (8.6)	67.2 (8.2)
Mean (SD) change from baseline in HR			
Hour 1	−0.5 (5.6)	−2.2 (7.5)	0.4 (8.5)
Hour 2	2.2 (7.9)	0.7 (8.6)	−2.4 (7.3)
Hour 3	2.8 (8.5)	0.7 (8.4)	−4.8 (7.3)
Hour 4	1.3 (7.4)	−1.5 (9.7)	−2.4 (8.3)
Hour 5	−0.3 (6.2)	−2.3 (9.7)	−4.3 (7.7)
Hour 6	0.6 (8.0)	−3.3 (9.1)	−5.3 (7.8)
Pulse rate, standing (bpm), n	24	21	22
Mean (SD) HR			
Baseline	81.0 (14.0)	85.0 (12.2)	82.8 (11.3)
Hour 1	78.6 (11.6)	82.5 (12.4)	84.5 (9.5)
Hour 2	84.4 (12.5)	85.8 (14.7)	80.2 (9.7)
Hour 3	87.7 (13.1)	83.7 (13.1)	80.4 (8.7)
Hour 4	85.2 (9.4)	82.2 (10.1)	80.8 (9.6)
Hour 5	80.4 (9.6)	80.2 (9.7)	79.6 (11.3)
Hour 6	79.3 (9.7)	80.1 (9.3)	76.0 (9.4)
Mean (SD) change from baseline in HR			
Hour 1	−1.3 (7.8)	−1.4 (9.3)	1.9 (9.8)
Hour 2	4.5 (10.7)	1.9 (11.6)	−2.4 (8.7)
Hour 3	7.9 (11.8)	−0.2 (10.4)	−2.2 (8.5)
Hour 4	5.3 (10.2)	−1.7 (10.9)	−1.8 (9.6)
Hour 5	0.5 (10.3)	−3.7 (9.8)	−2.9 (12.0)
Hour 6	−0.6 (8.7)	−3.8 (8.6)	−6.5 (10.6)

bpm, beats per minute; HR, heart rate; SD, standard deviation.

Supplementary Figure 1. Study design of the phase 3 Japan-True North study.

[a]The maintenance period was initially planned as 52 weeks but was shortened to 40 weeks in an amendment to the protocol. This did not affect the timing of efficacy endpoints at week 52 for the maintenance period. [b]Patients were stratified by corticosteroid use at screening (yes or no) and prior biologic use (yes or no). [c]Ozanimod was initiated at a dose of 0.23 mg for 4 days, then ozanimod 0.46 mg for 3 days, followed thereafter by the assigned treatment level (ie, ozanimod 0.46 mg or ozanimod 0.92 mg). [d]Patients who did not achieve clinical response (defined as a reduction from baseline in the complete Mayo score ≥3 points and ≥30% and a reduction from baseline in the RBS of ≥1 point or an absolute RBS of ≤1 point) at week 12. [e]Disease relapse: increase in UC disease activity as defined by an increase in partial Mayo score ≥2 points compared with the week 12 partial Mayo score with an absolute partial Mayo score ≥4 points, an endoscopic subscore of ≥2 points, and exclusion of other causes of an increase in disease activity unrelated to underlying UC (eg, infections, change in medication). OLE, open-label extension; RBS, rectal bleeding subscore; UC, ulcerative colitis.

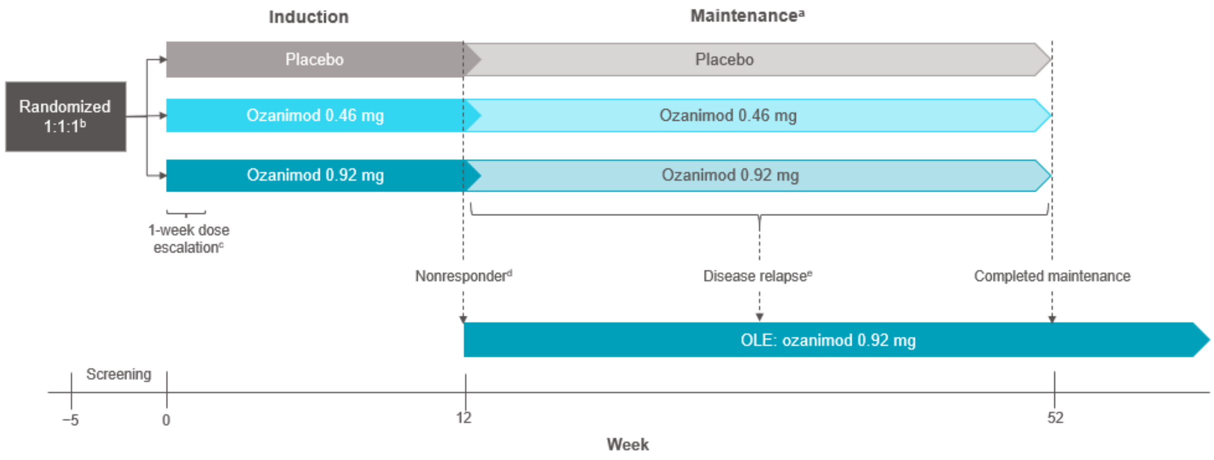

Supplementary Figure 2. Additional secondary and exploratory endpoints at weeks 12 and 52. Nonresponder imputation approach was used for handling of missing data. Weighted differences, 95% CIs, and *P* values for comparison between groups were based on the Cochran-Mantel-Haenszel test and were stratified by prior biologic agents and corticosteroid use (yes/no). [a]Clinical response: A reduction from baseline in the 9-point Mayo score of ≥2 points and ≥35%, and a reduction from baseline in the RBS of ≥1 point or an absolute RBS of ≤1 point. [b]Clinical remission: complete Mayo score of ≤2 points and with no individual subscore of >1 point. [c]Clinical remission: SFS = 0 or 1 (without a requirement of a decrease of ≥1 point from the baseline SFS), RBS = 0, and endoscopy subscore = 0 or 1. [d]Histologic remission: Geboes score <2.0. CI, confidence interval; RBS, rectal bleeding subscore; SFS, stool frequency subscore.

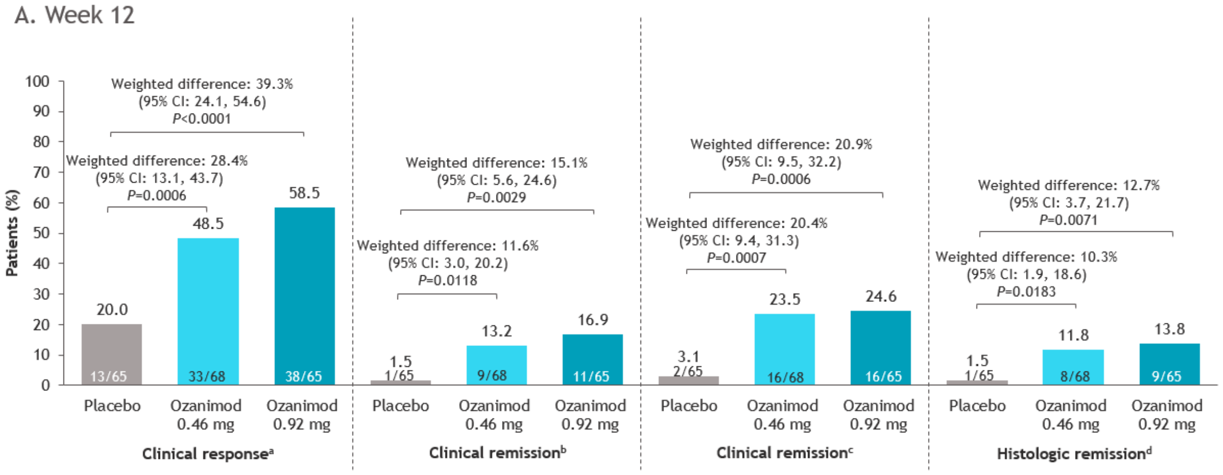

B. Week 52

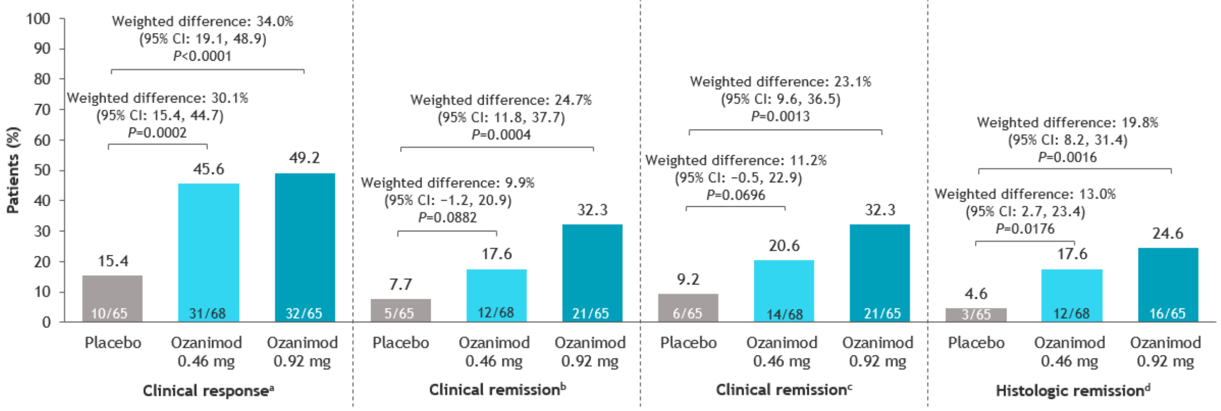

Patients (%)
100
90
80
70
60
50
40
30
20
10
0
Weighted difference: 34.0%
(95% CI: 19.1, 48.9)
P<0.0001
Weighted difference: 30.1%
(95% CI: 15.4, 44.7)
P=0.0002
15.4
45.6
49.2
10/65
31/68
32/65
Placebo
Ozanimod 0.46 mg
Ozanimod 0.92 mg
Clinical response[a]
Weighted difference: 24.7%
(95% CI: 11.8, 37.7)
P=0.0004
Weighted difference: 9.9%
(95% CI: −1.2, 20.9)
P=0.0882
7.7
17.6
32.3
5/65
12/68
21/65
Placebo
Ozanimod 0.46 mg
Ozanimod 0.92 mg
Clinical remission[b]
Weighted difference: 23.1%
(95% CI: 9.6, 36.5)
P=0.0013
Weighted difference: 11.2%
(95% CI: −0.5, 22.9)
P=0.0696
9.2
20.6
32.3
6/65
14/68
21/65
Placebo
Ozanimod 0.46 mg
Ozanimod 0.92 mg
Clinical remission[c]
Weighted difference: 19.8%
(95% CI: 8.2, 31.4)
P=0.0016
Weighted difference: 13.0%
(95% CI: 2.7, 23.4)
P=0.0176
4.6
17.6
24.6
3/65
12/68
16/65
Placebo
Ozanimod 0.46 mg
Ozanimod 0.92 mg
Histologic remission[d]

Supplementary Figure 3. Clinical response at week 12 by baseline characteristic subgroups. Nonresponder imputation approach is used for handling missing data. Treatment differences and *P* values for comparison between the active and placebo groups are based on the Cochran-Mantel-Haenszel test, stratified by prior biologic agents and CS use (yes or no). If the subgroup is the stratification factor, the Cochran-Mantel-Haenszel test is not stratified by this subgroup factor. 5-ASA, 5-aminosalicylic acid; ALC, absolute lymphocyte count; BL, baseline; CI, confidence interval; CS, corticosteroid; UC, ulcerative colitis.

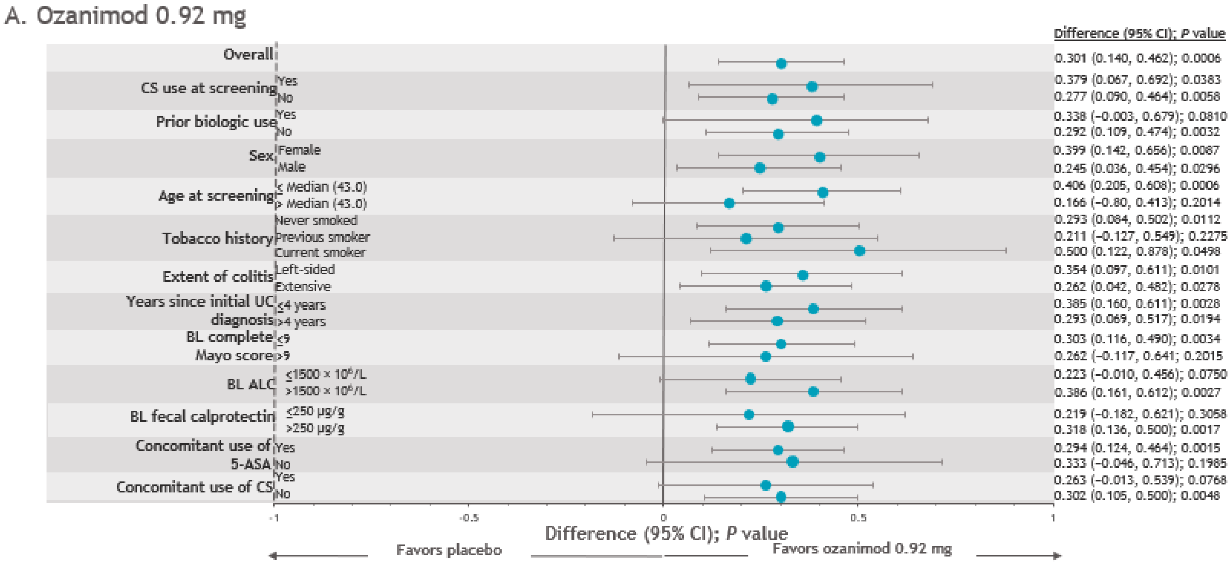

B. Ozanimod 0.46 mg

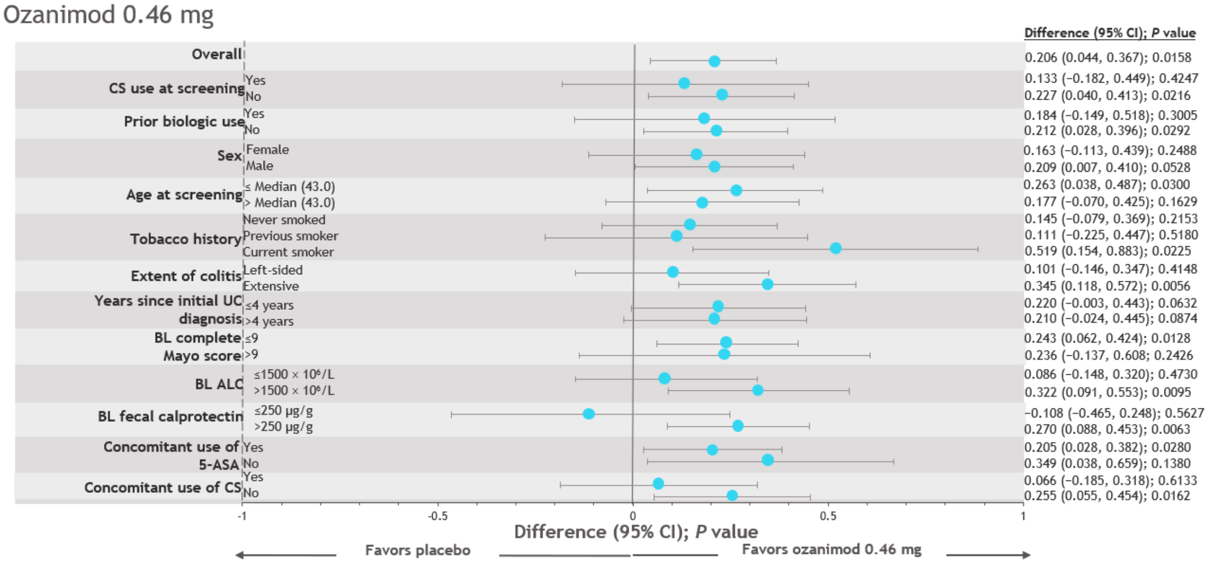

Supplementary Figure 4. Changes in absolute lymphocyte count over time. ALC, absolute lymphocyte count; SD, standard deviation; W, week.

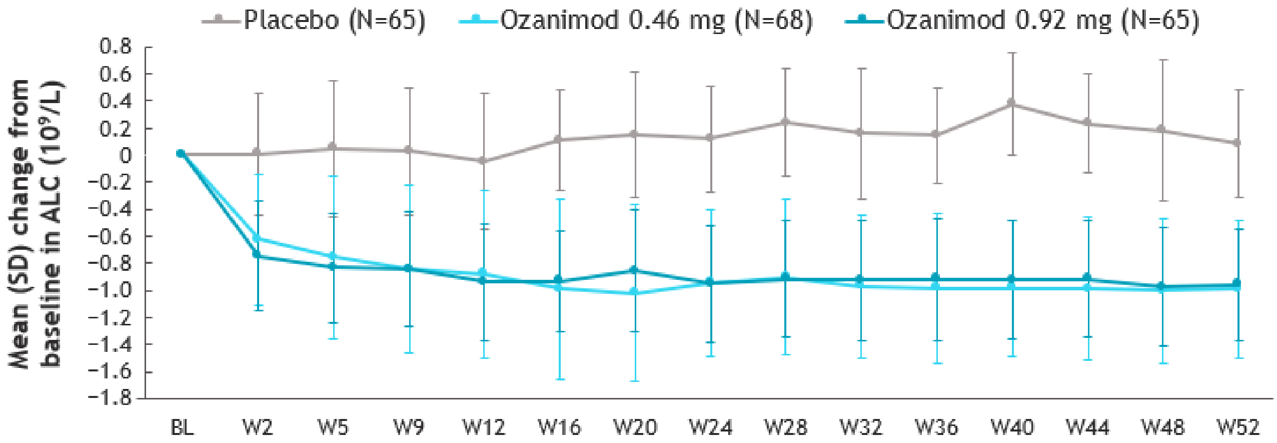

n															
Placebo	65	62	61	59	58	17	16	15	14	13	13	13	13	13	12
Ozanimod 0.46 mg	68	63	61	60	59	37	37	36	36	36	36	36	35	35	34
Ozanimod 0.92 mg	65	62	61	59	59	39	39	38	37	36	36	36	36	35	35

Plain Language Summary

Publication Plain Language Summary

Ozanimod Was Effective and Well Tolerated in Japanese Patients With Moderate to Severe Ulcerative Colitis

The full title of the article is “Once-Daily Oral Ozanimod for Japanese Patients With Ulcerative Colitis: Results From the Phase 2/3 J-True North Study”

You can find the full article here: [XXX]. You can access the full article for free

This publication plain language summary has been developed to accompany the article and is not intended for any other use.

What did this study look at?

Ulcerative colitis: What is it?

- Ulcerative colitis is a disease in which the lining of the colon and rectum becomes inflamed. Symptoms of ulcerative colitis include blood in the stool, diarrhea, and a feeling that you need to pass stools even though your bowels are already empty

People with untreated ulcerative colitis have a higher risk for

Anxiety and depression

Cancer of the colon and rectum

This disease can also negatively affect a person’s job and social interactions

- To treat ulcerative colitis, doctors commonly prescribe anti-inflammatory medications, such as aminosalicylates and corticosteroids, as the first step. Medications known as immunomodulators, which reduce inflammation by decreasing the immune system response that starts the process of inflammation, may also be prescribed. If those medications do not work, medications known as biologics and Janus kinase inhibitors may be prescribed

The study: What is it about and why was it done?

- The efficacy and safety of ozanimod have not yet been evaluated in a large number of Japanese people
- Therefore, researchers conducted this J-True North study to see how once-daily ozanimod 0.46 mg or ozanimod 0.92 mg works in Japanese people (hereafter referred to as participants) with moderate to severe ulcerative colitis and to see what side effects it caused
- Based on findings from J-True North, once-daily ozanimod 0.92 mg was approved in Japan in December 2024 for the treatment of moderate to severe ulcerative colitis in participants who have had an inadequate response to standard treatments

Ozanimod: How does it work?

- Ozanimod is a sphingosine 1-phosphate (S1P) receptor 1 and 5 modulator that works to reduce inflammation by preventing lymphocytes, a kind of white blood cell, from moving into inflamed tissues

Ozanimod is a capsule taken by mouth

Participants who start ozanimod need to slowly increase the dose of ozanimod over a week to reach the once-daily dose of 0.92 mg

Ozanimod 0.23 mg once daily	Ozanimod 0.46 mg once daily	Ozanimod 0.92 mg once daily
Days 1-4	Days 5-7	Day 8 and beyond

The dose of ozanimod is increased slowly to reduce the chance of side effects on the heart.

- Ozanimod is approved to treat moderate to severe ulcerative colitis and multiple sclerosis in the United States and several other countries

The summary: What will this cover?

- This plain language summary describes a clinical study called J-True North, which looked at the effects of ozanimod in Japanese people with moderate to severe ulcerative colitis. This study compared changes in symptoms between people who took placebo and ozanimod to see if ozanimod worked. The placebo looked like ozanimod but did not have the active medication in it. Researchers also studied the side effects of ozanimod. This allowed them to determine whether ozanimod is safe and if it improved symptoms, such as blood in the stool and diarrhea

How was the study done?

Study start and end date
June 3, 2019, to August 28, 2023

Phase of study
Phase 2/3

Study status
Results reported within the study are final

Key inclusion criteria

- Japanese adults (18-75 years old) with ulcerative colitis for at least the past 3 months
- These adults must have previously taken aminosalicylates or corticosteroids to treat their ulcerative colitis

Key exclusion criteria

- People were excluded if they had serious inflammation of a large portion of their colon, known as severe extensive colitis, or if they had Crohn's disease, certain heart conditions, or a history of type 1 diabetes or uncontrolled type 2 diabetes

Randomization: Participants with ulcerative colitis were randomly assigned (or "randomized") to take once-daily ozanimod 0.46 mg, ozanimod 0.92 mg, or placebo

The placebo looks like ozanimod, but it does not have any medicine in it

This study was "blinded," meaning that participants in the study and the researchers did not know who took ozanimod and who took placebo

Screening
(5 weeks)

Researchers checked to see whether people with ulcerative colitis could take part in this study based on the study's inclusion and exclusion criteria

Induction period
(12 weeks)

Participants with ulcerative colitis took ozanimod or placebo for 12 weeks

After the 12 weeks, participants with ulcerative colitis who had clinical response, meaning whose symptoms were better, could enter the maintenance period of the study

Maintenance period
(40 weeks)

Participants with ulcerative colitis continued to take the same medicine (ozanimod or placebo) as they did during the induction period for an additional 40 weeks

Week −5 | 0 | 12 | 52

In this study, researchers determined whether people with ulcerative colitis had clinical response by seeing whether their symptoms improved (for example, by having less blood in the stool, less diarrhea, or less abdominal pain) and by using a small camera to see whether the colon looked less swollen

- The study looked at the percentages of participants with clinical response after 12 weeks of treatment and after 52 weeks of taking ozanimod
- These percentages of participants with clinical response after taking ozanimod were compared with the percentages of participants with clinical response after taking placebo
- Researchers also looked at the side effects that participants had during the study

Who took part in this study?

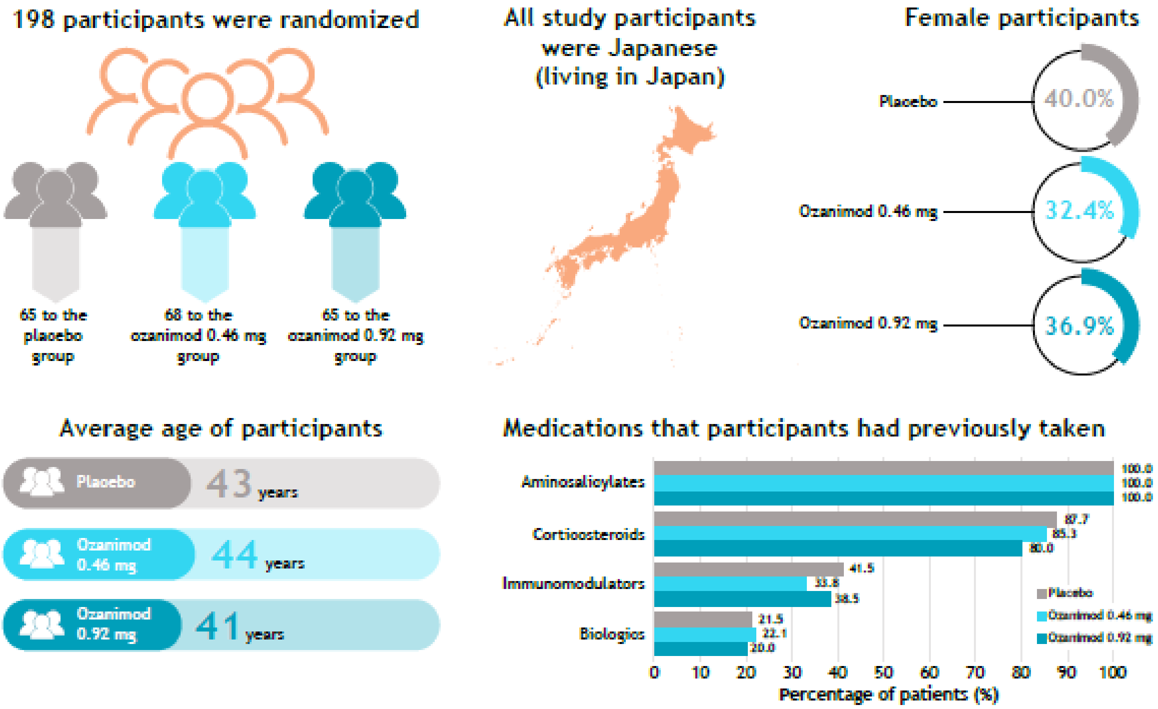

What were the results of the study?

- After taking ozanimod for 12 weeks (either dose), more than half of the participants had clinical response compared with less than one-third of those in the placebo group

32.3%
52.9%
61.5%

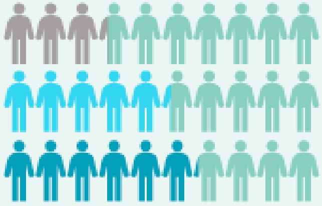

- After taking ozanimod for 52 weeks (either dose), almost half of the people had clinical response compared with only 16.9% in the placebo group

16.9%
47.1%
49.2%

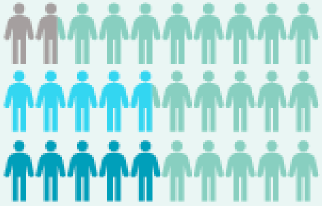

- ≥80% of participants who achieved clinical response receiving ozanimod 0.46 mg or ozanimod 0.92 mg at the end of the induction period showed clinical response at the end of the maintenance period

≥80%

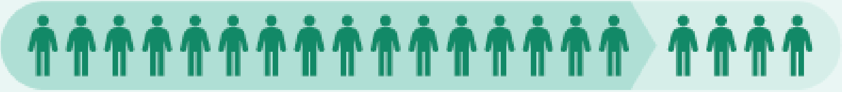

- In the induction and maintenance periods of the study, the following were the most common side effects in participants taking ozanimod:
- Researchers were interested in knowing whether participants taking ozanimod had side effects seen in patients who took S1P modulators, the same class of medications as ozanimod

	Cold symptoms	Fever	Headache	Low heart rate	Cancer	Shingles	Swelling in part of the retina
Placebo	9.2%	4.6%	6.2%	None	None	1.5%	None
Ozanimod 0.46 mg	14.7%	16.2%	11.8%	None	None	1.5%	None
Ozanimod 0.92 mg	13.8%	10.8%	9.2%	None	None	3.1%	1.5%

What were the study's main conclusions?

- Ozanimod was effective and well tolerated as a once-daily oral medication in Japanese participants with moderate to severe ulcerative colitis
- This Japanese clinical study was the first large-scale study examining ozanimod in an Asian population. The results suggest that ozanimod is effective and safe for Asians
- The efficacy and safety results of ozanimod in the J-True North study were similar to the findings from a global study of ozanimod called True North

Who sponsored this study?

This clinical study was sponsored by Bristol Myers Squibb, which thanks everybody who participated in the study

Where can I find additional information?

The original article was published in the medical journal *Gastro Hep Advances*. Please refer to the original article for complete author-disclosure information

You can find more information about this study here:
Study Details | To Evaluate Efficacy and Long-term Safety of Ozanimod in Japanese Subjects With Moderately to Severely Active Ulcerative Colitis | https://clinicaltrials.gov/study/NCT03915769

プレーンランゲージサマリー

中等症から重症の日本人潰瘍性大腸炎患者においてオザニモドの有効性と良好な忍容性が示されました

論文の題名：Once-Daily Oral Ozanimod for Japanese Patients With Ulcerative Colitis: Results From the Phase 2/3 J-True North Study

論文の全文は [XXX] から無料でご覧いただけます

このプレーンランゲージサマリーは論文の要約として作成されたもので、その他の用途での使用を意図したものではありません。

この試験では何を調べましたか?

潰瘍性大腸炎とは？

- 潰瘍性大腸炎とは、結腸や直腸の粘膜に炎症が生じる疾患です。症状には血便や下痢などがあり、排便後でも便意を感じることがあります

治療していない潰瘍性大腸炎の患者では、以下を発症するリスクが高まります

不安や抑うつ

結腸・直腸がん

潰瘍性大腸炎は仕事や社会的交流にも悪影響を及ぼすことがあります

- 潰瘍性大腸炎の治療には、最初にアミノサリチル酸製剤やコルチコステロイドなどの炎症を抑える薬が一般的に処方されます。炎症の起点となる免疫反応を低下させる目的で免疫調節薬が処方されることもあります。これらの薬で効果がなければ生物学的製剤や JAK 阻害薬と呼ばれる薬が処方されることがあります

この試験では何を調べましたか、なぜ行われたのですか？

- これまでにオザニモドの有効性と安全性が日本人で大規模に評価されたことはありません
- そこで研究者は、中等症から重症の日本人潰瘍性大腸炎患者（以下、参加者と呼びます）を対象にこの J-True North 試験を行い、オザニモドを 0.46 mg または 0.92 mg で 1 日 1 回投与した場合、どのような効果を示し、どのような副作用が現れるかを確かめました
- J-True North 試験の結果を基に、オザニモド 0.92 mg の 1 日 1 回投与が、既存治療で効果が不十分であった中等症から重症の潰瘍性大腸炎の治療薬として、2024 年 12 月に日本で承認されました

オザニモドはどのように作用するのですか？

- オザニモドはスフィンゴシン 1- リン酸（S1P）受容体 1 および 5 に対する調節薬で、白血球の一種であるリンパ球の炎症組織への移行を阻害することで炎症を抑制します

オザニモドはカプセル剤の飲み薬です

オザニモドは 1 日 1 回の投与量が 0.92 mg に達するまで 1 週間かけてゆっくりと投与量を増やしていく必要があります

オザニモド 0.23 mg	オザニモド 0.46 mg	オザニモド 0.92 mg
1 日 1 回	1 日 1 回	1 日 1 回
1 ～ 4 日目	5 ～ 7 日目	8 日目以降

心臓への副作用を減らす目的でオザニモドの用量はゆっくり増やしていきます。

- オザニモドは中等症から重症の潰瘍性大腸炎および多発性硬化症の治療薬として米国をはじめ諸外国で承認されています

この文書では何について書かれていますか？

- このプレーンランゲージサマリーは、中等症から重症の日本人潰瘍性大腸炎患者を対象にオザニモドの効果を検討した臨床試験（J-True North 試験）について説明しています。この試験では、オザニモドの効果を確認するために、プラセボを服用した患者とオザニモドを服用した患者で症状の変化を比較しました。プラセボはオザニモドのような見た目で有効成分は入っていません。研究者はオザニモドの副作用についても検討しました。試験により、オザニモドが安全であるかどうか、血便や下痢などの症状が改善されるかどうかを調べることができました

試験はどのように行われましたか?

試験の開始日と終了日
2019 年 6 月 3 日から 2023 年 8 月 28 日まで

試験の相
第 2/3 相

試験の状況
試験内で最終結果が報告されています

主な選択基準

- 過去 3 ヵ月間以上前に潰瘍性大腸炎と診断された日本人の成人患者（18 ～ 75 歳）
- このうち、治療のためにアミノサリチル酸製剤またはコルチコステロイドの使用歴がある患者

主な除外基準

- 大腸の大部分に重篤な炎症がある、重症で広範囲に及ぶ大腸炎患者、クローン病または特定の心臓疾患を持つ患者、もしくは 1 型糖尿病またはコントロール不良の 2 型糖尿病の既往がある患者

ランダム化：試験に参加した潰瘍性大腸炎患者は、1日1回のオザニモド 0.46 mg 投与群、オザニモド 0.92 mg 投与群、またはプラセボ投与群のいずれかにランダムに割り付けられました

プラセボはオザニモドのような見た目ですが薬の成分は入っていません

この試験は「盲検化」されており、誰がオザニモドを服用し、誰がプラセボを服用したかは、試験に参加した患者にも研究者にも知らされませんでした

スクリーニング（5 週間）
潰瘍性大腸炎の患者がこの試験に参加できるかどうかを、選択基準と除外基準に基づいて研究者が確認しました

導入期（12 週間）
試験に参加した患者はオザニモドまたはプラセボを 12 週間服用しました
12 週間後、臨床的改善が認められた、つまり症状が改善した患者は試験の維持期に進むことができました

維持期（40 週間）
参加者は、導入期と同じ薬（オザニモドまたはプラセボ）の服用をさらに 40 週間続けました

週 –5　0　12　52

この試験で研究者は、潰瘍性大腸炎の症状が改善したか（血便が減った、下痢が減った、腹痛が減ったなど）、さらに内視鏡を使って大腸の状態を見ることによって、臨床的改善を得られたかどうかを判定しました

- この試験では、オザニモドを 12 週間および 52 週間服用したあとに臨床的改善が認められた参加者の割合を調べました
- オザニモドの服用後に臨床的改善が認められた参加者の割合を、プラセボの服用後に臨床的改善が認められた参加者の割合と比較しました
- さらに研究者は試験中に参加者に発現した副作用についても調べました

誰がこの試験に参加しましたか?

198 名がランダム化されました

プラセボ群 65 名
オザニモド 0.46 mg 群 68 名
オザニモド 0.92 mg 群 65 名

試験参加者全員が日本人（日本在住）

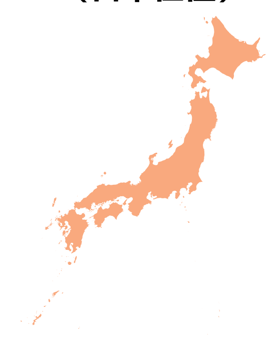

女性参加者

プラセボ 40.0%
オザニモド 0.46 mg 32.4%
オザニモド 0.92 mg 36.9%

参加者の平均年齢

プラセボ 43 歳
オザニモド 0.46 mg 44 歳
オザニモド 0.92 mg 41 歳

参加者がこれまでに使用した薬

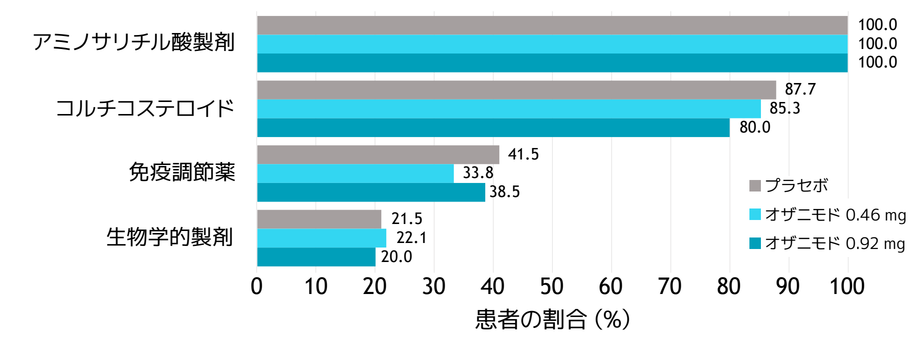

試験の結果は？

- オザニモドを 12 週間服用した後に臨床的改善が認められた参加者は、（どちらの用量でも）半数を超えたのに対し、プラセボ群では 3 分の 1 未満でした

32.3%
52.9%
61.5%

- オザニモドを 52 週間服用した後に臨床的改善が認められた参加者は、（どちらの用量でも）ほぼ半数であったのに対し、プラセボ群では 16.9% でした

16.9%
47.1%
49.2%

- 導入期の終了時点で、オザニモド 0.46 mg またはオザニモド 0.92 mg を服用して臨床的改善を得た参加者の 80% 以上が維持期の終了時点で臨床的改善を示しました

≥80%

- 試験の導入期および維持期において、オザニモドを服用した参加者に多く見られた副作用は以下の通りです
- 研究者は、オザニモドを服用している参加者に、オザニモドと類似の S1P 受容体調節薬で見られた副作用が現れるかどうかに関心がありました

	風邪症状	発熱	頭痛	心拍数低下	がん	帯状疱疹	黄斑浮腫
プラセボ	9.2%	4.6%	6.2%	なし	なし	1.5%	なし
オザニモド 0.46 mg	14.7%	16.2%	11.8%	なし	なし	1.5%	なし
オザニモド 0.92 mg	13.8%	10.8%	9.2%	なし	なし	3.1%	1.5%

この試験の主な結論は？

- オザニモドは中等症から重症の潰瘍性大腸炎を持つ日本人患者において 1 日 1 回の経口投与で有効であり、忍容性は良好でした
- 日本人を対象にしたこのオザニモドの臨床試験はアジア人集団において初めて大規模に検討した試験であり、その結果からアジア人におけるオザニモドの有効性と安全性が示唆されました
- オザニモドの有効性と安全性に関して J-True North 試験では、True North 試験と呼ばれるオザニモドの海外で実施された治験と同様の結果が得られました

この試験は誰が行いましたか?

この臨床試験はブリストル・マイヤーズ スクイブ株式会社が行いました。この試験に参加して下さった皆様に感謝申し上げます

追加の情報はどこで見られますか?

原著論文は医学雑誌 *Gastro Hep Advances* に掲載されました。著者の利益相反の開示情報については、原著論文を参照ください

この試験の詳細についてはこちらをご覧ください：
試験詳細 | To Evaluate Efficacy and Long-term Safety of Ozanimod in Japanese Subjects With Moderately to Severely Active Ulcerative Colitis | https://clinicaltrials.gov/study/NCT03915769
