# Supplementary material for: Once-Daily Oral Ozanimod for Japanese Patients With Ulcerative Colitis: Results From the Phase 2/3 J-True North Study
Source: Gastro Hep Adv. 2025 Sep 16;5(1):100812. doi: 10.1016/j.gastha.2025.100812 (PMC12630022; doi:10.1016/j.gastha.2025.100812)
Supplement: Supplementary material [file mmc3.pdf]

プレーンランゲージサマリー

中等症から重症の日本人潰瘍性大腸炎患者において オザニモドの有効性と良好な忍容性が示されました

論文の題名：Once-Daily Oral Ozanimod for Japanese Patients With Ulcerative Colitis: Results From the Phase 2/3 J-True North Study

論文の全文は [XXX] から無料でご覧いただけます

このプレーンランゲージサマリーは論文の要約として作成されたもので、その他の用途での使用を意図したものではありません。

この試験では何を調べましたか？

潰瘍性大腸炎とは？

- 潰瘍性大腸炎とは、結腸や直腸の粘膜に炎症が生じる疾患です。症状には血便や下痢などがあり、排便後でも便意を感じることがあります

治療していない潰瘍性大腸炎の患者では、以下を発症するリスクが高まります

不安や抑うつ
結腸・直腸がん

潰瘍性大腸炎は仕事や社会的交流にも悪影響を及ぼすことがあります

- 潰瘍性大腸炎の治療には、最初にアミノサリチル酸製剤やコルチコステロイドなどの炎症を抑える薬が一般的に処方されます。炎症の起点となる免疫反応を低下させる目的で免疫調節薬が処方されることもあります。これらの薬で効果がなければ生物学的製剤や JAK 阻害薬と呼ばれる薬が処方されることがあります

この試験では何を調べましたか、なぜ行われたのですか？

- これまでにオザニモドの有効性と安全性が日本人で大規模に評価されたことはありません
- そこで研究者は、中等症から重症の日本人潰瘍性大腸炎患者（以下、参加者と呼びます）を対象にこの J-True North 試験を行い、オザニモドを 0.46 mg または 0.92 mg で 1 日 1 回投与した場合、どのような効果を示し、どのような副作用が現れるかを確かめました
- J-True North 試験の結果を基に、オザニモド 0.92 mg の 1 日 1 回投与が、既存治療で効果が不十分であった中等症から重症の潰瘍性大腸炎の治療薬として、2024 年 12 月に日本で承認されました

オザニモドはどのように作用するのですか？

- オザニモドはスフィンゴシン 1- リン酸（S1P）受容体 1 および 5 に対する調節薬で、白血球の一種であるリンパ球の炎症組織への移行を阻害することで炎症を抑制します

オザニモドはカプセル剤の飲み薬です

オザニモドは 1 日 1 回の投与量が 0.92 mg に達するまで 1 週間かけてゆっくりと投与量を増やしていく必要があります

オザニモド 0.23 mg	オザニモド 0.46 mg	オザニモド 0.92 mg
1 日 1 回	1 日 1 回	1 日 1 回
1 ～ 4 日目	5 ～ 7 日目	8 日目以降

心臓への副作用を減らす目的でオザニモドの用量はゆっくり増やしていきます。

- オザニモドは中等症から重症の潰瘍性大腸炎および多発性硬化症の治療薬として米国をはじめ諸外国で承認されています

この文書では何について書かれていますか？

- このプレーンランゲージサマリーは、中等症から重症の日本人潰瘍性大腸炎患者を対象にオザニモドの効果を検討した臨床試験（J-True North 試験）について説明しています。この試験では、オザニモドの効果を確認するために、プラセボを服用した患者とオザニモドを服用した患者で症状の変化を比較しました。プラセボはオザニモドのような見た目で有効成分は入っていません。研究者はオザニモドの副作用についても検討しました。試験により、オザニモドが安全であるかどうか、血便や下痢などの症状が改善されるかどうかを調べることができました

試験はどのように行われましたか？

試験の開始日と終了日
2019 年 6 月 3 日から 2023 年 8 月 28 日まで

試験の相
第 2/3 相

試験の状況
試験内で最終結果が報告されています

主な選択基準

- 過去 3 ヵ月間以上前に潰瘍性大腸炎と診断された日本人の成人患者（18 ～ 75 歳）
- このうち、治療のためにアミノサリチル酸製剤またはコルチコステロイドの使用歴がある患者

主な除外基準

- 大腸の大部分に重篤な炎症がある、重症で広範囲に及ぶ大腸炎患者、クローン病または特定の心臓疾患を持つ患者、もしくは 1 型糖尿病またはコントロール不良の 2 型糖尿病の既往がある患者

ランダム化：試験に参加した潰瘍性大腸炎患者は、1 日 1 回のオザニモド 0.46 mg 投与群、オザニモド 0.92 mg 投与群、またはプラセボ投与群のいずれかにランダムに割り付けられました

プラセボはオザニモドのような見た目ですが薬の成分は入っていません

この試験は「盲検化」されており、誰がオザニモドを服用し、誰がプラセボを服用したかは、試験に参加した患者にも研究者にも知らされませんでした

スクリーニング（5 週間）
潰瘍性大腸炎の患者がこの試験に参加できるかどうかを、選択基準と除外基準に基づいて研究者が確認しました

導入期（12 週間）
試験に参加した患者はオザニモドまたはプラセボを 12 週間服用しました
12 週間後、臨床的改善が認められた、つまり症状が改善した患者は試験の維持期に進むことができました

維持期（40 週間）
参加者は、導入期と同じ薬（オザニモドまたはプラセボ）の服用をさらに 40 週間続けました

週 -5 0 12 52

この試験で研究者は、潰瘍性大腸炎の症状が改善したか（血便が減った、下痢が減った、腹痛が減ったなど）、さらに内視鏡を使って大腸の状態を見ることによって、臨床的改善を得られたかどうかを判定しました

- この試験では、オザニモドを 12 週間および 52 週間服用したあとに臨床的改善が認められた参加者の割合を調べました
- オザニモドの服用後に臨床的改善が認められた参加者の割合を、プラセボの服用後に臨床的改善が認められた参加者の割合と比較しました
- さらに研究者は試験中に参加者に発現した副作用についても調べました

誰がこの試験に参加しましたか？

198 名がランダム化されました

プラセボ群 65 名
オザニモド 0.46 mg 群 68 名
オザニモド 0.92 mg 群 65 名

試験参加者全員が日本人（日本在住）

女性参加者

プラセボ 40.0%
オザニモド 0.46 mg 32.4%
オザニモド 0.92 mg 36.9%

参加者の平均年齢

プラセボ 43 歳
オザニモド 0.46 mg 44 歳
オザニモド 0.92 mg 41 歳

参加者がこれまでに使用した薬（患者の割合 (%)）

	プラセボ	オザニモド 0.46 mg	オザニモド 0.92 mg
アミノサリチル酸製剤	100.0	100.0	100.0
コルチコステロイド	87.7	85.3	80.0
免疫調節薬	41.5	33.8	38.5
生物学的製剤	21.5	22.1	20.0

試験の結果は？

- オザニモドを 12 週間服用した後に臨床的改善が認められた参加者は、（どちらの用量でも）半数を超えたのに対し、プラセボ群では 3 分の 1 未満でした

32.3%
52.9%
61.5%

- オザニモドを 52 週間服用した後に臨床的改善が認められた参加者は、（どちらの用量でも）ほぼ半数であったのに対し、プラセボ群では 16.9% でした

16.9%
47.1%
49.2%

- 導入期の終了時点で、オザニモド 0.46 mg またはオザニモド 0.92 mg を服用して臨床的改善を得た参加者の 80% 以上が維持期の終了時点で臨床的改善を示しました

≥80%

- 試験の導入期および維持期において、オザニモドを服用した参加者に多く見られた副作用は以下の通りです
- 研究者は、オザニモドを服用している参加者に、オザニモドと類似の S1P 受容体調節薬で見られた副作用が現れるかどうかに関心がありました

	風邪症状	発熱	頭痛	心拍数低下	がん	帯状疱疹	黄斑浮腫
プラセボ	9.2%	4.6%	6.2%	なし	なし	1.5%	なし
オザニモド 0.46 mg	14.7%	16.2%	11.8%	なし	なし	1.5%	なし
オザニモド 0.92 mg	13.8%	10.8%	9.2%	なし	なし	3.1%	1.5%

この試験の主な結論は？

- オザニモドは中等症から重症の潰瘍性大腸炎を持つ日本人患者において 1 日 1 回の経口投与で有効であり、忍容性は良好でした
- 日本人を対象にしたこのオザニモドの臨床試験はアジア人集団において初めて大規模に検討した試験であり、その結果からアジア人におけるオザニモドの有効性と安全性が示唆されました
- オザニモドの有効性と安全性に関して J-True North 試験では、True North 試験と呼ばれるオザニモドの海外で実施された治験と同様の結果が得られました

この試験は誰が行いましたか？

この臨床試験はブリストル・マイヤーズ スクイブ株式会社が行いました。この試験に参加して下さった皆様に感謝申し上げます

追加の情報はどこで見られますか？

原著論文は医学雑誌 *Gastro Hep Advances* に掲載されました。著者の利益相反の開示情報については、原著論文を参照ください

この試験の詳細についてはこちらをご覧ください：
試験詳細 | To Evaluate Efficacy and Long-term Safety of Ozanimod in Japanese Subjects With Moderately to Severely Active Ulcerative Colitis | https://clinicaltrials.gov/study/NCT03915769
